# ATF3 Deficiency Exacerbates Ageing‐Induced Atherosclerosis and Clinical Intervention Strategy

**DOI:** 10.1002/advs.202502249

**Published:** 2025-07-11

**Authors:** Hao Nie, Tianyi Ji, Zixin Wan, Jie Huang, Han Li, Lingjiao Zou, Zhen Yang, Jiarui Li, Yuqi Guan, Lei Ruan, Jinhua Yan, Cuntai Zhang

**Affiliations:** ^1^ Department of Geriatrics, Key Laboratory of Vascular ageing Ministry of Education Tongji Hospital of Tongji Medical College Huazhong University of Science and Technology Wuhan P. R. China; ^2^ Department of Geriatrics Tongji Hospital of Tongji Medical College Huazhong University of Science and Technology Wuhan P. R. China

**Keywords:** activated transcription factor 3, atherosclerosis, autophagy, terazosin, vascular smooth muscle cells senescence

## Abstract

Vascular smooth muscle cell (VSMC) senescence is a pivotal driver of atherosclerosis (AS), but molecular links to ageing‐related dysfunction are unclear. It is aimed to identify regulators of VSMC senescence and develop clinical interventions for ageing‐related AS. Using single‐cell RNA sequencing of human atherosclerotic carotid arteries and immunofluorescence validation, activating transcription factor 3 (ATF3) is identified as central to VSMC senescence. Mechanistic studies employ SMC‐specific ATF3 knockout mice, CUT&Tag‐seq, RNA/protein interaction assays, and m6A epitranscriptomic analyses. To bridge discovery to therapy, high‐throughput virtual screening is performed for ATF3‐targeting compounds and functionally validated hits. ATF3 deficiency in VSMCs accelerates ageing‐induced AS by promoting senescence. Multi‐omics showed ATF3 activates ATG7, triggering autophagy, while cytoplasmic ATG7 enhances ATF3 nuclear translocation, establishing a positive feedback loop. Ageing increases m6A methylation and decreases the stability of *Atf3* mRNA. Terazosin (TZ) diminishes the interaction between YTH N6‐methyladenosine RNA binding protein F2 (YTHDF2) and *Atf3* mRNA, helping to preserve *Atf3* mRNA stability. TZ is a promising therapeutic strategy for delaying VSMC senescence and preventing AS. ATF3 protects against VSMC senescence and AS by orchestrating autophagy via a novel ATF3‐ATG7 amplification loop. Repurposing TZ to stabilize ATF3 offers a translatable approach to combat ageing‐driven cardiovascular disease.

## Graphical abstract

Targeting ATF3 with terazosin to against VSMC Senescence and atherosclerosis. TZ reduces the binding affinity of YTHDF2 to *Atf3* mRNA, while enhancing mRNA stability. ATF3 promotes *Atg7* transcription, enhancing autophagy. ATG7 binds to ATF3 in the cytoplasm, facilitating ATF3 translocation to the nucleus and establishing positive feedback. By modulating ATF3, VSMCs' senescence can be improved, which in turn inhibits atherosclerosis.

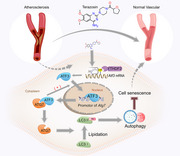

## 1 Introduction

Atherosclerosis (AS) is an age‐associated vascular disorder.^[^ ^1^, ^2^ ^]^ Vascular smooth muscle cells (VSMCs) constitute a major cellular component of atherosclerotic plaques and are characterized by senescence and increased apoptosis.^[^ ^3^ ^]^ VSMC senescence accelerates VSMC phenotypic switching,^[^ ^4^ ^]^ resulting in pathological structural deterioration of vessels and increased vascular stiffness.^[^ ^5^, ^6^, ^7^ ^]^ Hence, protecting VSMCs from senescence has emerged as a promising therapeutic target for treating AS. However, hardly any pharmacological intervention has been proven to be safe and effective for reversing VSMC senescence and AS in clinical practice. Therefore, pharmacological treatment strategies for reversing VSMC senescence and AS are needed clinically.

The Activator Protein 1/Activating Transcription Factor (AP1/ATF) family, which includes key members such as Proto‐oncogene c‐Fos, Proto‐oncogene c‐Jun, and ATF1 to ATF6, has been extensively studied for its critical roles in cardiovascular diseases.^[^ ^8^ ^]^ Our analysis of single‐cell RNA sequencing (scRNA‐seq) data from AS patients indicates that ATF3 may be a potential regulatory molecule for AS. ATF3 participates in extensive stress responses, and its proper activity is essential for sustaining normal physiological functions in cells.^[^ ^9^ ^]^ However, the role of ATF3 in senescence across different cell types and disease contexts is controversial. In certain models, ATF3 promotes senescence by activating senescence‐associated endogenous retroviruses and inducing an antiviral defense response.^[^ ^10^ ^]^ In fibroblasts, ATF3 drives senescence‐associated gene expression by remodeling chromatin accessibility.^[^ ^11^ ^]^ Furthermore, ATF3 was upregulated in the VSMCs of murine aortic aneurysm and dissection models, where it suppressed apoptosis by inhibiting the DNA damage response.^[^ ^12^ ^]^ Further studies are essential to unravel its context‐dependent mechanisms in age‐related pathologies.

Autophagy, a vital physiological process, promotes efficient cellular recycling by transporting dysfunctional cellular components to lysosomes for degradation and metabolic processing, supporting cell survival.^[^ ^13^ ^]^ The primary type of autophagy is autophagosome (AP) formation, which is facilitated by microtubule‐associated protein 1 light chain 3 (MAP1LC3/LC3).^[^ ^14^ ^]^ Autophagy‐related proteins such as autophagy‐related 3 and autophagy‐related 7 (ATG7) facilitate this process.^[^ ^15^, ^16^ ^]^ Reduced autophagy is associated with cardiovascular functional decline and increased susceptibility to cardiovascular diseases in older adults.^[^ ^17^ ^]^ Growing evidence suggests that enhancing autophagy can mitigate vascular ageing.^[^ ^18^, ^19^ ^]^ Enhancing autophagy to slow down vascular ageing is an emerging novel research field.

Terazosin (TZ), a widely used medicine in clinical practice, reduces smooth muscle tension and is utilized for managing benign prostatic hyperplasia and hypertension. Recent studies have revealed that TZ has anti‐apoptotic properties independent of its ability to block α1‐adrenoceptors.^[^ ^20^ ^]^ TZ has also been shown to reduce the risk of age‐related neurodegenerative diseases. TZ has therapeutic potential for Parkinson's disease^[^ ^21^ ^]^ and reduces amyloid‐beta production in Alzheimer's disease by inhibiting glycogen synthase kinase 3 beta.^[^ ^22^, ^23^ ^]^ We found that the novel mechanism of TZ seems to involve altering ATF3 levels to exert anti‐ageing effects.

This study aimed to explore the anti‐aging and anti‐AS effects of ATF3, as well as the underlying mechanisms.

## 2 Results

### 2.1 AS is Associated with Decreased ATF3 Expression and VSMC Senescence

**Figure 1:**
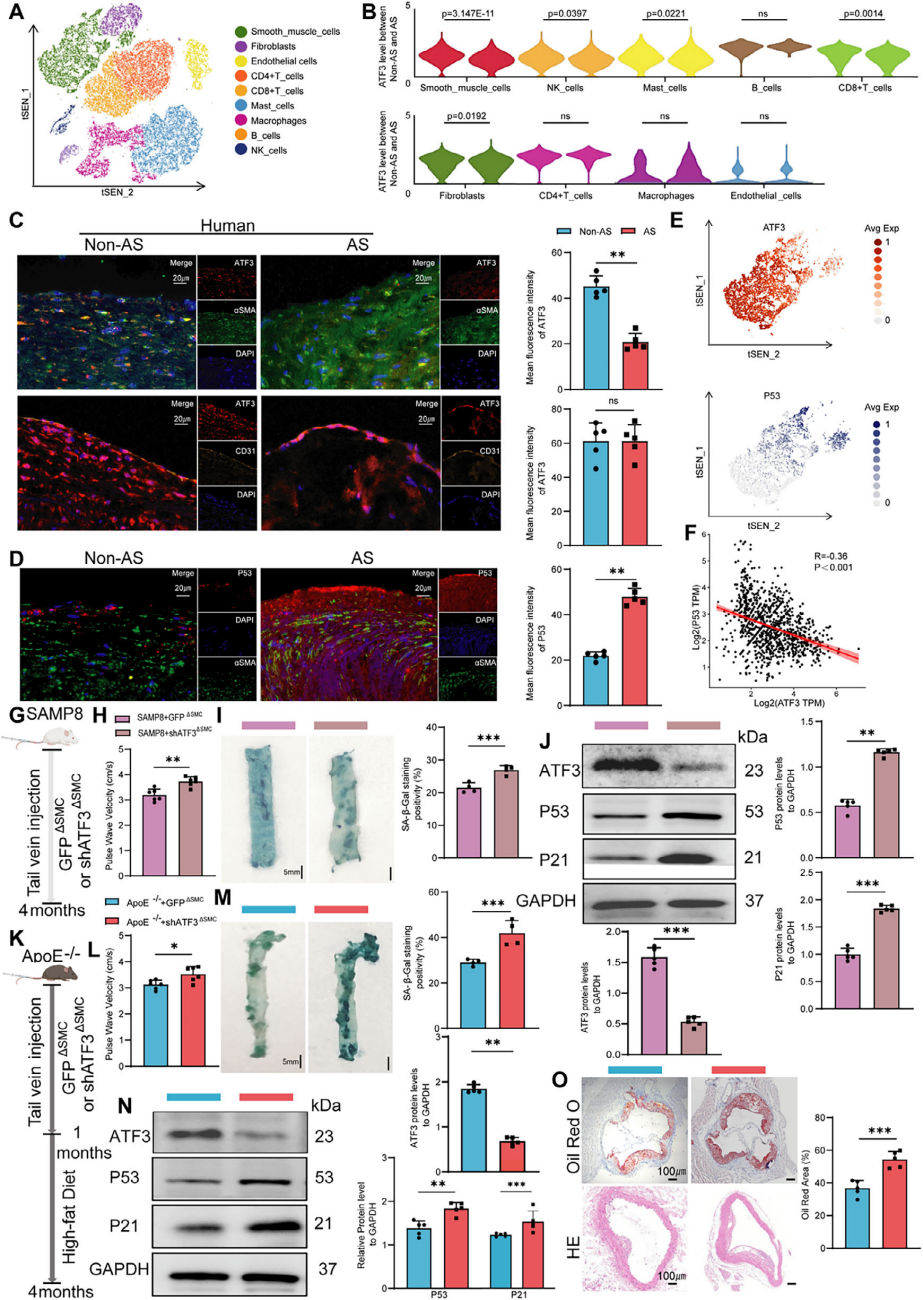
Vascular smooth muscle cells (VSMCs) in atherosclerosis (AS) simultaneously display signs of senescence and a decline in ATF3 content. A) t‐SNE analysis of single cells from the carotid arteries of atherosclerosis patients, with 9 major cell types labeled in different colors. B) The violin plot illustrating the variation in levels of ATF3 across different cell types. C) IF imaging of ATF3 (red), α‐SMA (green), CD31(orange), and nuclei (blue) in human carotid arteries (scale bars = 20 µm; *n *= 5). D) IF imaging of p53 (red), α‐SMA (green), and nuclei (blue) in human carotid arteries (scale bars = 20 µm; *n *= 5). E) Feature plot illustrating the expression distribution of ATF3 and p53 in VSMCs. F) Scatter plot illustrating the correlation between p53 and ATF3 expression levels. G) Experimental design: SMC‐specific knockdown of ATF3 in SAMP8 mice (*n *= 6). H) Pulse wave velocity (PWV) in SAMP8 mice (*n *= 6). I) SA‐β‐gal staining of mouse aortas (scale bars = 5 mm, *n *= 4). J) Western blot analysis of ATF3, p53, and p21 expression in SAMP8 mice aortas (*n *= 5). K) Experimental design: SMC‐specific knockdown of ATF3 in ApoE^‐/‐^ mice. After 4 weeks, mice fed with high‐fat diet for 3 months (*n *= 8). L) PWV in ApoE^‐/‐^ mice (*n *= 6). M) SA‐β‐gal staining of mouse aortas (scale bars = 5 mm, *n* = 4). N) Western blot analysis of ATF3, p53, and p21 expression in ApoE^‐/‐^ mice aortas (*n *= 5). O) Oil Red O staining and Hematoxylin and eosin staining of mouse aortas (scale bars = 100 µm, *n *= 5). Error bars represent the mean ± standard deviation. The unpaired *t*‐test was used to compare data; *p < 0.05, **p < 0.01, ***p < 0.001.

To identify potential AS‐related candidate genes within the AP1/ATF transcription factor family, single‐cell RNA sequencing analysis was conducted on twelve human carotid artery samples from the public GEO database (GSE253903), including six cases with carotid atherosclerotic plaques. Based on the marker genes of each cluster, cells from carotid atherosclerotic plaques were primarily classified into nine cell types (**Figure** **1**A; Figure S1C, Supporting Information). Among the various cell types, the AP1 family exhibited the greatest fold change in ATF3 expression, especially in VSMCs (Figure 1B; Figure S1D, Supporting Information).

Carotid atherosclerotic samples from patients with AS were collected. VSMCs were identified through immunofluorescence (IF) staining using the SMC‐specific marker alpha‐smooth muscle actin (α‐SMA). Endothelial cells (ECs) were labeled with platelet endothelial cell adhesion molecule‐1 (CD31).^[^ ^24^ ^]^ IF analysis further revealed that ATF3 expression was downregulated in VSMCs but not in ECs (Figure 1C). On the other hand, VSMCs within atherosclerotic plaque areas exhibited pronounced senescence, as evidenced by upregulated expression of the senescence markers p53 and p21 (Figure 1D; Figure S1E, Supporting Information). VSMC phenotypic modulation drives the progression of AS^[^ ^25^ ^]^ and represents a key hallmark of VSMC senescence.^[^ ^26^ ^]^ The phenotypic plasticity of VSMCs drives alterations in the architecture and physiology of the vessel wall, contributing to the progression of vascular stiffening.^[^ ^27^ ^]^ The IF assay revealed downregulation of a contractile phenotypic marker, smooth muscle protein 22α (SM22α), and upregulation of the synthetic marker osteopontin (OPN) compared with those in healthy controls (Figure S1E, Supporting Information).

To further confirm the above findings, we collected aortic specimens from high‐fat diet (HFD)‐fed ApoE^‐/‐^ mice and found that the decrease in ATF3 was specific to VSMCs (Figure S2A, Supporting Information) and that VSMCs in atherosclerotic plaques of the aorta underwent senescence and phenotypic transformation compared with those in the control group (Figure S2B, Supporting Information). We identified in age‐stratified mouse cohorts that vascular senescence progressively intensifies with age, alongside elevated expression of p21 and p53 proteins and a concomitant decline in ATF3 mRNA/protein levels (Figure S2C,D, Supporting Information). We were interested in the association between ATF3 and VSMC senescence, and we observed that the expression distributions of ATF3 and p53 in the VSMCs trended in opposite directions (Figure 1E). Additionally, a negative correlation was observed between ATF3 and p53 levels (Figure 1F). Therefore, ATF3 might be an important molecule involved in the regulation of VSMC senescence and AS.

### 2.2 ATF3 Deficiency in VSMCs Exacerbates VSMC Senescence and AS

To evaluate whether ATF3 deficiency contributes to VSMC senescence, we delivered shRNA‐ATF3 via recombinant adeno‐associated virus serotype 9 vectors using the SM22α promoter (specific to SMCs) to knock down ATF3 expression (Figures S2E,F,S3A, Supporting Information). Senescence‐accelerated mouse prone 8 (SAMP8) mice were selected as a model to study age‐related changes in the vasculature (Figure 1G), and they displayed a disruption in vascular homeostasis and a markedly elevated incidence of atherosclerotic development compared with senescence‐accelerated mouse resistance 1 (SAMR1) mice starting at 10–14 months.^[^ ^28^ ^]^ Pulse wave velocity (PWV) was used to assess vascular stiffness, as increased stiffness reflects aggravated VSMC senescence and vascular ageing.^[^ ^29^ ^]^ Compared with SAMP8+GFP^∆SMC^ mice, SAMP8+shATF3^∆SMC^ mice significantly increased vascular stiffness (Figure 1H). Knockdown of ATF3 exacerbated VSMC senescence, as evidenced by an increased SA‐β‐gal‐positive staining area and elevated levels of senescence markers (Figure 1I,J). To illustrate the effect of ATF3 on ageing‐induced AS, first, a SAMP8 mouse model (vs SAMR1 controls) was used to explicitly evaluate the interplay between vascular ageing and AS susceptibility. Compared to age‐matched SAMR1 mice, SAMP8 mice exhibited no significant atherosclerotic features prior to 3 months of age (Figure S3B, Supporting Information). However, with increasing age, distinct plaque formation was detected through Oil Red O staining of the aortic roots of 14‐month‐old SAMP8 mice (Figure S3B, Supporting Information). In 14‐month‐old SAMP8 mice, smooth muscle cells exhibited a significantly augmented inflammatory response (Figure S3C, Supporting Information), as demonstrated by elevated expression of cluster of vascular cell adhesion molecule‐1, interleukin‐6, and tumor necrosis factor‐alpha. These molecular alterations represent a critical hallmark of the phenotypic transition from normal smooth muscle characteristics to an AS‐prone state.^[^ ^30^ ^]^ In line with existing research, the premature vascular ageing phenotype inherent in SAMP8 mice has been shown to be a predisposing factor for AS pathogenesis.^[^ ^31^ ^]^ ATF3 knockdown exacerbated AS progression in 14‐month‐old SAMP8 mice, as evidenced by increased plaque area compared with that of normal control littermates (Figure S3B,C, Supporting Information).

Next, we explored whether ATF3 deficiency exacerbates AS by promoting VSMC senescence in ApoE^‐/‐^ mice. The experiment included two groups, ApoE^‐/‐^+GFP^∆SMC^ mice and ApoE^‐/‐^+shATF3^∆SMC^ mice, which were fed a HFD for 3 months (Figure 1K). We assessed PWV and plaque formation using SA‐β‐gal, hematoxylin‐eosin (HE) staining, Elastica van Gieson (EVG) staining, transmission electron microscope (TEM), and lipid deposition. As anticipated, ApoE^‐/‐^+shATF3^∆SMC^ mice exhibited increased vascular stiffness (Figure 1L) and VSMC senescence (Figure 1M,N). The absence of ATF3 promoted the degradation of elastic fibers (Figure S3D, Supporting Information). Western blotting (WB) and Immunofluorescence (IF) results demonstrated that ATF3 knockdown in VSMCs significantly increased OPN levels and reduced SM22α expression, suggesting enhanced VSMC phenotypic switching (Figure S3D–F, Supporting Information). Furthermore, ApoE^‐/‐^+shATF3^∆SMC^ mice exhibited lipid accumulation at the aortic valve and increased plaque development (Figure 1O).

### 2.3 ATF3 Promotes Autophagy by Increasing *Atg7* Transcription

**Figure 2:**
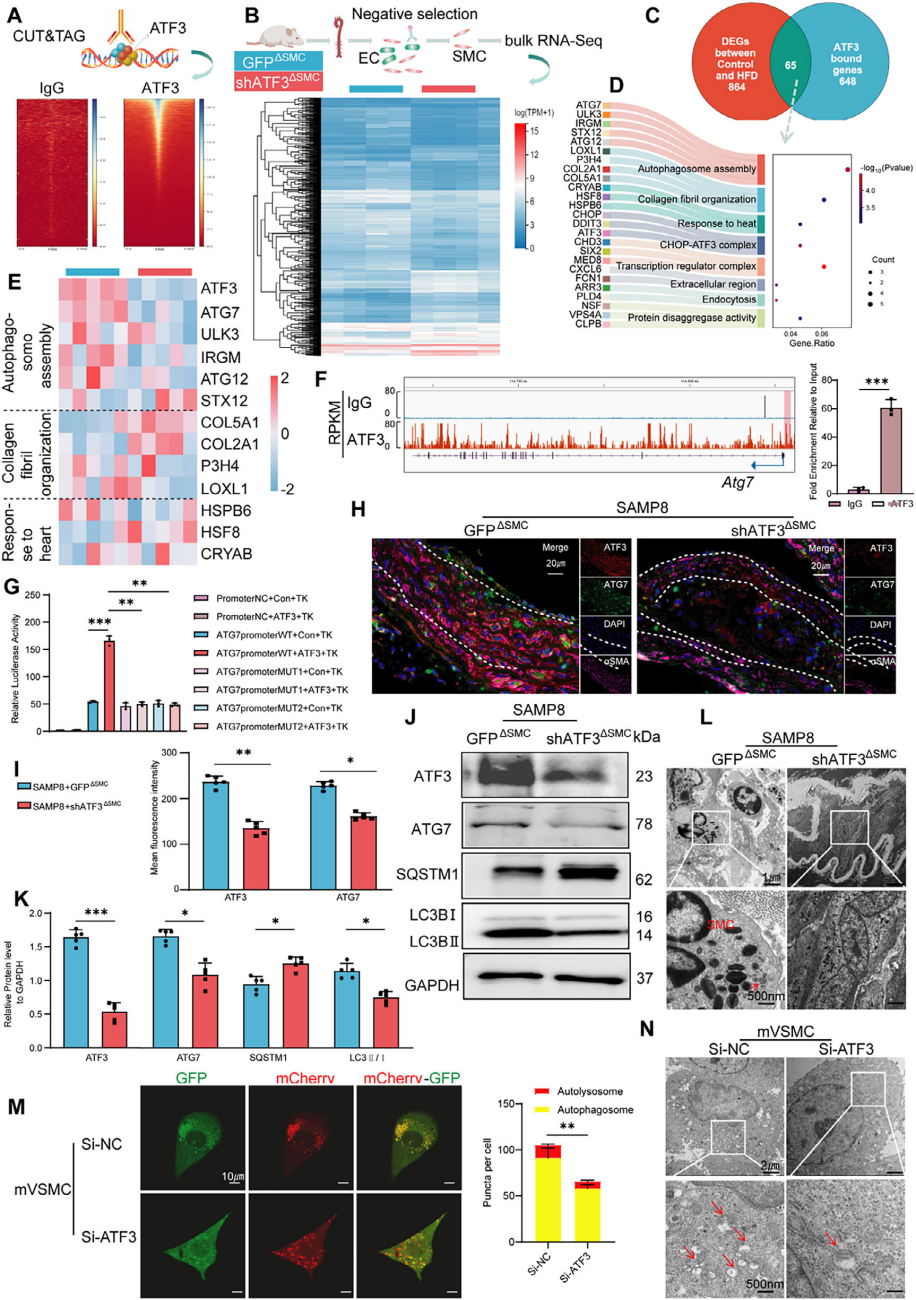
ATF3 facilitates autophagy through the upregulation of *Atg7* transcription. A) The CUT&Tag calling peaks are enriched in the transcription start site (TSS) area by the heatmap. B) Clustering heatmap of differentially expressed genes between SAMP8+GFP^∆SMC^ and SAMP8+shATF3^∆SMC^ mice. C) Veen diagram of CUT&Tag and RNA‐seq intersecting targets. D) Dot plot of the GO pathway enrichment analysis of intersecting targets. E) Variations in the expression of genes enriched in the top three signaling pathways. F) ATF3 exhibits a significant binding peak in the promoter region of the *Atg7* gene in CUT&Tag. And ChIP‐qPCR of ATF3 binding to the *Atg7* promoter region. G) Luciferase assay of PGL3‐basic vector carrying wild‐type or mutant *Atg7* 3′‐UTR co‐transfected with ATF3 or negative control in HEK‐293 T cells (*n *= 3). TK: pRL‐TK, Renilla Luciferase and Thymidine Kinase promoter. H,I) Immunofluorescent staining of ATF3 (red), ATG7 (green), α‐SMA (pink), and nuclei (blue) in mouse aortas (scale bars = 20 µm; *n *= 5). J,K) Western blot analysis of ATF3, LC3B‐II/LC3B‐I, SQSTM1 and ATG7 expression in mouse aortas (*n *= 5). L) TEM observation of autophagic phenomena in VSMCs in mouse aortas (*n *= 5), arrows indicate autophagosomes or autolysosomes (scale bars, 1 µm and 500 nm). M) Fluorescence analysis of VSMCs transfected with the mCherry‐GFP‐LC3 reporter (*n *= 3); red: autophagosomes, yellow: autolysosomes (scale bars, 10 µm). N) TEM observation of autophagic phenomena in VSMCs (*n* = 3), arrows indicate autophagosomes or autolysosomes (scale bars, 2 µm and 500 nm). Error bars represent the mean ± standard deviation. The unpaired *t*‐test (I,K), one‐way ANOVA (G), and Mann–Whitney *U*‐test (M) were used to compare data; *p < 0.05, **p < 0.01, ***p < 0.001.

**Figure 3:**
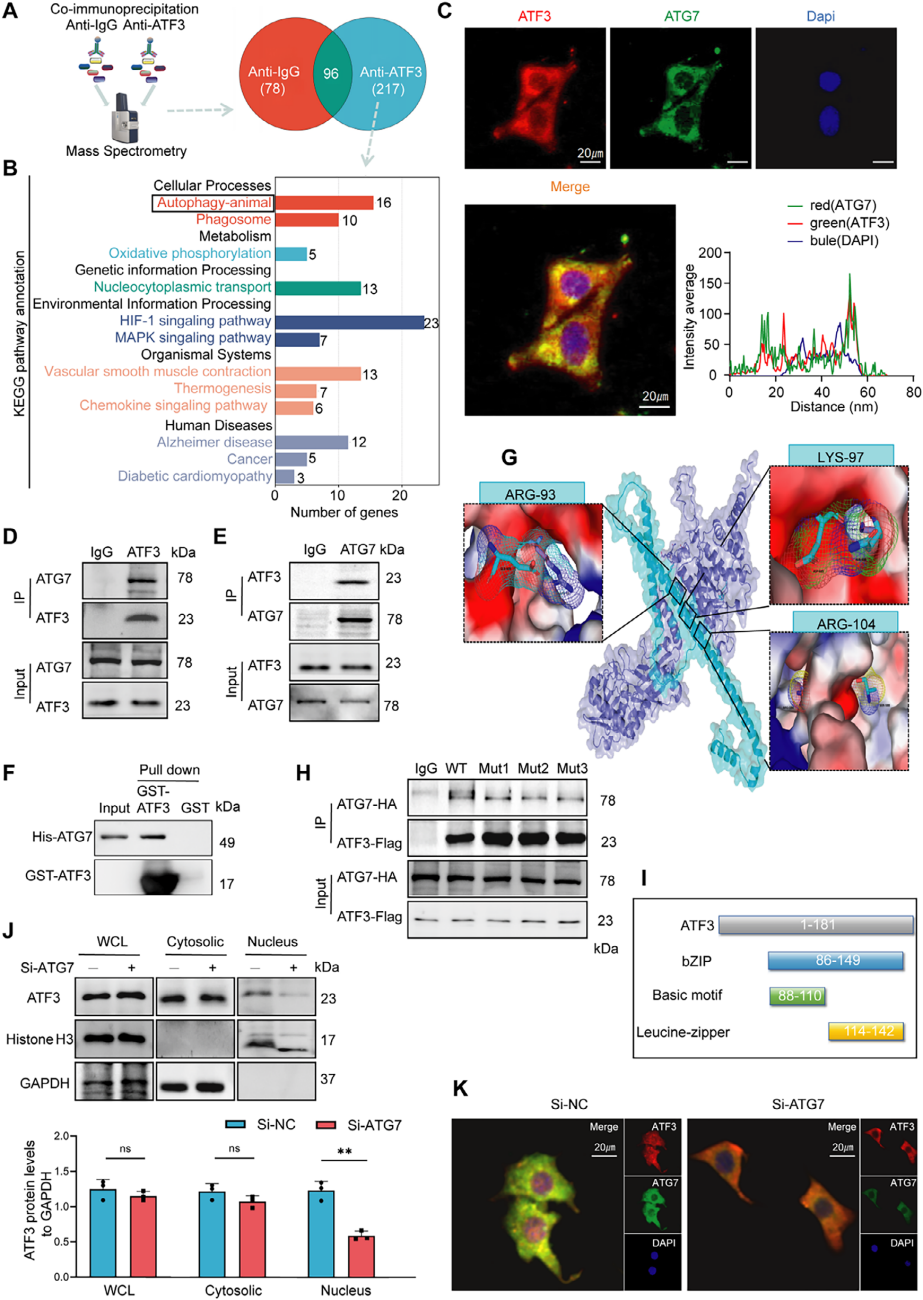
ATG7 promotes the translocation of ATF3 into the nucleus. A,B) Pull‐down proteomics analysis of ATF3 and IgG antibodies: differential protein Venn diagram (A) and KEGG pathway classification analysis (B). C) Fluorescence analysis of the cellular distribution of ATF3 and ATG7 (scale bars = 100 µm). D) Immunoprecipitation of ATG7 from VSMCs using ATF3 and IgG antibodies, respectively. E) Immunoprecipitation of ATF3 from VSMCs using ATG7 and IgG antibodies, respectively. F) GST‐pull‐down of His‐ATG7 from HEK‐293T cells with a GST‐ATF3 fusion protein. G) Visualization of the interaction between ATF3 and ATG7; blue: ATF3, purple: ATG7. The spatial structure is presented through a cartoon, and the surface is presented as a combination pocket. H) Different protein complex components from WT, Mutant1‐ (ATF3–A93G), Mutant2‐ (ATF3–L97G), or Mutant3‐ (ATF3–A104G) transfected HEK‐293T cells immunoprecipitated using the Flag tag antibody. I) 2D structure diagram of ATF3. J) Western blot analysis of ATF3 expression in the nucleus and cytoplasm following transfection of VSMCs with Si‐NC or Si‐ATG7 (*n *= 3). K) Fluorescence analysis of ATF3 distribution in the nucleus and cytoplasm (scale bars, 20 µm; *n *= 3). Input: whole cell lysates, IP: immunoprecipitants, Mut: Mutant. Error bars represent the mean ± standard deviation. The Mann–Whitney *U*‐test was performed to compare data; **p < 0.01.

To investigate how ATF3, as a transcription factor, exerts its transcriptional regulatory functions, CUT&Tag and RNA‐seq were performed to map the genome‐wide DNA‐binding sites and likely transcriptional targets of ATF3. According to the CUT&Tag identification, compared with the control, ATF3 displayed binding peaks around the transcription start site, 2 kb away (**Figure** **2**A). As shown in Figure 2B, after negative selection of VSMCs in the vasculature was performed, RNA‐seq analysis was conducted, and the heatmap displays the differentially expressed genes (DEGs) between the SAMP8+GFP^∆SMC^ and SAMP8+shATF3^∆SMC^ mice. Cross‐analysis of the CUT&Tag and RNA‐seq data led to the identification of 65 candidate transcriptional targets directly regulated by ATF3 (Figure 2C). These genes were primarily enriched in signaling pathways related to autophagy and collagen synthesis (Figure 2D). Autophagy is a pivotal juncture for cellular processes that are disrupted in age‐related diseases.^[^ ^32^ ^]^ real‐time quantitative PCR (qPCR) experiments revealed that *Atg7* was significantly downregulated after ATF3 knockdown in SAMP8 mice (Figure 2E). The CUT&Tag results revealed significant binding peaks of ATF3 in the promoter region of *Atg7* (Figure 2F). Specific primers targeting the *Atg7* promoter region were designed for Chromatin Immunoprecipitation (ChIP)‐qPCR, and the results revealed significant enrichment of ATF3 binding signals in the *Atg7* promoter region (Figure 2F). We propose that *Atg7* could be a potential target gene of ATF3. The JASPAR database was used to predict the binding region of ATF3 to the *Atg7* promoter. *Atg7* promoter mutations 1 (−32 to −25 bp) and 2 (−1538 to −1531 bp) were designed, and a dual‐luciferase reporter assay was conducted for further validation in HEK‐293T cells. The results revealed that ATF3 significantly increased luciferase activity in the wild‐type *Atg7* 3’‐untranslated region (3'UTR) reporter, whereas ATF3 did not affect the luciferase activity of *Atg7* promoter mutations (Figure 2G). These findings suggest direct binding of ATF3 to the *Atg7* promoter region. scRNA‐seq of AS lesions demonstrated co‐expression patterns between ATF3 and ATG7 in VSMCs (Figure S4A, Supporting Information), which was supported by a statistically significant positive correlation (Figure S4B, Supporting Information). IF labeling demonstrated the concurrent downregulation of ATG7 in response to ATF3 knockdown in SAMP8 mice (Figure 2H,I). ATG7 acts as an E1‐like enzyme, facilitating the conjugation of LC3 to phosphatidylethanolamine.^[^ ^33^ ^]^ As expected, the decrease in ATG7 levels coincided with a reduction in autophagy (Figure 2J,K). In SAMP8+shATF3^∆SMC^ mice, the LC3B‐II/LC3B‐I ratio decreased resulting in an increase in the level of the autophagy substrate sequestosome1 (SQSTM1).^[^ ^34^ ^]^ Fewer APs were observed in the VSMCs with ATF3 deficiency via TEM (**Figure** **3**L).

Small‐interfering RNA (siRNA) was used to investigate the effects of ATF3 in vitro (Figure S4C–F, Supporting Information), ATF3 knockdown led to a decrease in ATG7 at both the mRNA and protein levels (Figure S4G–I, Supporting Information). Bafilomycin A1 (BafA1) prevents the autophagosome‐lysosome fusion and subsequently results in the accumulation of LC3‐II.^[^ ^35^ ^]^ A lake of ATF3 further decreased the LC3B‐II/LC3B‐I ratio in the presence of BafA1 in mVSMCs (Figure S4J,K, Supporting Information). mVSMCs were infected with Adenovirus (AdV) carrying GFP‐mCherry‐LC3 fusion protein. GFP signal quenching occurred in the highly acidic environment of lysosomes but not by mCherry (red fluorescent protein). We found that the numbers of mCherry‐single positive (red dot) and mCherry‐GFP double positive puncta (yellow dot) were decreased in mVSMCs with ATF3 deficiency (Figure 2M). Loss of ATF3 decreased the level of APs and autolysosomes in mVSMCs by TEM (Figure 2N). These findings suggest that ATF3 facilitates autophagy through the stimulation of ATG7 transcription.

### 2.4 ATG7 Promotes the Nuclear Entry of ATF3

Co‐immunoprecipitation (Co‐IP) assay and mass spectrometry analysis were used to identify the proteins binding to ATF3. Proteomic analysis revealed that 217 proteins were pulled down by ATF3 (Figure 3A). Notably, KEGG enrichment analysis highlighted significant enrichment of the autophagy pathway (Figure 3B). There were 16 proteins enriched in the autophagy pathway. Among these, ATG7 was highly ranked in both intensity‐based absolute quantification and confidence (Q‐score) within the autophagy pathway (Table S2, Supporting Information). IF co‐localization analysis revealed significant co‐localization of ATF3 and ATG7 in the cytoplasm (Figure 3C). Interactions between ATF3 and ATG7 were detected using Co‐IP and WB. The ATF3‐specific antibody immunoprecipitated endogenous ATG7 from VSMC lysates (Figure 3D). Conversely, the ATG7 antibody also immunoprecipitated endogenous ATF3 (Figure 3E). The results of the Glutathione S‐transferase (GST) pull‐down assay confirmed direct binding between ATF3 and ATG7 (Figure 3F). ATF3 immobilized on the CM5 sensor chip can bind ATG7 with an affinity constant of 963 nm as determined via a surface plasmon resonance assay (Figure S4L, Supporting Information). These findings suggest that ATF3 directly binds to ATG7

The binding pattern of ATG7 to ATF3 was analyzed using the molecular docking method. The strongest binding complex was selected for visualization (Figure 3G), with a central energy of −542 kcal mol^−1^. To further clarify the actual binding site between ATG7 and ATF3, the amino acid binding site was predicted (Table S3, Supporting Information). The corresponding mutant ATF3 plasmids were constructed with three variants: ATF3‐A93G, ATF3‐L97G, and ATF3‐A104G. Compared with those of wild‐type ATF3, Co‐IP experiments demonstrated a reduced interaction of all ATF3 variants with ATG7 (Figure 3H), suggesting that a stable complex formed between the compounds ATG7 and ATF3 at amino acids 93, 97, and 104.

According to the forecasts, these three binding sites are located in the basic structural domain of the ATF3 (amino acids 88–110), which is responsible for the nuclear translocation of the ATF3 (Figure 3I). To confirm whether ATG7 acts as an auxiliary factor for ATF3, we knocked down ATG7 in mVSMCs (Figure S5A–D, Supporting Information) and assessed the nuclear translocation of ATF3. The nuclear‐to‐cytoplasmic ratio of ATF3 was significantly lower in the ATG7‐knockdown group than in the control group (Figure 3J). IF analysis further confirmed disrupted ATF3 concentrations in the nucleus after ATG7 knockdown, in contrast with the results of the control group (Figure 3K). Moreover, we demonstrated that ATG7 promotes ATF3 nuclear translocation without concomitant translocation into the nucleus. WB and IF analyses confirmed ATF3 enrichment in nuclei upon ATG7 overexpression in VSMCs, whereas ATG7 exhibited cytoplasmic retention without nuclear entry (Figure S5E–G, Supporting Information).

These results indicate that a positive feedback loop formed between ATF3 and ATG7. ATG7 bound to ATF3 in the cytoplasm, increasing the nuclear translocation of ATF3, while ATF3 bound to the promoter region of *Atg7* and increased its transcription in the nucleus, increasing the expression of ATG7 mRNA and protein and initiating the downstream autophagy process.

### 2.5 TZ Enhances *Atf3* mRNA Stability to Inhibit VSMC Senescence and Phenotype Switching

**Figure 4:**
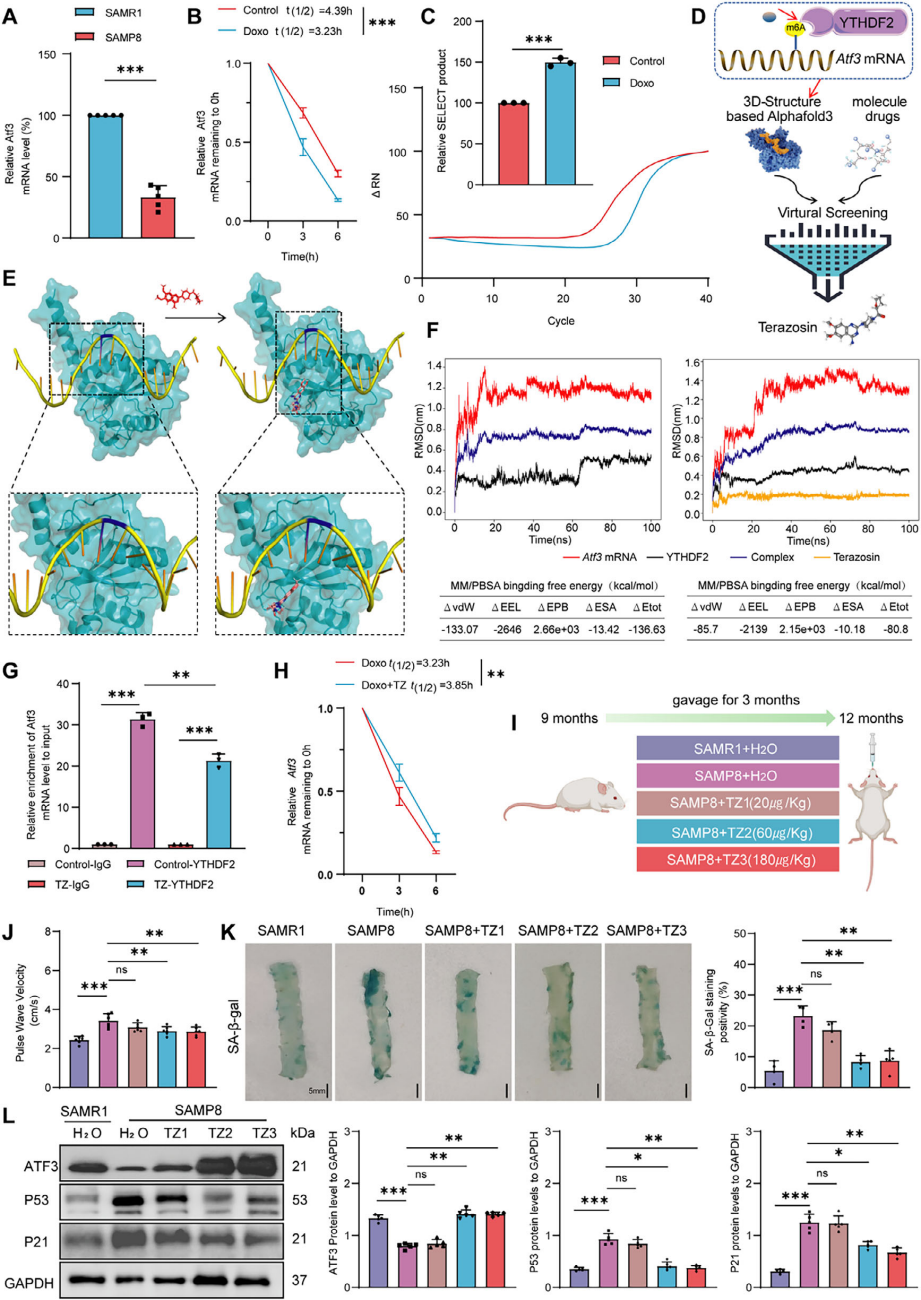
Terazosin (TZ) improves age‐related phenotypes in SAMP8 mice via ATF3. A) qPCR analysis of *Atf3* in SAMP8 mice carotid arteries (*n *= 5). B) Senescence decreases the half‐life of *Atf3* mRNA in VSMCs (*n *= 3). C) Relative m6A methylation abundances of *Atf3* mRNA at the 1627 site measured by the SELECT method in VSMCs (*n *= 3). D) Experimental design: screening small molecular drugs targeting ATF3. E) Visualization of the interaction between *Atf3* mRNA, YTHDF2, and TZ. blue: YTHDF2, yellow: *Atf3* mRNA with m6A modifications (blue), red: TZ. F) Structural backbone RMSD variations from molecular dynamics simulations and MM‐PBSA‐derived binding free energy values. G) Carry out YTHDF2‐RNA immunoprecipitation (RIP) in TZ‐treated VSMCs, then use qPCR to evaluate Atf3 mRNA expression. Analyze the influence of TZ on YTHDF2's affinity for Atf3 mRNA (*n* = 3). H) TZ increases the half‐life of *Atf3* mRNA in VSMCs (*n *= 3). I) Experimental design: Administered TZ via gavage to SAMP8 mice for three months, with SAMR1 as the control group (TZ1: 20 µg kg^−1^, TZ2: 60 µg kg^−1^, TZ3: 180 µg kg^−1^; *n* = 6). J) PWV in SAMR1 mice and SAMP8 mice (*n *= 6). K) SA‐β‐gal staining of mouse aortas (scale bars = 5 mm, *n *= 5). L) Western blot analysis of ATF3, p53, and p21 expression in mouse aortas (*n *= 5). Error bars represent mean ± standard deviation. The one‐way ANOVA (G, J, L) and unpaired *t*‐test (A–C) were used to compare data; *p < 0.05, **p < 0.01, ***p < 0.001.

We explored a clinical drug that specifically targets ATF3. ATF3 mRNA levels were lower in SAMP8 mice than in SAMR1 mice (**Figure** **4**A). We assessed the half‐life (*t_1/2_*) of *Atf3* mRNA in VSMCs using qPCR. Compared with that in the control group, the half‐life of *Atf3* mRNA in senescent VSMCs was shorter (Figure 4B). The most abundant internal modification in mRNAs is N6‐methyladenosine (m6A), which affects the stability and translation efficiency of mRNAs.^[^ ^36^ ^]^ We also investigated whether the decreased expression of ATF3 mRNA and protein arises from a m6A‐mediated epitranscriptomic mechanism. We analyzed publicly available m6A sequencing data using RMBase v2.0 and SRAMP.^[^ ^37^, ^38^ ^]^ For detection purposes, we selected the three methylation sites (777, 1434, and 1627) that received the highest scores in both prediction databases. Data from the single‐base elongation‐ and ligation‐based qPCR amplification (SELECT) assay revealed that the m6A level at adenosine residue 1627 in *Atf3* mRNA was increased in senescent VSMCs (Figure 4C). The m6A reader protein, YTH N6‐methyladenosine RNA binding protein F2 (YTHDF2) binds to m6A‐modified *Atf3* mRNA, triggering its decay.^[^ ^39^ ^]^ Thus, blocking the binding of YTHDF2 to *Atf3* mRNA could serve as a potential approach to target ATF3. We conducted a virtual screening of 16 000 000 compounds from the FDA‐approved drug database, traditional Chinese medicine (TCM) database, and PubChem database to identify therapeutic agents that attenuate the binding between *Atf3* mRNA and YTHDF2 (Figure 4D). The top 10 molecules with the highest affinity are listed in Table S4 (Supporting Information). The stability of the complex was assessed using molecular dynamics (MD) simulations. Ultimately, the compound Terazosin (TZ), which had the highest binding affinity, was prioritized for further mechanistic validation. The binding modes of TZ, *Atf3* mRNA, and YTHDF2 are depicted in Figure 4E. The root‐mean‐square deviation (RMSD), radius of gyration (Rg), and root mean square fluctuation (RMSF) were monitored throughout the 100‐ns MD simulations to assess the structural stability of the systems (Figure 4F; Figure S6A,B, Supporting Information). As a result of TZ addition, *Atf3* mRNA exhibited enhanced structural fluctuations and a destabilized binding interface with YTHDF2. The increase in total energy (ΔEtot) upon TZ addition further supports the weakened binding affinity between *Atf3* mRNA and YTHDF2 (Figure 4F; Figure S6C,D, Supporting Information). An RNA immunoprecipitation (RIP) assay was then performed to assess the binding of YTHDF2 with *Atf3* mRNA. Notably, the presence of TZ significantly diminished the affinity of YTHDF2 for m6A‐modified *Atf3* mRNA (Figure 4G). TZ extended the *Atf3* mRNA half‐life in ageing VSMCs (Figure 4H).

We further investigated the potential regulatory effects of TZ on ATF3 in ECs. In contrast to that in VSMCs, ATF3 mRNA in ECs was inherently highly stable and was not further extended by TZ (Figure S7A,B, Supporting Information). A plausible explanation is that m6A modification of ATF3 mRNA in ECs remains unaltered during ageing (Figure S7C, Supporting Information).

To exclude the possibility that TZ may target other members of the ATF family, we conducted RNA‐seq on TZ‐treated VSMCs from SAMP8 mice. Enrichment analysis of the DEGs revealed that the ATF3‐CHOP signaling axis was the most prominently altered pathway (Figure S7D, Supporting Information). Notably, qPCR validation demonstrated no significant alterations in the mRNA levels of other ATF family members, including ATF2 and ATF4, compared with those in vehicle controls (Figure S7E, Supporting Information).

Further validation of the therapeutic effects of TZ was carried out through in vivo experiments. SAMP8 and SAMR1 mice were randomly divided into five groups and treated with three different TZ doses (Figure 4I). After 3 months of treatment, the basic characteristics of the mice were recorded (Table S5, Supporting Information). No significant differences were observed among the five groups in terms of body weight, serum glucose level, or blood lipid level at multiple time points during drug administration.

Additionally, HE staining of liver and kidney histology revealed no apparent structural disarrangement, indicating rare hepatotoxicity and renal toxicity (Figure S8A, Supporting Information). Mice treated with TZ showed a decrease in blood pressure (BP), but the difference was not statistically significant (Figure S8B,C, Supporting Information).

The PWV of SAMP8 mice was greater than that of SAMR1 mice. TZ treatment decreased vascular stiffness in SAMP8 mice (Figure 4J). The senescence markers SA‐β‐gal activity, p53, and p21 were assessed. TZ treatment not only increased ATF3 mRNA and protein levels but also decreased SA‐β‐gal activity, p53, and p21 mRNA and protein levels in SAMP8 mice (Figure 4K,L; Figure S8D–F, Supporting Information). The vessels of prematurely aged SAMP8 mice expressed more synthetic phenotypic markers, while those of TZ‐treated SAMP8 mice showed more contractile phenotypic markers (Figure S8G–L; Figure S9A, Supporting Information). TZ treatment reduced the broken ends of elastic fibers and restored the arrangement of collagen fibers, as shown by HE and EVG staining and TEM analysis (Figure S9B, Supporting Information). These findings suggest that TZ slows the vascular ageing process in SAMP8 mice. The lowest effective dose that reduced vascular stiffness (60 µg Kg^−1^) was selected as the therapeutic dose for further experimental exploration based on the above results.

**Figure 5:**
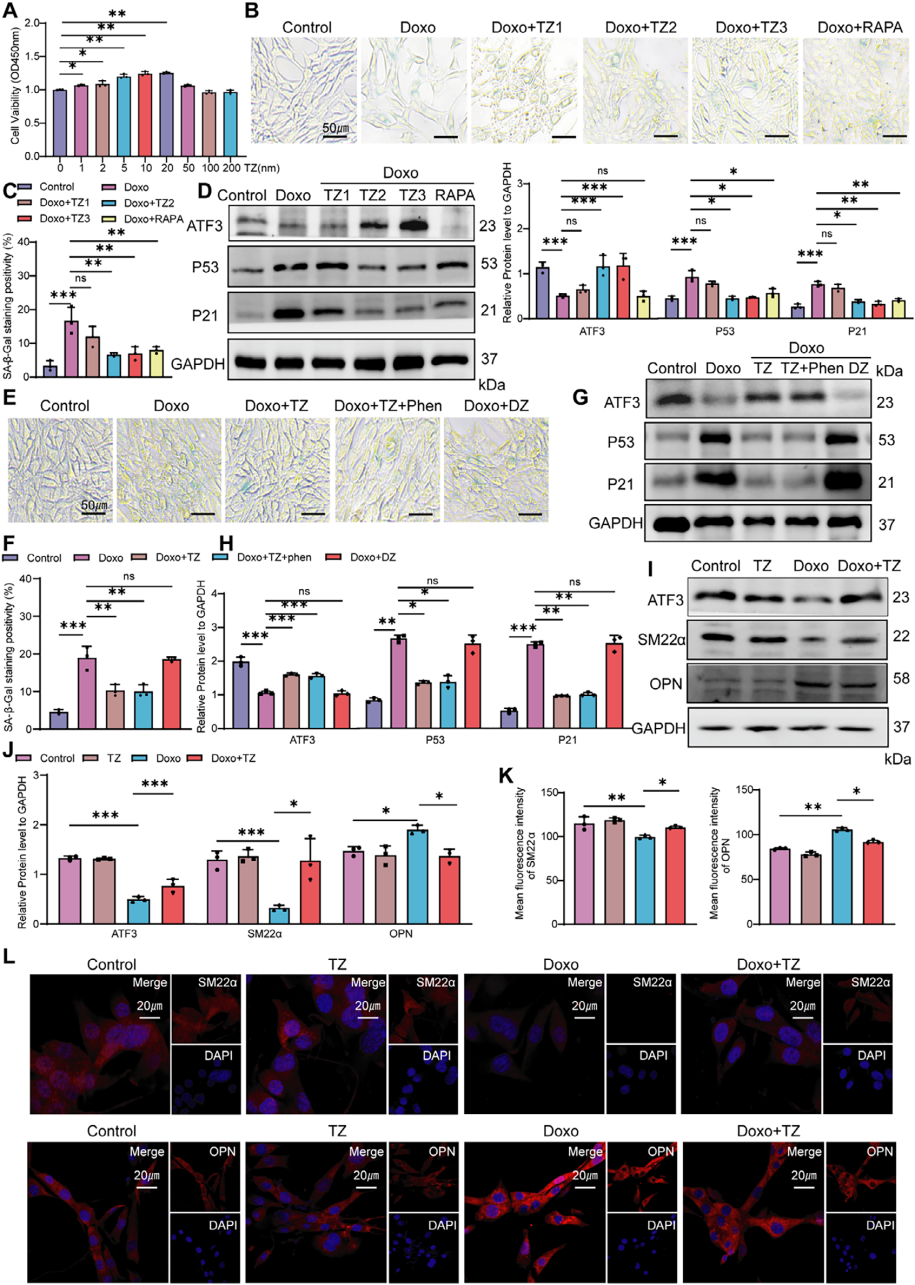
TZ reverses VSMC senescence and phenotype conversion. A) Analysis of cell viability in VSMCs treated with varying TZ concentrations for 48 h (*n *= 3). B,C) SA‐β‐gal staining of VSMCs (scale bars = 50 µm; *n *= 3). D) Western blot analysis of ATF3, p53, and p21 expression in VSMCs (*n *= 3). E,F) Prior to adding TZ to the senescent VSMCs, pre‐treat with Phen (phenoxybenzamine) for 4 h, using DZ (Doxazosin) as a negative control. E) SA‐β‐gal staining of VSMCs (scale bars = 50 µm; *n *= 3). F) Western blot analysis of ATF3, p53, and p21 expression in VSMCs (*n *= 3). G–I) Western blot analysis of SM22α and OPN expression in VSMCs (*n *= 3). J–L) Immunofluorescent staining of SM22α (red), OPN (red), and nuclei (blue) in VSMCs (scale bars = 20 µm; *n *= 3). Error bars represent mean ± deviation. The Kruskal–Wallis test was performed for data comparison. *p < 0.05, **p < 0.01, ***p < 0.001.

Mouse primary VSMCs were utilized to illustrate the protective effect of TZ in vitro. Cell viability assays showed enhanced cell activity at concentrations ranging 1–20 nm (**Figure** **5**A). Consequently, the concentration ranges of TZ that do not decrease cell viability were categorized into three groups: TZ1 (1 nm), TZ2 (10 nm), and TZ3 (20 nm). Doxorubicin was used to induce VSMC senescence, as previously reported.^[^ ^40^, ^41^ ^]^ Rapamycin (RAPA), known for its ability to inhibit cell senescence, was selected as a positive control in this study.^[^ ^42^, ^43^ ^]^ The anti‐ageing effects of TZ were assessed, and a significant reduction in SA‐β‐gal activity was observed after TZ administration (Figure 5B,C). Similar to RAPA, TZ2 and TZ3 effectively reduced the degree of senescence in VSMCs (Figure 5D), leading to the use of a 10 nm TZ concentration as the experimental therapeutic dose for further validation. In addition, we investigated whether the anti‐ageing effect of TZ depends on α1 blockade. In experiments including SA‐β‐gal and WB, the anti‐ageing effect of TZ was not altered by prior exposure to the irreversible α‐blocker phenoxybenzamine (phen). Doxazosin (DZ) failed to protect VSMCs from senescence, indicating that TZ does not exert its anti‐ageing effects by acting as an α1‐adrenoceptor blocker (Figure 5E–H). WB (Figure 5I,J) and IF assays (Figure 5K,L) subsequently revealed that TZ inhibited the phenotypic transformation of VSMCs.

### 2.6 Silencing ATF3 in VSMCs Abolishes the Anti‐Ageing Effects of TZ

**Figure 6:**
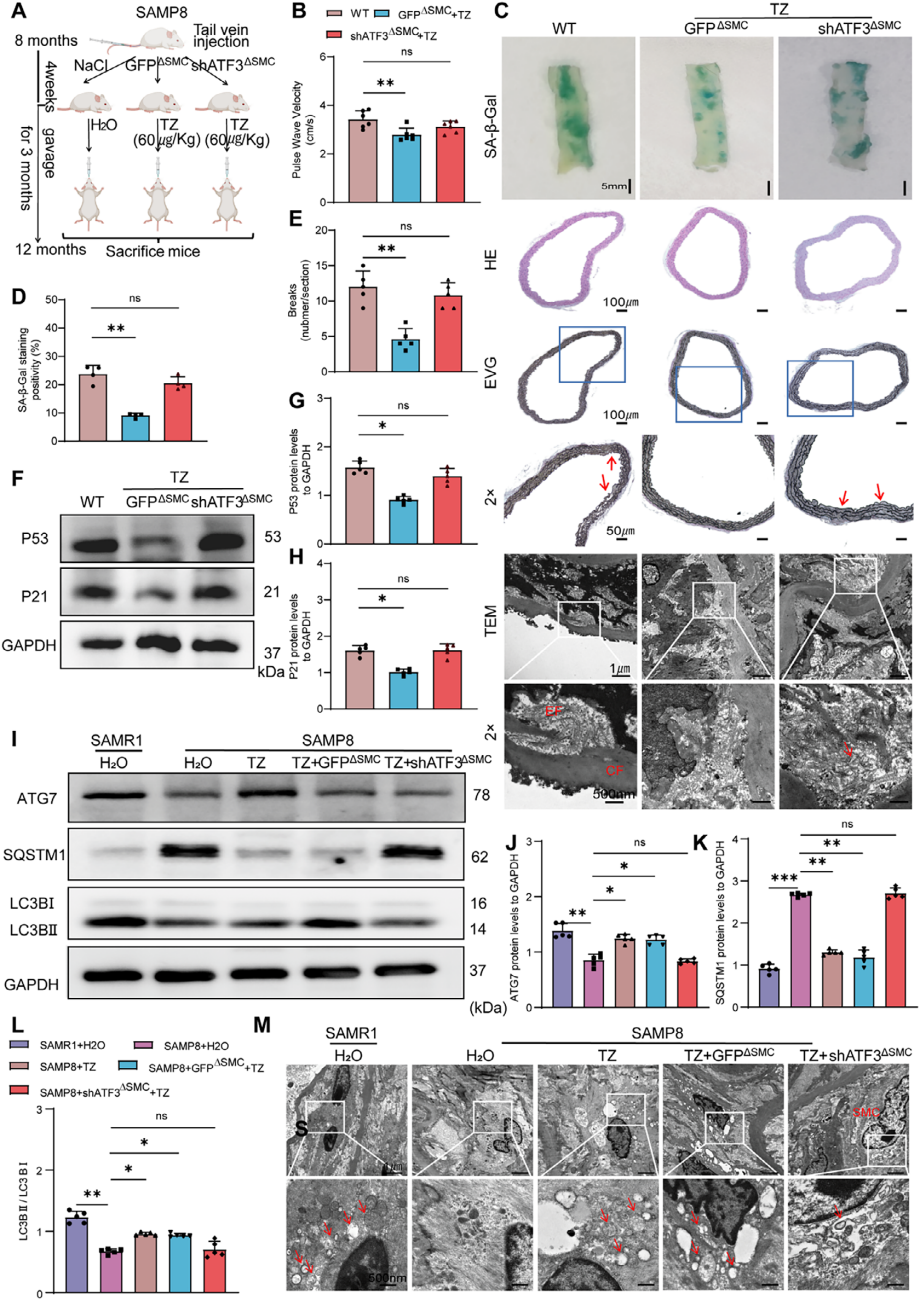
TZ improves VSMC senescence by promoting the autophagy signaling pathway via ATF3. A) Experimental design: SAMP8 mice were injected tail vein with adenoassociated virus. After 4 weeks, mice received TZ (60 µg kg^−1^) by oral gavage for 3 months (*n* = 6). B) PWV in SAMP8 mice (*n *= 6). C–E) SA‐β‐gal staining, Hematoxylin and eosin staining, EVG staining, and TEM of mouse aortas (scale bars, 5 mm, 50 µm, 100 µm, 1 µm, and 500 nm). EF: elastic fiber, CF: collagen fiber; arrows indicate areas of collagen fiber disarray (*n *= 5). F–H) Western blot analysis of p53 and p21 expression in mouse aortas (*n *= 5). I–L) Western blot analysis of LC3B‐II/LC3B‐I, SQSTM1, and ATG7 expression in mouse aortas (*n *= 5). M) TEM observation of autophagic phenomena in VSMCs in mouse aortas (*n *= 5); arrows indicate autophagosomes or autolysosomes (scale bars, 1 µm and 500 nm). Error bars represent mean ± standard deviation. The one‐way ANOVA was performed to compare data; *p < 0.05, **p < 0.01, ***p < 0.001.

To clarify whether the observed vascular results are dependent on the effects of TZ in ECs or VSMCs, we investigated the changes in the expression of ATF3 in ECs and VSMCs. According to the IF assay results, the level of VSMC‐labeled ATF3 protein decreased in SAMP8 mice and recovered after TZ intervention (Figure S10A, Supporting Information). Moreover, ATF3 expression in ECs was not affected by ageing or TZ intervention (Figure S10A, Supporting Information), indicating that VSMC‐derived ATF3 acts as a key regulator contributing to the protective effects of TZ. We administered TZ to SAMP8+GFP^∆SMC^ and SAMP8+shATF3^∆SMC^ mice for 3 months and divided the experimental groups into three categories (**Figure** **6**A).

SAMP8 mice treated with TZ rarely presented significant alterations in body weight, blood glucose levels, blood lipids, or abnormalities in liver and kidney histology (Table S6, Figure S10B, Supporting Information).

A comparison of the SAMP8+GFP^∆SMC^ +TZ and SAMP8+shATF3^∆SMC^+TZ groups revealed that decreased ATF3 expression significantly inhibited the regulatory effects of TZ and accelerated VSMC senescence and vascular ageing in SAMP8 mice. Compared with the SAMP8+GFP^∆SMC^+TZ group, the SAMP8+shATF3^∆SMC^+TZ group exhibited higher PWV (Figure 6B). ATF3 knockdown inhibited the reparative effect of TZ on vascular ageing and structure, as shown by SA‐β‐gal and EVG staining and TEM (Figure 6C–E). WB revealed a significant increase in senescence markers and synthetic phenotypic markers in the SAMP8+shATF3^∆SMC^+TZ group compared with those in the SAMP8+GFP^∆SMC^+TZ group (Figure 6F,H), with similar trends observed in the qPCR and IF assays (Figure S10C–E, Supporting Information).

In vitro studies also demonstrated this effect. ATF3‐knockdown siRNA blocked the beneficial effects of TZ on anti‐ageing and anti‐phenotype switching in senescent VSMCs (Figure S11, Supporting Information).

### 2.7 TZ Reduced VSMC Senescence and Vascular Ageing through the Autophagy Signaling Pathway

We examined the variations in the autophagy pathway after the administration of TZ. TZ increased ATG7 levels, promoted the conversion of LC3‐II to LC3‐I, and enhanced SQSTM1 degradation in SAMP8 mice, as shown by WB (Figure 6I–L). This trend was reversed by the absence of ATF3 (Figure 6I–L). Compared with SAMR1 mice, SAMP8 mice showed fewer APs in the vascular media under TEM. When ATF3 was expressed, TZ led to an increased number of APs (Figure 3M). These results showed that TZ administration activated autophagy in an ATF3‐dependent manner.

In vitro experiments were conducted to confirm the above findings. TZ elevated ATG7 mRNA and protein levels in senescent VSMCs, facilitating the degradation of SQSTM1 (Figure S12A–D, Supporting Information). TZ further increased the LC3B‐II/LC3B‐I ratio in the presence of BafA1. However, ATF3 silencing prevented TZ from promoting LC3‐II formation (Figure S12E,F, Supporting Information). Both TEM and LC3 virus transfection revealed that TZ increased the AP content, whereas ATF3 knockdown reversed this effect (Figure S12G,H, Supporting Information). Taken together, these results suggest that TZ treatment activates autophagic flux via ATF3.

To investigate whether TZ played a role in regulating cellular ageing and phenotype switching through the ATG7/LC3 signaling pathway, ATG7 and LC3 were inhibited in VSMCs. In ATG7‐depleted senescent VSMCs, TZ failed to decrease the expression of ageing markers or significantly reverse the contraction‐secretion phenotype transition (Figure S13, Supporting Information). DC‐LC3in‐D5, an LC3 lipidation inhibitor^[^ ^44^ ^]^ suppresses cellular autophagy and exacerbates cellular senescence. The inhibition of autophagy led to VSMC senescence and secretory phenotype transition, a change that could not be reversed by TZ treatment, as shown by the results of the qPCR, WB, IF assays (Figure S14, Supporting Information). These findings indicate that the inhibition effect of TZ on VSMC senescence depended on the activation of autophagic flux.

### 2.8 TZ Improves AS by Ameliorating VSMC Senescence

**Figure 7:**
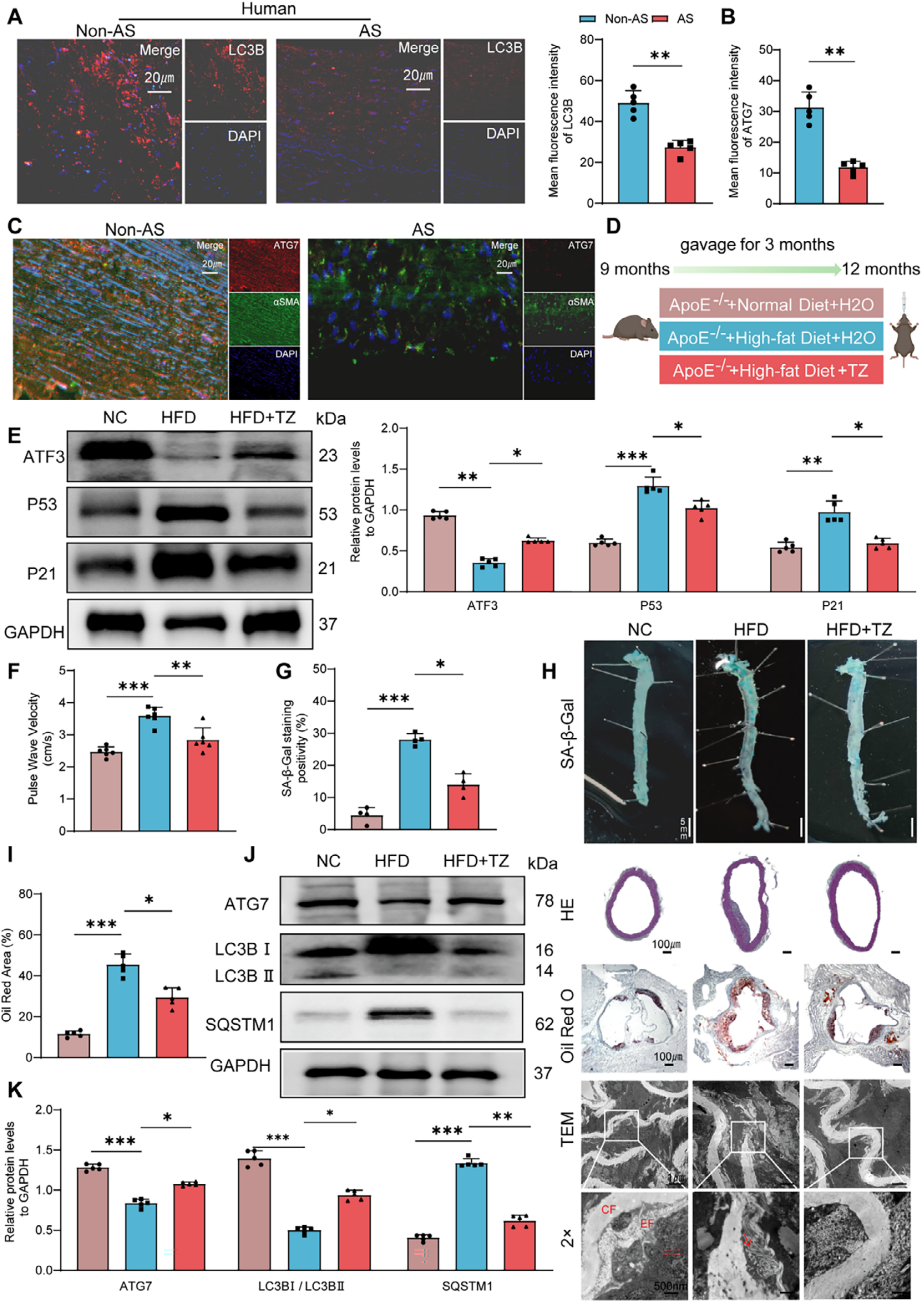
TZ improves age‐related phenotypes in ApoE^‐/‐^ mice. A) Immunofluorescent staining of LC3B (red) and nuclei (blue) in human carotid arteries (scale bars, 20 µm; *n *= 5). B,C) Immunofluorescent staining of ATG7 (red), α‐SMA (green), and nuclei (blue) in human carotid arteries (scale bars, 20 µm; *n *= 5). D) Experimental design: Administered TZ via gavage to in APOE^‐/‐^ mice fed with a High‐fat diet for three months (TZ: 60 µg kg^−1^, *n *= 8–10). E) Western blot analysis of ATF3, p53, and p21 expression in mouse aortas (*n *= 5). F) PWV in ApoE^‐/‐^ mice (*n *= 8–10). G–I) SA‐β‐gal staining, Hematoxylin and eosin staining, Oil Red O staining, and TEM of mouse aortas (scale bars, 5 mm, 100 µm, 1 µm, and 500 nm). EF: elastic fiber, CF: collagen fiber; arrows indicate areas of collagen fiber disarray (*n* = 5). J,K) Western blot analysis of LC3B‐II/LC3B‐I, SQSTM1, and ATG7 expression in mouse aortas (*n *= 5). Error bars represent mean ± standard deviation. The unpaired *t*‐test (A,B) and one‐way ANOVA (E,F,G,I,K) were performed to compare data; *p < 0.05, **p < 0.01, ***p < 0.001.

We verified the ATG7/LC3 signaling pathway in the atherosclerotic vasculature. IF analysis revealed that the abundance of LC3 spots in atherosclerotic plaques was reduced, indicating decreased autophagy activity in AS human samples (**Figure** **7**A).

ATG7 expression was lower in atherosclerotic plaque samples than in healthy control samples (Figure 7B,C). Additionally, to elucidate the impact of TZ on senescent VSMCs within the context of AS, we adopted an AS model in which ApoE^‐/‐^ mice were fed a HFD (Figure 7D). As anticipated, the AS model mice exhibited overt signs of vascular senescence, as evidenced by elevated expression of VSMC senescence markers (Figure 7E; Figure S15A–C, Supporting Information) and increases in PWV (Figure 7F) and the senescent cell fraction (Figure 7G,H). Treatment with TZ increased the ATF3 content in AS model mice and ameliorated VSMC senescence (Figure 7E). We also evaluated the vascular morphology of ApoE^‐/‐^ mice via HE and EVG staining, TEM analysis, and lipid deposition. TZ significantly decreased elastic fiber rupture at the plaque site and the Oil Red O‐positive staining area in the aortic valve (Figure 7H,I; Figure S15D,E, Supporting Information). Remarkably, TZ ameliorated the extent of vascular ageing without significantly lowering the BP of ApoE^‐/‐^ mice (Figure S15F, Supporting Information). mRNA and protein analyses collectively revealed that TZ countered VSMC phenotypic switching during AS development (Figure S15G–L, Supporting Information). TZ demonstrated robust safety and therapeutic efficacy in AS mouse models.

TZ restored the diminished ATG7 levels associated with AS progression in ApoE^‐/‐^ mice (Figure 7J,K). Similar changes were observed by qPCR and IF (Figure S16A,B, Supporting Information). Furthermore, TEM revealed diminished AP formation within the VSMCs of AS model mice. However, this effect was reversed upon TZ administration (Figure S16C, Supporting Information). WB analysis confirmed that atherosclerotic conditions were associated with reduced formation of LC3B‐II and increased SQSTM1 in VSMCs, and these effects were reversed by TZ treatment (Figure 7J,K). Taken together, these data indicate that TZ activates autophagy through the ATF3/ATG7/LC3 signaling pathway, ultimately delaying VSMC senescence and reducing AS.

## 3 Discussion

This study demonstrated that the lack of ATF3 in VSMCs contributes to AS and VSMC senescence. ATF3 activates the transcription of ATG7, promoting autophagy to improve VSMC senescence. A new target of TZ is to maintain the stability of *Atf3* mRNA to exert anti‐AS effects. ATF3 knockdown or autophagy inhibition negates the vascular protective effects of TZ.

VSMC senescence has been implicated as a contributing factor in the development of diverse vascular pathologies.^[^ ^45^ ^]^ AS is characterized by premature vascular ageing,^[^ ^46^ ^]^ particularly in atherosclerotic plaque areas in the aorta. Senescent VSMCs exacerbate plaque growth and increase the risk of rupture.^[^ ^47^ ^]^ Anti‐senescence strategies, such as histone deacetylase‐modulating agents^[^ ^48^ ^]^ and vitamin D,^[^ ^49^ ^]^ have been explored to ameliorate vascular dysfunction in AS.

ATF3 is vital in cellular functions and decreases in expression with age. Its activity and expression are influenced by transcriptional regulation, post‐transcriptional control, post‐translational modification, and the cellular milieu.^[^ ^37^ ^]^ Wang et al. demonstrated that inactivation of ATF3 results in VSMC dedifferentiation, causing loss of the contractile phenotype.^[^ ^50^ ^]^ Other studies have reported the beneficial actions of ATF3 in the context of AS. Hepatocyte‐derived ATF3 exerts an inhibitory effect on AS development through its ability to modulate lipid and bile acid metabolism.^[^ ^51^ ^]^ We observed the greatest changes in ATF3 expression in VSMCs from AS patients compared with those from baseline controls, while ECs from AS patients showed no significant alterations (in ATF3 expression) compared with their baseline counterparts. In addition, ATF3 levels were significantly decreased in the VSMCs of aged mice. The expression distributions of ATF3 and p53 in VSMCs were negatively correlated. Moreover, additional knockdown of ATF3 in VSMCs exacerbated AS in APOE^‐/‐^ mice through accelerated VSMC senescence.

Autophagic activity decreases in the vascular tissues of elderly mice and humans,^[^ ^32^ ^]^ increasing the risk of arterial stiffness and vascular calcification. Impaired VSMC autophagy in APOE^‐/‐^ mice with SMC‐specific ATG7 deficiency accelerates plaque formation.^[^ ^52^ ^]^ Previous studies have shown that inducing autophagy can suppress VSMC phenotypic transformation and migration induced by oxidized low‐density lipoprotein.^[^ ^53^ ^]^ The modulatory effect on VSMC phenotypic transformation induced by high uric acid can be reversed, delaying AS.^[^ ^54^, ^55^ ^]^ However, the dual role of autophagy in organisms^[^ ^56^ ^]^ requires great caution in the administration of autophagy‐modulating drugs. Our findings align with those of other studies, suggesting that ATF3 acts as a potential novel pathway for autophagy regulation. ATF3 activation enhances autophagy upon bacterial infection, increasing the defense capabilities of the organism.^[^ ^57^ ^]^ ATF3 overexpression in mouse lung tissues following Mycobacterium infection facilitates the expression of autophagy‐related proteins while suppressing inflammation.^[^ ^57^ ^]^ However, impaired autophagy hinders the nuclear translocation of ATF3, compromising its anti‐inflammatory effects.^[^ ^58^ ^]^ Tumor cells lacking ATG7 or ATG5 exhibit downregulated ATF3 expression due to impaired autophagy.^[^ ^59^ ^]^ In our study, ATF3 interacted with ATG7 as determined by MS, Co‐IP, GST pull‐down, and co‐localization assays. ATG7 binds to the nuclear translocation region of ATF3 in the cytoplasm, and three key amino acids essential for binding in this region were confirmed. The role of ATG7 in modulating the nuclear entry of ATF3 was assessed through supplementary experiments. ATG7‐deficient VSMCs showed reduced nuclear ATF3 content with a more dispersed distribution, emphasizing the role of ATG7 in regulating ATF3 nuclear localization and distribution. Once in the nucleus, ATF3 binds to the ATG7 promoter area, facilitating its transcription and leading to the activation of autophagy. ATG7 promotes ATF3 nuclear translocation without concomitant nuclear translocation. This mechanism mirrors established regulators of ATF transcription factors, such as 14‐3‐3 proteins that dissociate prior to nuclear entry.^[^ ^60^ ^]^

Depletion of ATF3 leads to a decline in autophagic flow. In VSMCs transfected with AdV carrying the GFP‐mCherry‐LC3 fusion protein, APs that do not fuse with lysosomes exhibit yellow fluorescence, whereas autolysosomes display red fluorescence. A deficiency reduces the levels of both fluorescence types, with a notable decrease in yellow fluorescence.

VSMC senescence is accompanied by increased m6A methylation of *Atf3* mRNA and decreased mRNA stability. By employing YTHDF2 to recognize the binding patterns of *Atf3* mRNA m6A sites, we performed high‐throughput virtual screening and discovered that TZ could potentially change their affinity. Through RIP assay and mRNA stability analysis, TZ was shown to increase the expression of ATF3 by reducing the binding affinity of YTHDF2 for m6A‐containing *Atf3* mRNAs. Molecular dynamics simulations further confirmed that adding TZ destabilizes the binding between YTHDF2 and ATF3 mRNA. Additionally, sequencing analysis of vascular cells from TZ‐treated SAMP8 mice revealed that DEGs were predominantly enriched in the ATF3‐CHOP signaling pathway. qPCR demonstrated that TZ does not alter the mRNA levels of other ATF family members. Collectively, these findings suggest the specificity of TZ in targeting ATF3 mRNA. Additionally, ATF3 expression in ECs remained unaltered by ageing or TZ treatment. In contrast to that in VSMCs, ATF3 mRNA in ECs is inherently highly stable and is not further extended by the TZ. A plausible explanation is that the m6A modification of ATF3 mRNA in ECs remains unaltered during ageing.

The clinical safety of TZ, a widely used drug, is well‐accepted. TZ has demonstrated significant benefits in various diseases, particularly neurodegenerative disorders, such as amyotrophic lateral sclerosis and Parkinson's disease.^[^ ^61^ ^]^ However, there is a relative dearth of data regarding the benefits of TZ for cardiovascular diseases. These findings suggest that the ATF3‐autophagy signaling axis may serve as a potential molecular target for TZ in AS intervention. In our previous pilot study, low‐dose TZ significantly improved arterial stiffness, as evidenced by reduced brachial‐ankle pulse wave velocity, with the beneficial effects persisting throughout a one‐year follow‐up period.^[^ ^62^ ^]^ On this basis, larger randomized controlled trials evaluating the efficacy of TZ in treating AS are still needed. By integrating these preliminary clinical observations with our experimental findings, this work provides foundational data and a feasibility assessment for the design of future large‐scale, in‐depth clinical trials. Knocking down ATF3 weakens the anti‐ageing effect of TZ. Moreover, TZ reduces VSMC senescence and phenotypic transformation through an autophagy‐dependent pathway, and autophagy inhibitors block the anti‐aging effects of TZ.

This study provides strong evidence and valuable insights into the relationship between VMSC senescence and the ATF3/ATG7/LC3 signaling pathway. While the findings showed a trend toward reduced BP with low‐dose TZ, they did not reach statistical significance. In particular, the TZ dose used in our rodent experiments, when translated into human equivalent doses, is far below the recommended dose for clinical use to lower BP according to the expert consensus‐based clinically equivalent dose estimates and dosing recommendations.^[^ ^63^ ^]^ Therefore, the downward tendency in blood pressure in ageing‐reversed mice might be attributed to improved vascular compliance due to reduced vascular stiffness. Our data demonstrate that TZ exerts anti‐ageing effects distinct from its α1‐AR antagonism. Pre‐treatment with phen fully abrogated TZ's α1‐AR antagonism, yet TZ retained its ability to rescue senescence markers in VSMCs. In contrast, DZ—a structurally similar α1‐AR antagonist—failed to mitigate cellular senescence at equimolar doses, despite exhibiting comparable receptor binding affinity. Future studies are still needed to examine the anti‐ageing effects of hypotensors across various tissues and cell types. The current data primarily provide theoretical foundations for clinical translation, and further translational medical studies are needed to validate TZ's clinical efficacy. Future research could focus on the following directions. Cross‐Species Validation: Expanding preclinical evaluation by assessing the efficacy of TZ in ageing‐related AS using large animal models (e.g., non‐human primates), with a focus on defining its dose‐response relationships and ATF3 dependency. Clinical correlation analysis: Retrospective cohort studies to compare plaque progression in AS patients treated with TZ with that in patients treated with other antihypertensive agents, and histological analyses of vascular tissues to quantify ATF3‐autophagy pathway activity and explore its clinical relevance.

## 4 Experimental Section

### Human Samples

A case‐control study involving human biological sample collection was performed. Five carotid artery samples were procured from the sample repository maintained by the Department of Cardiovascular Surgery at Tongji Hospital from 2020 to 2022. Control samples were acquired from three healthy donors who underwent heart transplantation. Table S1 (Supporting Information) summarizes the characteristics of the patients. The Ethics Committee of Tongji Hospital, Huazhong University of Science and Technology (approval number: TJ‐IRB20191215) approved the study. All experiments were performed in accordance with the principles expressed in the Declaration of Helsinki, and informed consent was obtained from all participants.

### Animals

The animal experiments were approved by the Experimental Animal Center of Huazhong University of Science and Technology (20 220 981). All animal procedures conformed to the current National Institution of Health guidelines.

Eight‐month‐old male SAMR1 mice and SAMP8 mice weighing 28—32 g were obtained from the Experimental Animal Science Department, Peking University School of Medicine, Beijing, China. Additionally, 8‐week‐old ApoE^‐/‐^ male mice weighing 25–28 g and 18‐month‐old C57BL/6 male mice weighing 30–35 g were purchased from Guangdong Yaokang Biotechnology Company.

ApoE^‐/‐^ mice were fed a HFD comprising 19.97% protein and 39.85% fat (Special Diet Services, XTZ08C, Xietong Bio‐engineering, Jiangsu, China) for 12 weeks to induce advanced plaques. The mice were housed under conventional and specific pathogen‐free conditions. Isoflurane, a volatile anesthetic agent, was used to induce general anesthesia in the mice once a month. The mice were euthanized via intraperitoneal injection of pentobarbital sodium (1%, 0.2 mL), and the hearts were subsequently perfused with phosphate‐buffered saline. The detailed methods are provided in the Supporting Information.

### TZ Administration

TZ (T4680, Sigma‐Aldrich, USA, sigmaaldrich.cn/CN/zh/product/sigma/t4680), with a purity of 99.7%, was used in this experiment. The molecular structure (www.chemblink.com) and chromatographic identification of TZ are in Figure S1A,B (Supporting Information). TZ was dissolved in distilled water and administered to mice by oral gavage, with the control group receiving an equivalent volume of distilled water. The TZ dosages were divided into three groups based on previous reports and preliminary designs: 20 µg kg^−1^ (TZ1 group), 60 µg kg^−1^ (TZ2 group), and 180 µg kg^−1^ (TZ3 group).

## Conflict of Interest

The authors declare no conflict of interest.

## Author Contributions

H.N. and T.J. contributed equally to this work and shared first authorship. H.N. and T.J. was dealt with conceptualization, investigation, validation, and writing original draft. J.Y. and C.Z. dealt with funding acquisition, writing review and editing, conceived the study, and designed the experiments. Y.H., J.H., H.L., L.Z., and Z.Y. dealt with data analysis. W.L., Y.G., Y.L., L.Z., and L.R. dealt with supervision.

## Supporting information



Supporting Information

Supporting Information

Supporting Information

Supporting Information

## Data Availability

The data that support the findings of this study are available from the corresponding author upon reasonable request.

## References

[1] S. Du , H. Ling , Z. Guo , Q. Cao , C. Song , Pharmacol. Res. 2021, 165, 105278. 33166733 10.1016/j.phrs.2020.105278

[2] J. C. Wang , M. Bennett , Circ. Res. 2012, 111, 245. 22773427 10.1161/CIRCRESAHA.111.261388

[3] Q. Xiang , F. Tian , J. Xu , X. Du , S. Zhang , L. Liu , Biol. Rev. Cambridge Philos. Soc. 2022, 97, 1844. 35569818 10.1111/brv.12866 PMC9541442

[4] P. Boutouyrie , P. Chowienczyk , J. D. Humphrey , G. F. Mitchell , Circ. Res. 2021, 128, 864. 33793325 10.1161/CIRCRESAHA.121.318061

[5] U. Ocak , M. T. Özdal , Brain Hemorrh. 2024, 5, 46.

[6] M. Tesauro , A. Mauriello , V. Rovella , M. Annicchiarico‐Petruzzelli , C. Cardillo , G. Melino , N. Di Daniele , J. Intern. Med. 2017, 281, 471. 28345303 10.1111/joim.12605

[7] T. J. Guzik , R. M. Touyz , Hypertension 2017, 70, 660. 28784646 10.1161/HYPERTENSIONAHA.117.07802

[8] S. Liu , H. Ding , Y. Li , X. Zhang , J. Cardiovasc. Dev. Dis. 2022, 9, 459. 36547457 10.3390/jcdd9120459 PMC9782920

[9] Y. Inaba , E. Hashiuchi , H. Watanabe , K. Kimura , Y. Oshima , K. Tsuchiya , S. Murai , C. Takahashi , M. Matsumoto , S. Kitajima , Y. Yamamoto , M. Honda , S. I. Asahara , K. Ravnskjaer , S. I. Horike , S. Kaneko , M. Kasuga , H. Nakano , K. Harada , H. Inoue , Nat. Commun. 2023, 14, 167. 36690638 10.1038/s41467-023-35804-w PMC9871012

[10] J. Mao , Q. Zhang , Y. Zhuang , Y. Zhang , L. Li , J. Pan , L. Xu , Y. Ding , M. Wang , Y. S. Cong , Nat. Aging 2024, 4, 1794. 39543280 10.1038/s43587-024-00745-6

[11] C. Zhang , X. Zhang , L. Huang , Y. Guan , X. Huang , X. L. Tian , L. Zhang , W. Tao , Aging Cell 2021, 20, 13315. 10.1111/acel.13315 PMC7963335 33539668

[12] Y. Du , P. Hu , X. Ding , D. Wang , J. Luo , S. Le , L. Ren , M. Chen , P. Ye , J. Xia , Clin. Transl. Med. 2025, 15, 70147. 10.1002/ctm2.70147 PMC11680558 39731276

[13] S. Kumariya , V. Ubba , R. K. Jha , J. R. Gayen , Autophagy 2021, 17, 2706. 34161185 10.1080/15548627.2021.1938914 PMC8526011

[14] I. Tanida , T. Ueno , E. Kominami , Methods Mol. Biol. 2008, 445, 77. 18425443 10.1007/978-1-59745-157-4_4

[15] K. Frudd , T. Burgoyne , J. R. Burgoyne , Nat. Commun. 2018, 9, 95. 29311554 10.1038/s41467-017-02352-z PMC5758830

[16] Y. Zheng , W. Sun , Z. Wang , J. Liu , C. Shan , C. He , B. Li , X. Hu , W. Zhu , L. Liu , F. Lan , C. Jiang , C. Zhao , X. Li , N. Sun , Research 2022, 2022, 9784081. 36405253 10.34133/2022/9784081 PMC9667885

[17] C. Koutouroushis , O. Sarkar , Cureus 2021, 13, 20042. 10.7759/cureus.20042 PMC8631374 34873555

[18] S. C. Nussenzweig , S. Verma , T. Finkel , Circ. Res. 2015, 116, 480. 25634971 10.1161/CIRCRESAHA.116.303805 PMC4313568

[19] S. Nair , J. Ren , Cell Cycle 2012, 11, 2092. 22580468 10.4161/cc.20317 PMC3368861

[20] X. Chen , C. Zhao , X. Li , T. Wang , Y. Li , C. Cao , Y. Ding , M. Dong , L. Finci , J. H. Wang , X. Li , L. Liu , Nat. Chem. Biol. 2015, 11, 19. 25383758 10.1038/nchembio.1657 PMC4412158

[21] J. E. Simmering , M. J. Welsh , L. Liu , N. S. Narayanan , A. Pottegård , JAMA Neurol. 2021, 78, 407. 33523098 10.1001/jamaneurol.2020.5157 PMC7851758

[22] Z. Y. Yu , X. Yi , Y. R. Wang , G. H. Zeng , C. R. Tan , Y. Cheng , P. Y. Sun , Z. H. Liu , Y. J. Wang , Y. H. Liu , J. Neurochem. 2022, 161, 293. 35244207 10.1111/jnc.15603

[23] F. Ye , R. F. Keep , Y. Hua , H. J. L. Garton , G. Xi , Brain Hemorrh. 2023, 4, 44. 10.1016/j.hest.2022.06.001 PMC10260252 37309451

[24] C. Chi , D. J. Li , Y. J. Jiang , J. Tong , H. Fu , Y. H. Wu , F. M. Shen , Biochim. Biophys. Acta, Mol. Basis Dis. 2019, 1865, 1810. 31109451 10.1016/j.bbadis.2018.08.015

[25] J. M. Miano , E. A. Fisher , M. W. Majesky , Circulation 2021, 143, 2110. 34029141 10.1161/CIRCULATIONAHA.120.049922 PMC8162373

[26] J. Shi , Y. Yang , A. Cheng , G. Xu , F. He , Am. J. Physiol.: Heart Circ. Physiol. 2020, 319, H613. 32762559 10.1152/ajpheart.00220.2020

[27] P. Lacolley , V. Regnault , A. P. Avolio , Cardiovasc. Res. 2018, 114, 513. 29514201 10.1093/cvr/cvy009

[28] V. Karuppagounder , S. Arumugam , S. S. Babu , S. S. Palaniyandi , K. Watanabe , J. P. Cooke , R. A. Thandavarayan , Ageing Res. Rev. 2017, 35, 291. 27825897 10.1016/j.arr.2016.10.006 PMC12142494

[29] S. Laurent , L. Marais , P. Boutouyrie , Can. J. Cardiol. 2016, 32, 669. 27118294 10.1016/j.cjca.2016.01.039

[30] A. Lin , J. M. Miano , E. A. Fisher , A. Misra , Nat. Cardiovasc. Res. 2024, 3, 1408. 39653823 10.1038/s44161-024-00569-y

[31] A. González‐Moro , E. Herranz , M. M. Rodríguez de Lope , I. R. Sanchez‐Pajares , J. Sánchez‐Ramírez , A. Rivera‐Tenorio , L. Shamoon , C. F. Sánchez‐Ferrer , C. Peiró , F. de la Cuesta , Life Sci. 2025, 369, 123529. 40049367 10.1016/j.lfs.2025.123529

[32] M. Abdellatif , S. Sedej , D. Carmona‐Gutierrez , F. Madeo , G. Kroemer , Circ. Res. 2018, 123, 803. 30355077 10.1161/CIRCRESAHA.118.312208

[33] J. R. Burgoyne , Autophagy 2018, 14, 1092. 29746182 10.1080/15548627.2018.1444311 PMC6103406

[34] D. H. Lee , J. S. Park , Y. S. Lee , J. Han , D. K. Lee , S. W. Kwon , D. H. Han , Y. H. Lee , S. H. Bae , Autophagy 2020, 16, 1949. 31913745 10.1080/15548627.2020.1712108 PMC7595589

[35] Y. Xing , X. Wei , Y. Liu , M. M. Wang , Z. Sui , X. Wang , W. Zhu , M. Wu , C. Lu , Y. H. Fei , Y. Jiang , Y. Zhang , Y. Wang , F. Guo , J. L. Cao , J. Qi , W. Wang , Autophagy 2022, 18, 1932. 34878954 10.1080/15548627.2021.2008752 PMC9450983

[36] M. Huang , X. Wang , M. Zhang , Y. Liu , Y. G. Chen , Cell Regener. 2024, 13, 14. 10.1186/s13619-024-00197-8 PMC11297012 39093347

[37] J. J. Xuan , W. J. Sun , P. H. Lin , K. R. Zhou , S. Liu , L. L. Zheng , L. H. Qu , J. H. Yang , Nucleic Acids Res. 2018, 46, D327. 29040692 10.1093/nar/gkx934 PMC5753293

[38] Y. Zhou , P. Zeng , Y. H. Li , Z. Zhang , Q. Cui , Nucleic Acids Res. 2016, 44, 91. 10.1093/nar/gkw104 PMC4889921 26896799

[39] X. Liu , J. Yuan , X. Zhang , L. Li , X. Dai , Q. Chen , Y. Wang , Chem. Res. Toxicol. 2021, 34, 1814. 34213887 10.1021/acs.chemrestox.1c00206 PMC8756675

[40] A. Bielak‐Zmijewska , M. Wnuk , D. Przybylska , W. Grabowska , A. Lewinska , O. Alster , Z. Korwek , A. Cmoch , A. Myszka , S. Pikula , G. Mosieniak , E. Sikora , Biogerontology 2014, 15, 47. 24243065 10.1007/s10522-013-9477-9 PMC3905196

[41] S. Mišúth , M. Uhrinová , J. Klimas , D. Vavrincová‐Yaghi , P. Vavrinec , Vasc. Pharmacol. 2021, 138, 106855. 10.1016/j.vph.2021.106855 33744414

[42] J. Li , S. G. Kim , J. Blenis , Cell Metab. 2014, 19, 373. 24508508 10.1016/j.cmet.2014.01.001 PMC3972801

[43] M. V. Blagosklonny , Cell Cycle 2022, 21, 1456. 35358003 10.1080/15384101.2022.2054636 PMC9278457

[44] S. Fan , L. Yue , W. Wan , Y. Zhang , B. Zhang , C. Otomo , Q. Li , T. Lin , J. Hu , P. Xu , M. Zhu , H. Tao , Z. Chen , L. Li , H. Ding , Z. Yao , J. Lu , Y. Wen , N. Zhang , M. Tan , K. Chen , Y. Xie , T. Otomo , B. Zhou , H. Jiang , Y. Dang , C. Luo , Angew. Chem. 2021, 60, 26105. 34590387 10.1002/anie.202109464 PMC8845813

[45] M. S. Chen , R. T. Lee , J. C. Garbern , Cardiovasc. Res. 2022, 118, 1173. 33963378 10.1093/cvr/cvab161 PMC8953446

[46] B. E. Veseli , P. Perrotta , G. R. A. De Meyer , L. Roth , C. Van der Donckt , W. Martinet , G. R. Y. De Meyer , Eur. J. Pharmacol. 2017, 816, 3. 28483459 10.1016/j.ejphar.2017.05.010

[47] S. Potteaux , C. Combadière , B. Esposito , C. Lecureuil , H. Ait‐Oufella , R. Merval , P. Ardouin , A. Tedgui , Z. Mallat , Arterioscler., Thromb., Vasc. Biol. 2006, 26, 1858. 16763157 10.1161/01.ATV.0000231527.22762.71

[48] M. O. J. Grootaert , M. R. Bennett , Nat. Rev. Cardiol. 2022, 19, 668. 35354967 10.1038/s41569-022-00685-x

[49] F. Carbone , L. Liberale , P. Libby , F. Montecucco , Eur. Heart J. 2023, 44, 2078. 36943351 10.1093/eurheartj/ehad165 PMC10281557

[50] Y. Wang , H. Gao , F. Wang , Z. Ye , M. Mokry , A. W. Turner , J. Ye , S. Koplev , L. Luo , T. Alsaigh , S. S. Adkar , M. Elishaev , X. Gao , L. Maegdefessel , J. L. M. Björkegren , G. Pasterkamp , C. L. Miller , E. G. Ross , N. J. Leeper , Cardiovasc. Res. 2021, 118, 2792. 10.1093/cvr/cvab347 PMC9586565 34849613

[51] Y. Xu , Y. Li , K. Jadhav , X. Pan , Y. Zhu , S. Hu , S. Chen , L. Chen , Y. Tang , H. H. Wang , L. Yang , D. Q. Wang , L. Yin , Y. Zhang , Nat. Metab. 2021, 3, 59. 33462514 10.1038/s42255-020-00331-1 PMC7856821

[52] Y. Osonoi , T. Mita , K. Azuma , K. Nakajima , A. Masuyama , H. Goto , Y. Nishida , T. Miyatsuka , Y. Fujitani , M. Koike , M. Mitsumata , H. Watada , Autophagy 2018, 14, 1991. 30025494 10.1080/15548627.2018.1501132 PMC6152523

[53] G. Wang , Y. Zhu , K. Li , B. Liao , F. Wang , L. Shao , L. Huang , D. Bai , J. Cardiovasc. Pharmacol. 2021, 78, 308. 34091481 10.1097/FJC.0000000000001069 PMC8340951

[54] M. O. J. Grootaert , M. Moulis , L. Roth , W. Martinet , C. Vindis , M. R. Bennett , G. R. Y. De Meyer , Cardiovasc. Res. 2018, 114, 622. 29360955 10.1093/cvr/cvy007

[55] Y. Lu , H. Zhang , M. Han , P. Wang , L. Meng , Pharmacology 2024, 109, 34. 38011839 10.1159/000534929

[56] N. Mizushima , M. Komatsu , Cell 2011, 147, 728. 22078875 10.1016/j.cell.2011.10.026

[57] I. Tattoli , M. T. Sorbara , D. J. Philpott , S. E. Girardin , Autophagy 2012, 8, 1848. 22932645 10.4161/auto.21863 PMC3541301

[58] B. Zhang , H. Li , J. Zhang , Y. Hang , Y. Xu , Tuberculosis 2022, 135, 102227. 35841815 10.1016/j.tube.2022.102227

[59] A. Aguirre , I. López‐Alonso , A. González‐López , L. Amado‐Rodríguez , E. Batalla‐Solís , A. Astudillo , J. Blázquez‐Prieto , A. F. Fernández , J. A. Galván , C. C. dos Santos , G. M. Albaiceta , J. Mol. Med. 2014, 92, 665. 24535031 10.1007/s00109-014-1132-7

[60] H. Chen , H. Zhang , P. Chen , S. Xiang , J. Mol. Biol. 2021, 433, 166874. 33556406 10.1016/j.jmb.2021.166874

[61] H. Chaytow , E. Carroll , D. Gordon , Y. T. Huang , D. van der Hoorn , H. L. Smith , T. Becker , C. G. Becker , K. M. E. Faller , K. Talbot , T. H. Gillingwater , EBioMedicine 2022, 83, 104202. 35963713 10.1016/j.ebiom.2022.104202 PMC9482929

[62] Y. Guan , Y. Zhang , L. Chen , Y. Ren , H. Nie , T. Ji , J. Yan , C. Zhang , L. Ruan , J. Cell. Mol. Med. 2024, 28, 18547. 10.1111/jcmm.18547 PMC11265993 39044238

[63] P. K. Whelton , R. M. Carey , JAMA 2017, 318, 2073. 29159375 10.1001/jama.2017.18209

