# Supplementary material for: ATF3 Deficiency Exacerbates Ageing‐Induced Atherosclerosis and Clinical Intervention Strategy
Source: Adv Sci (Weinh). 2025 Jul 11;12(37):e02249. doi: 10.1002/advs.202502249 (PMC12499416; doi:10.1002/advs.202502249)
Supplement: Supplementary file 1 — Supporting Information [file ADVS-12-e02249-s002.docx]

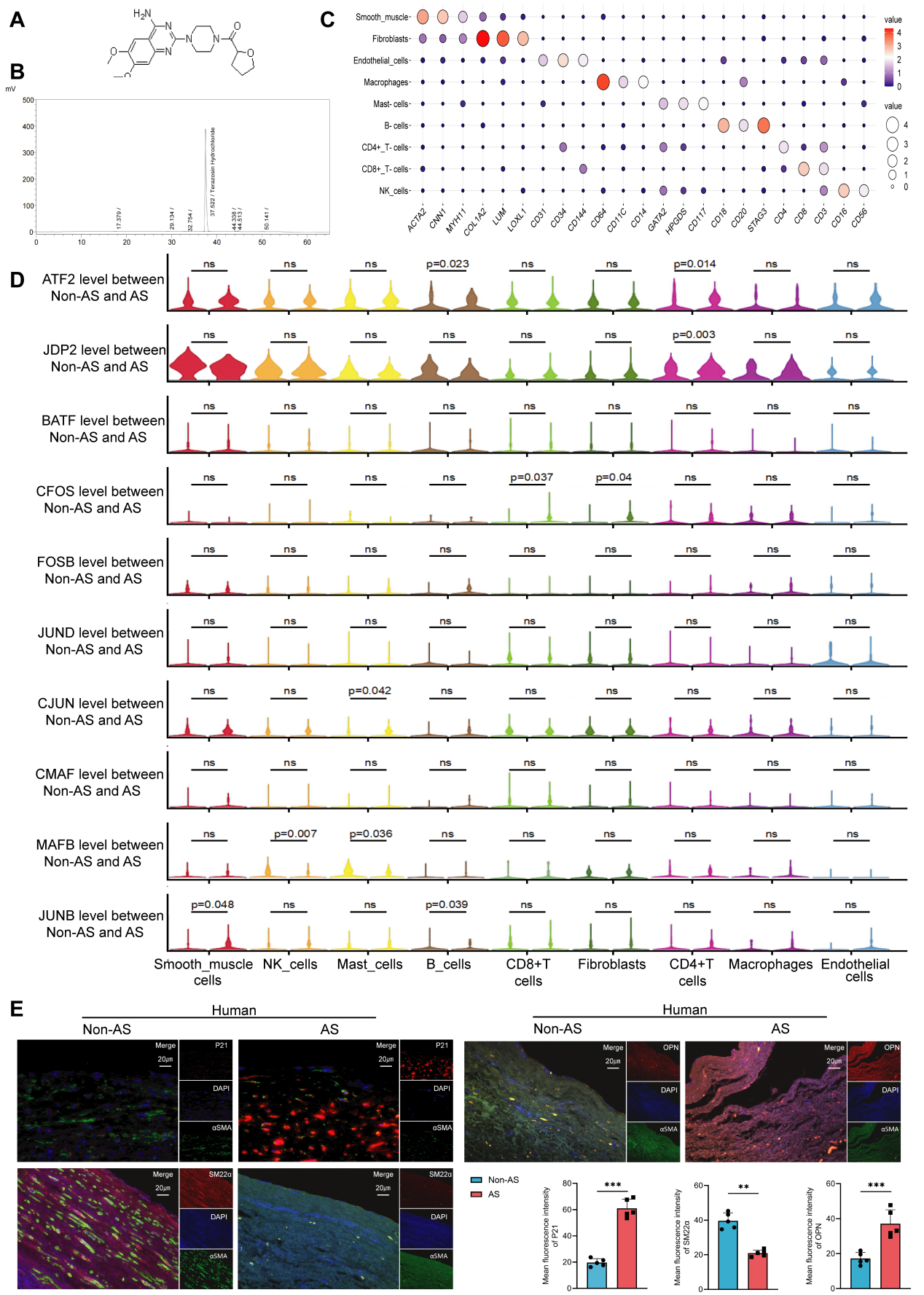
Figure S1


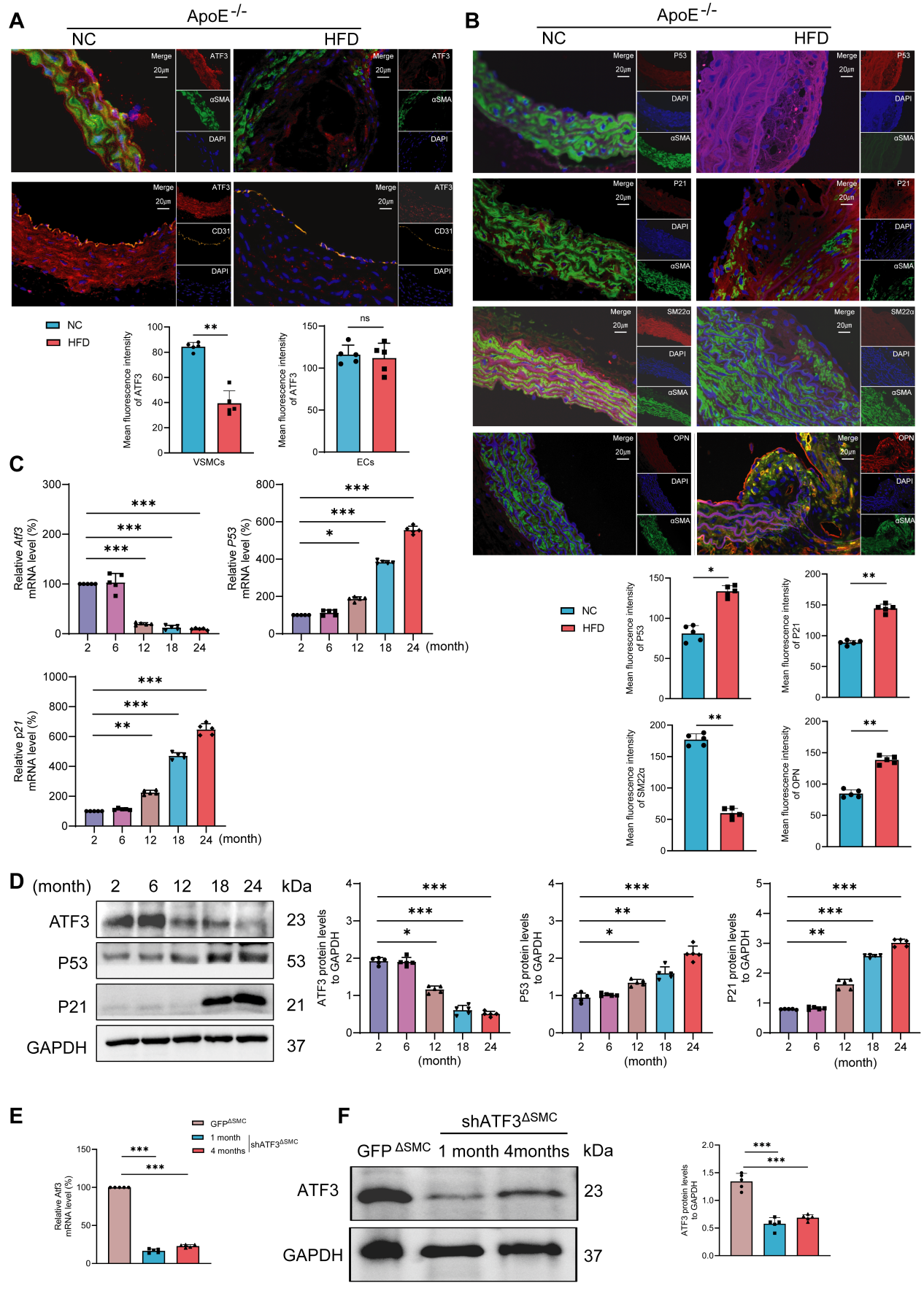
Figure S2


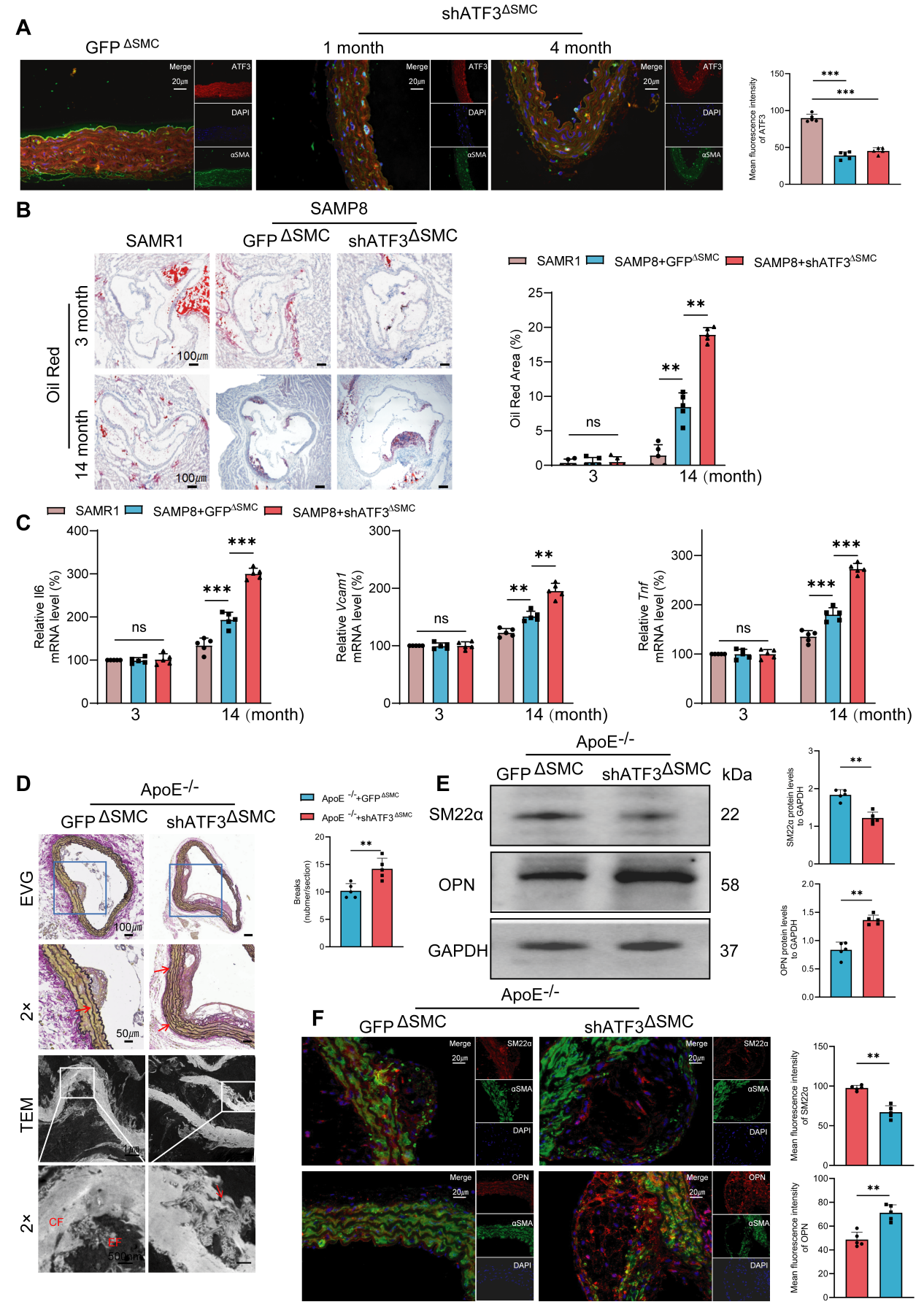
Figure S3


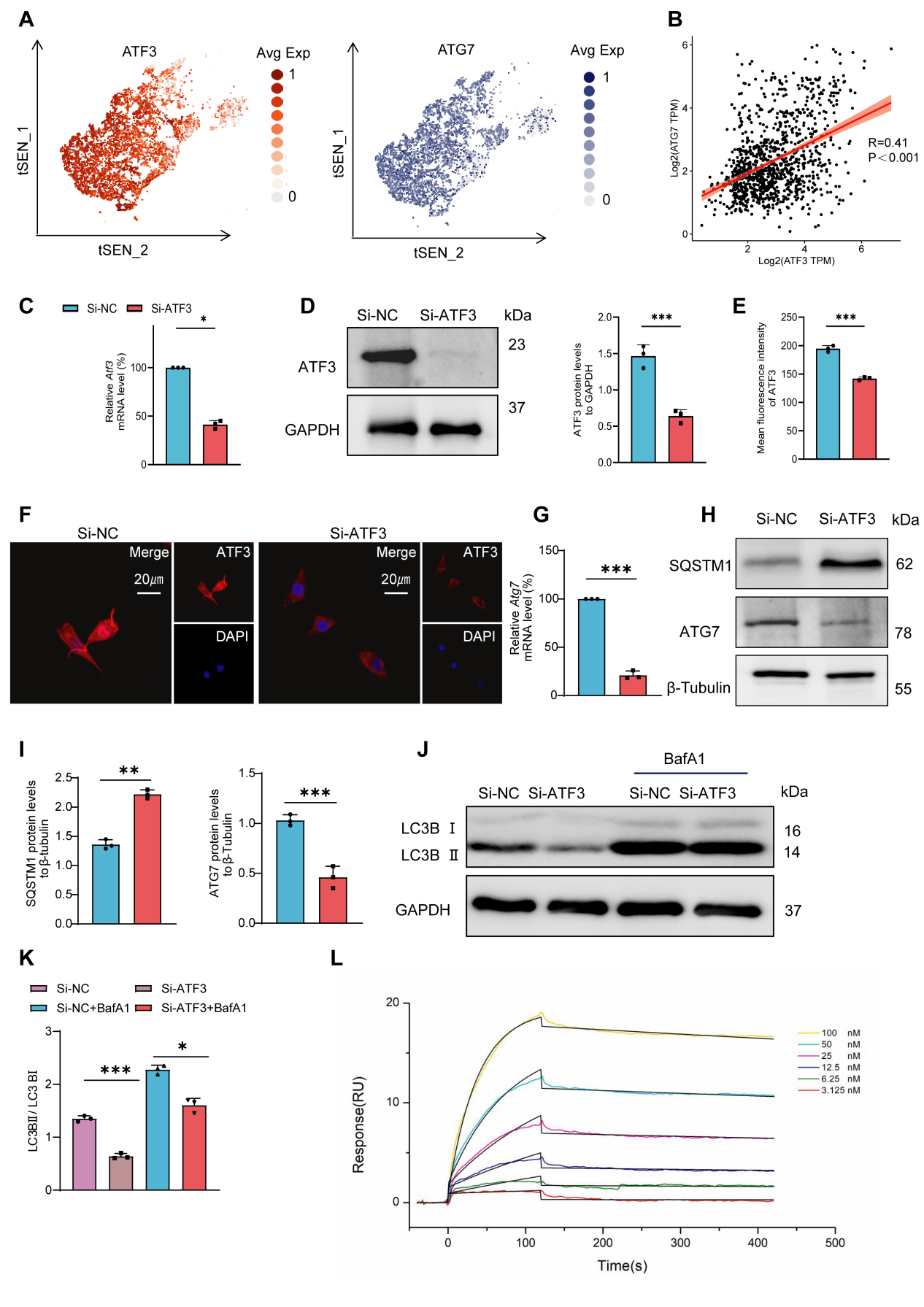
Figure S4


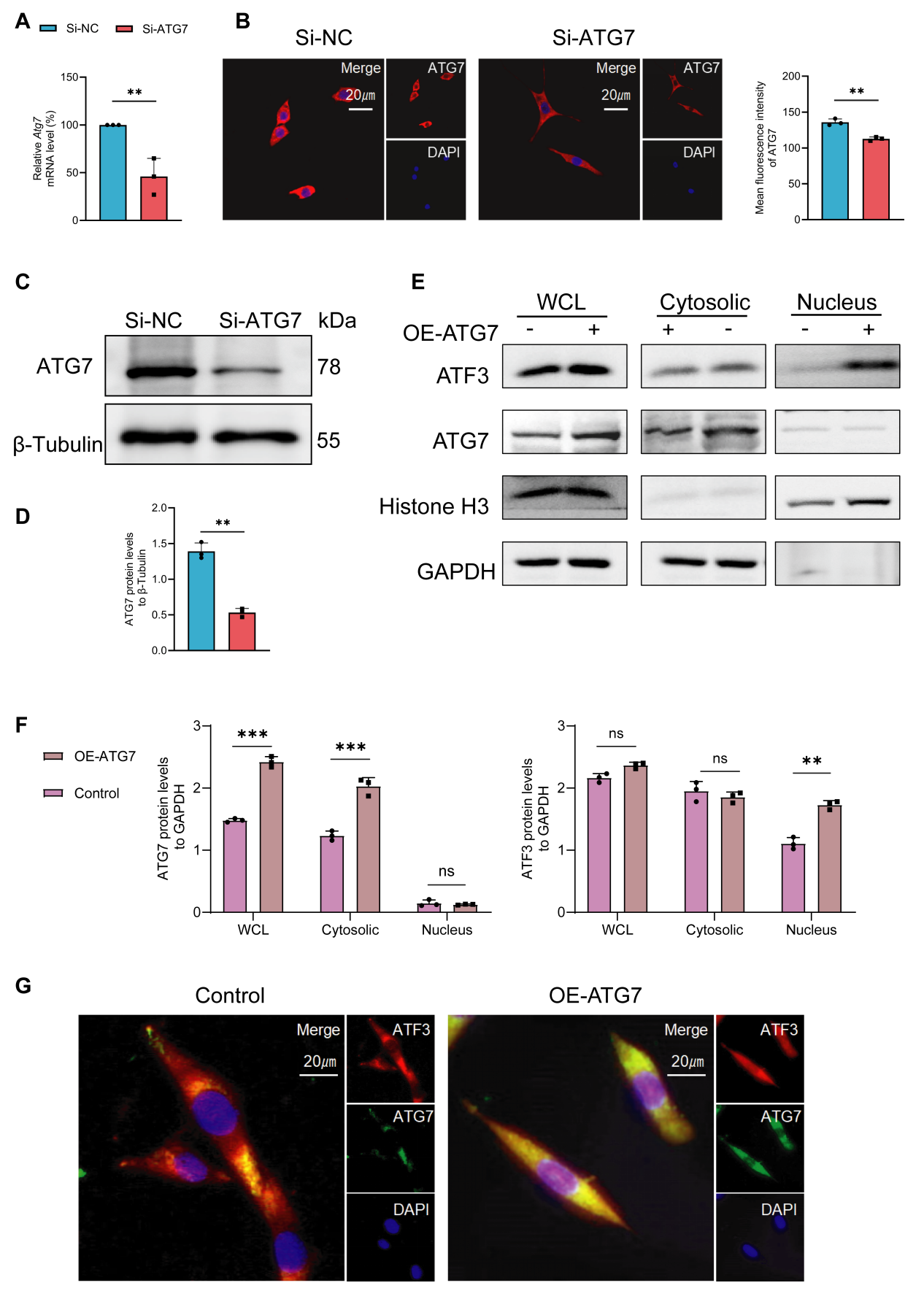
Figure S5


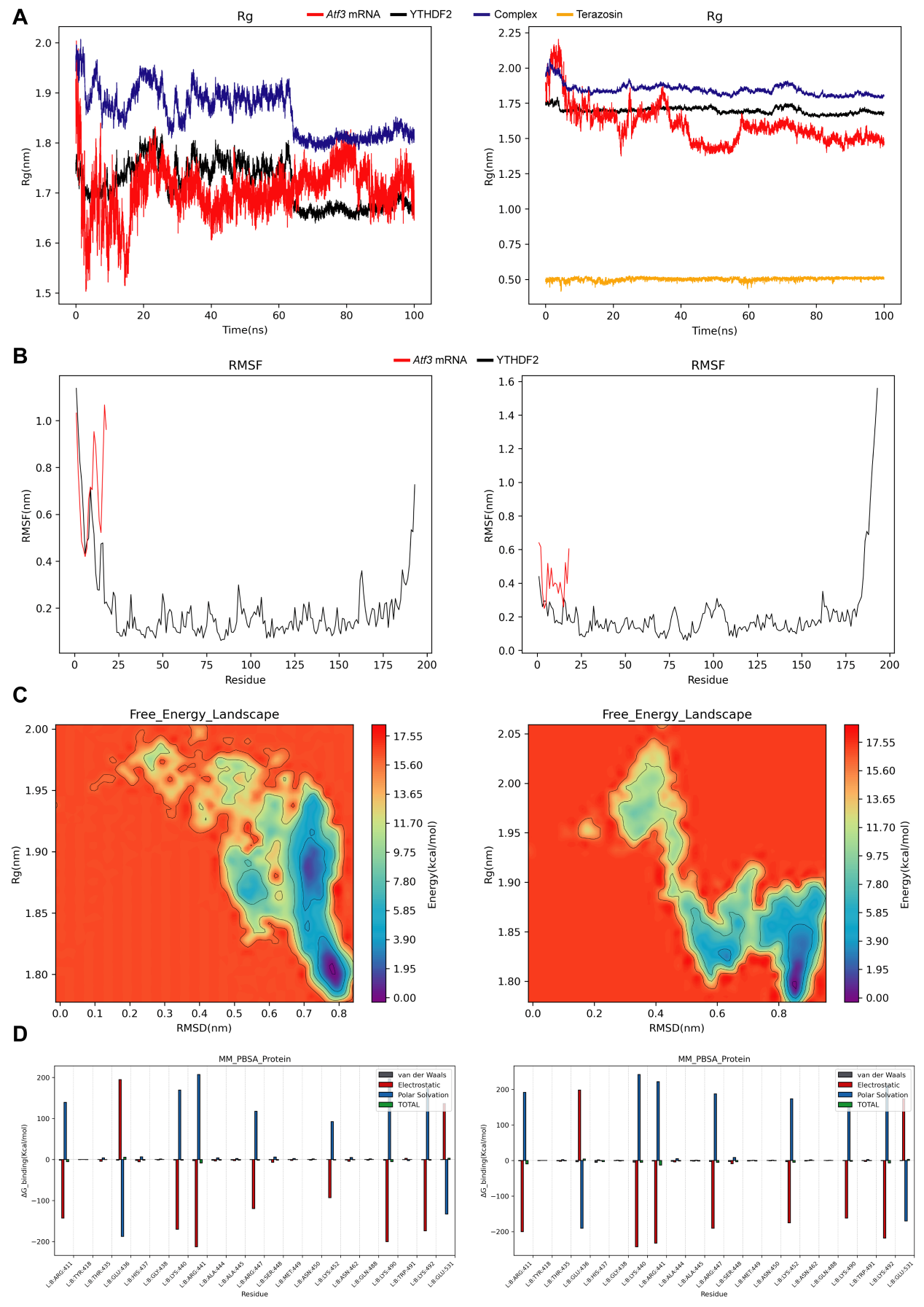
Figure S6


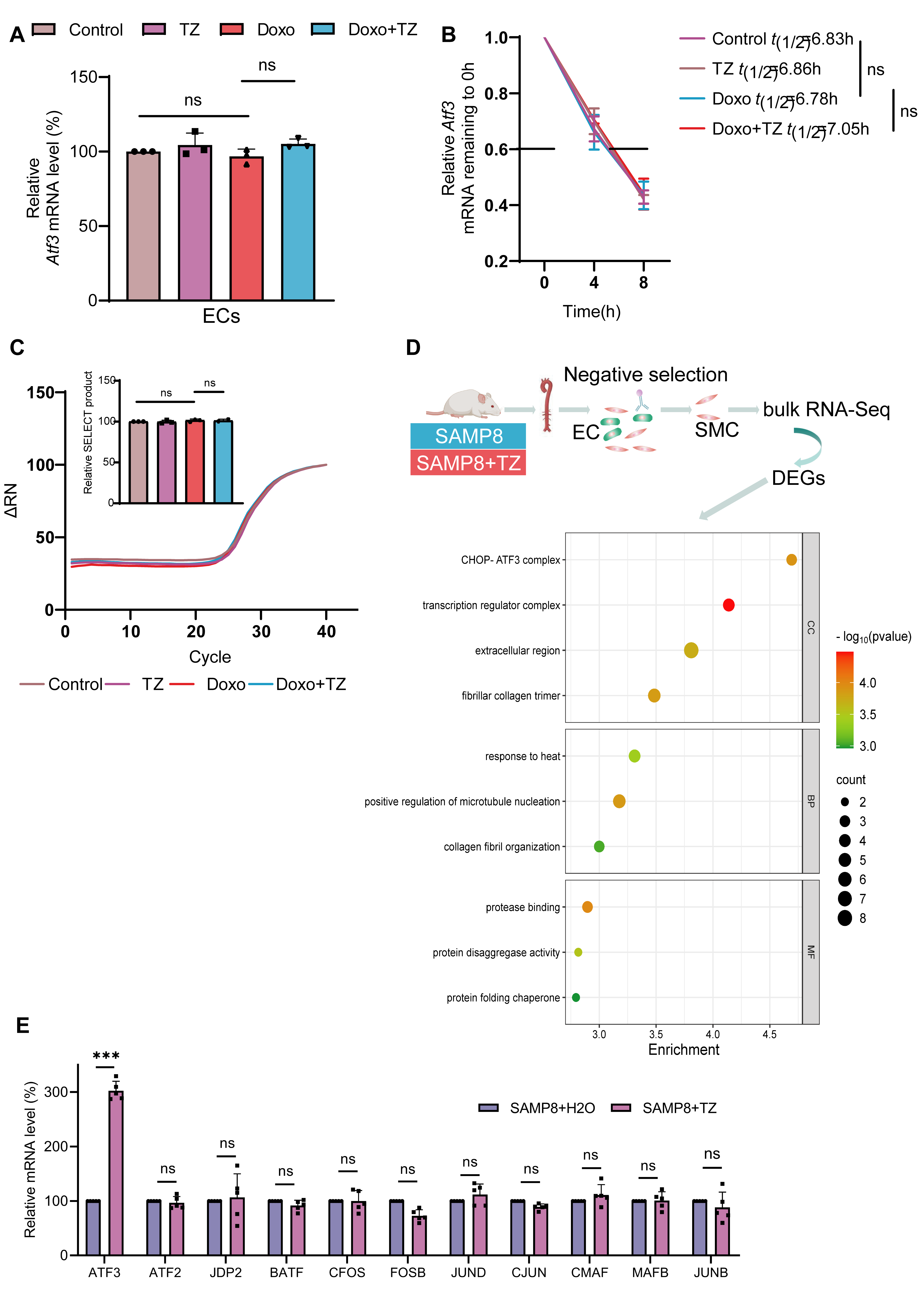
Figure S7


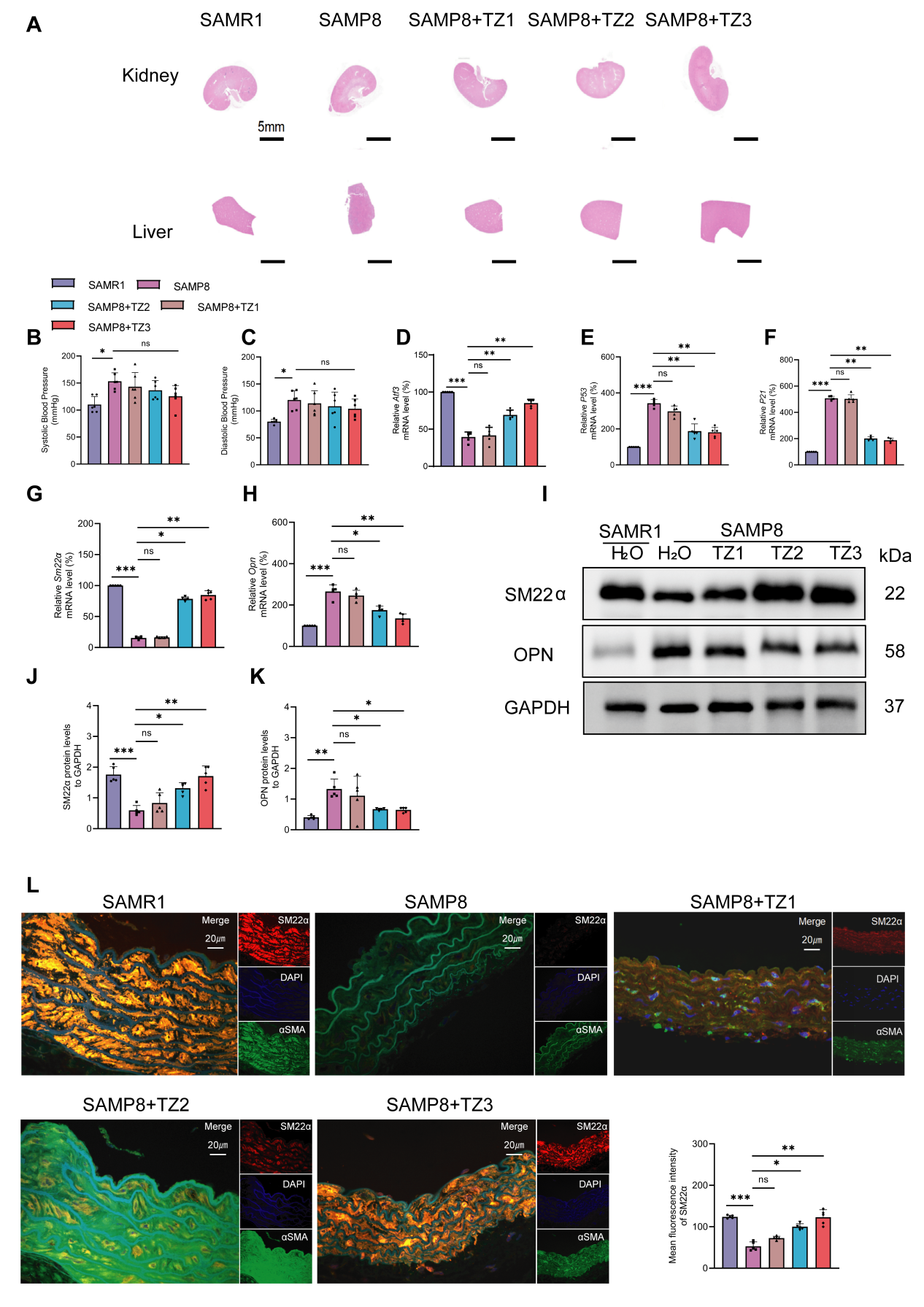
Figure S8


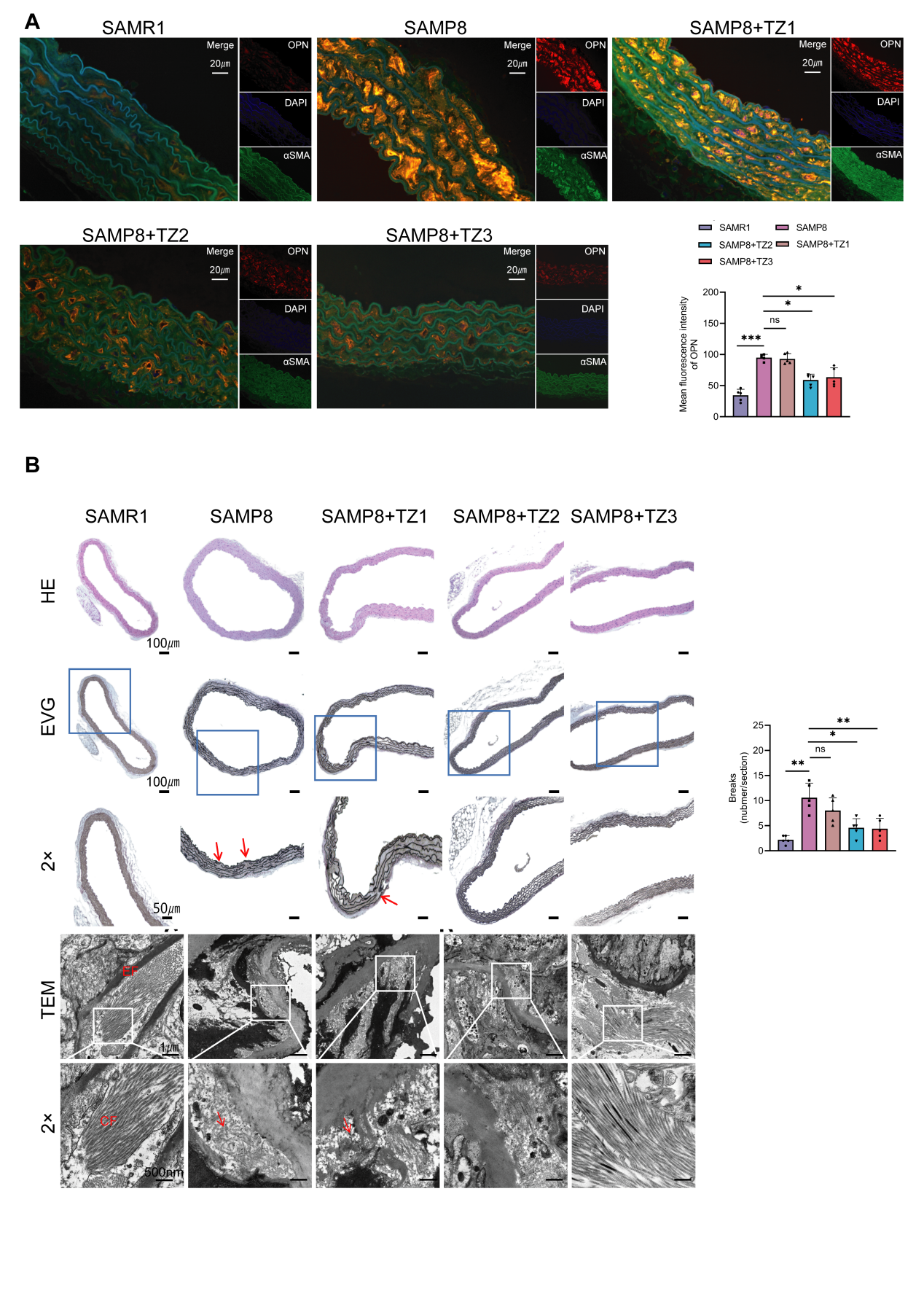
Figure S9


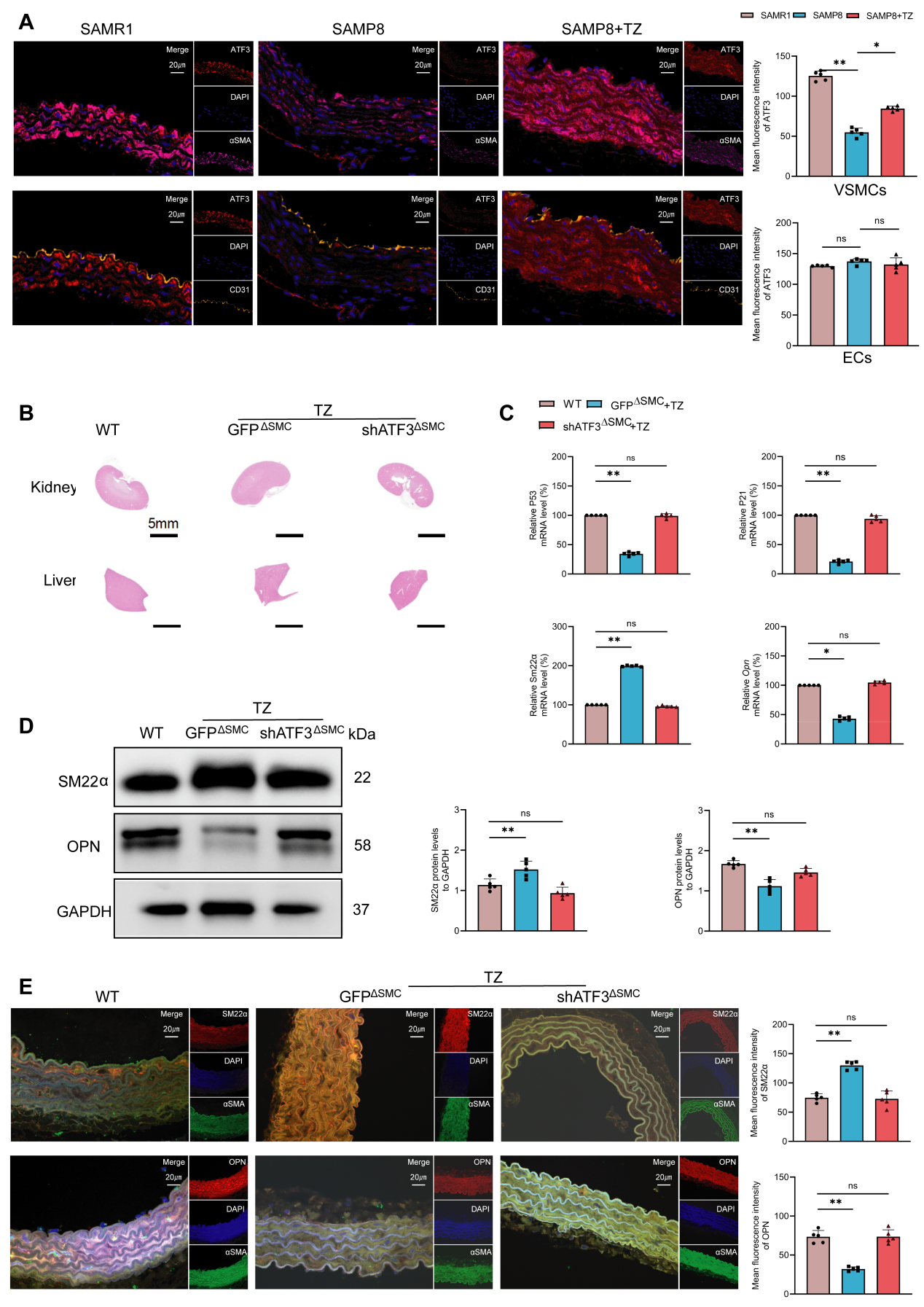
Figure S10


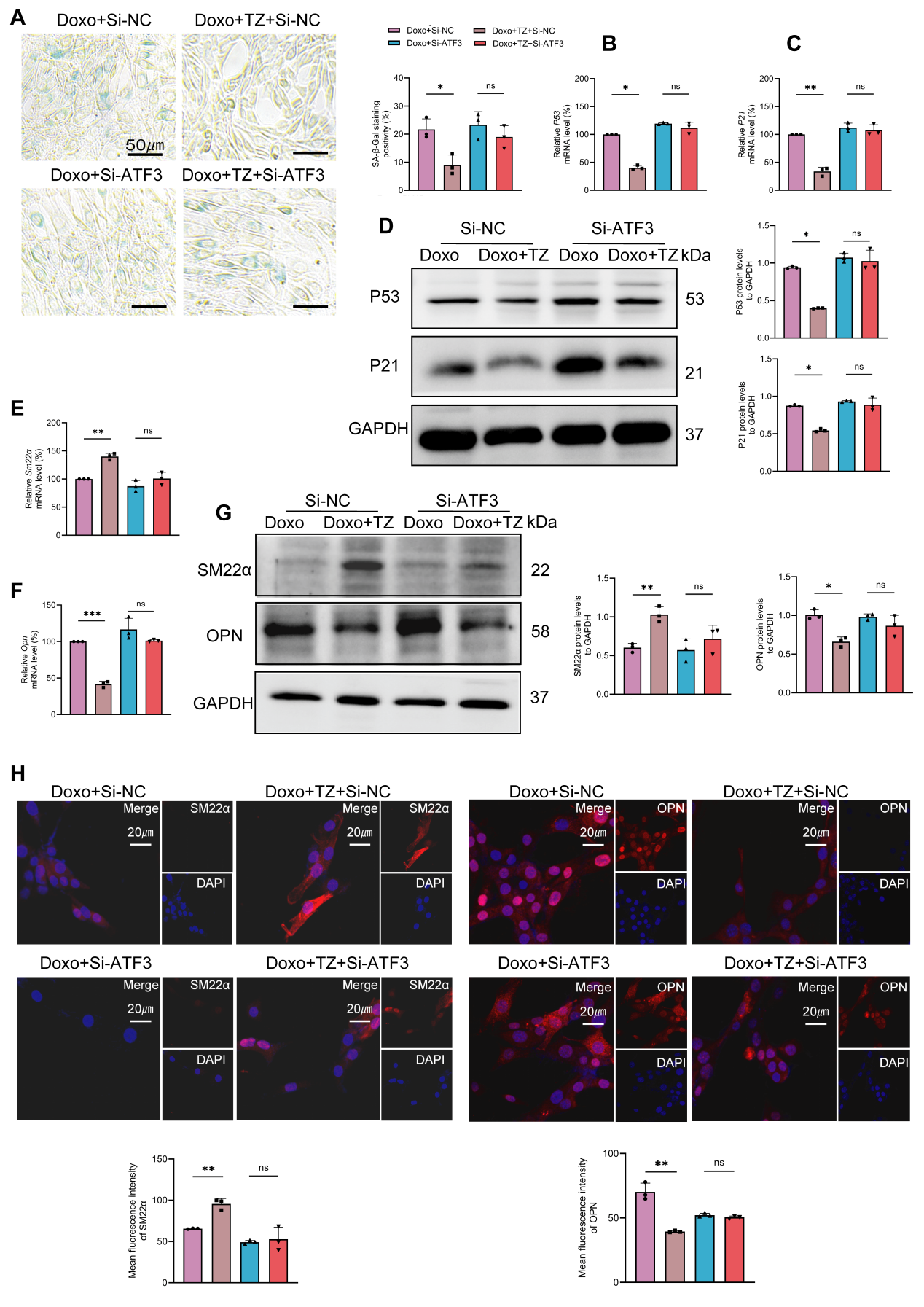
Figure S11


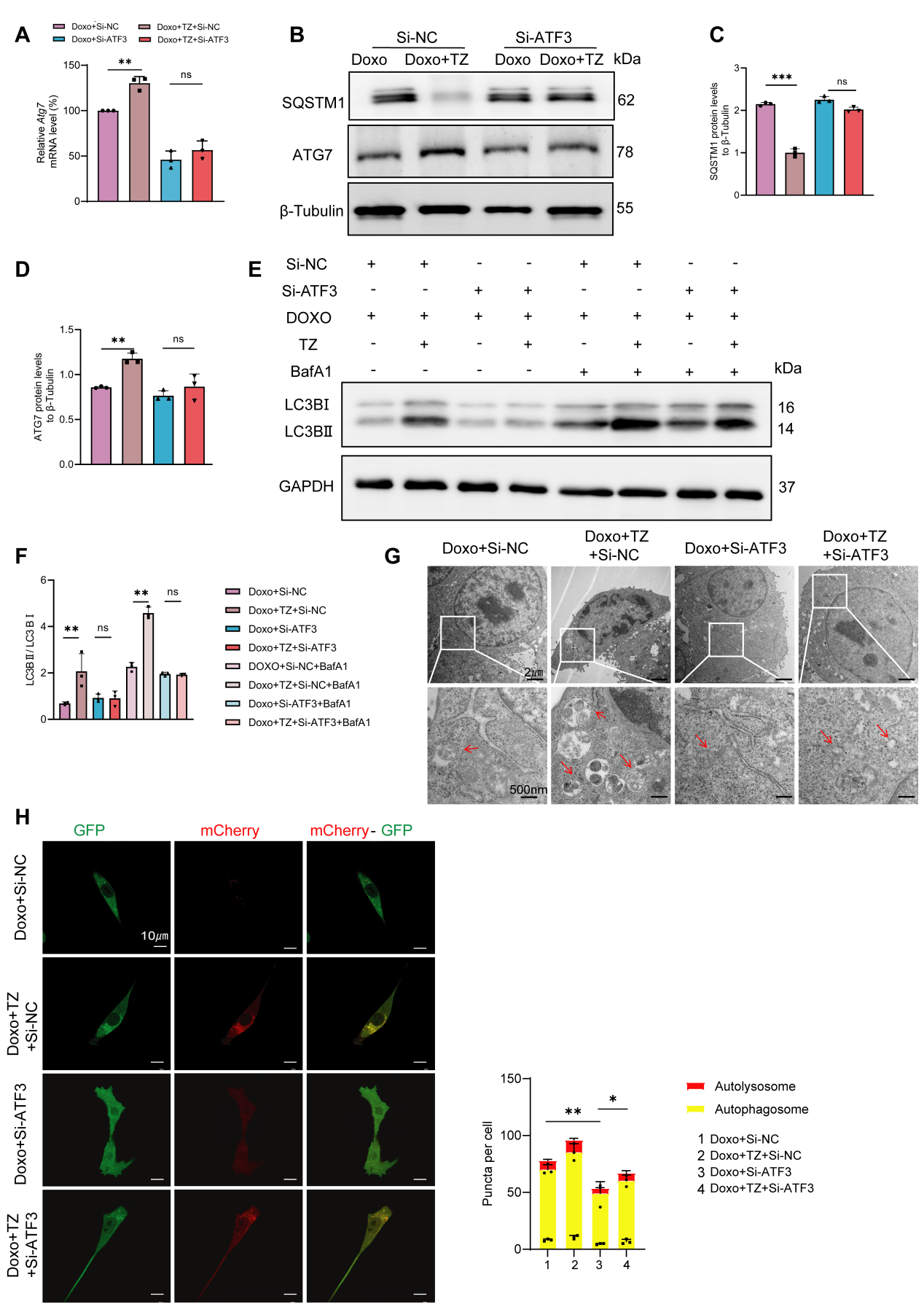
Figure S12


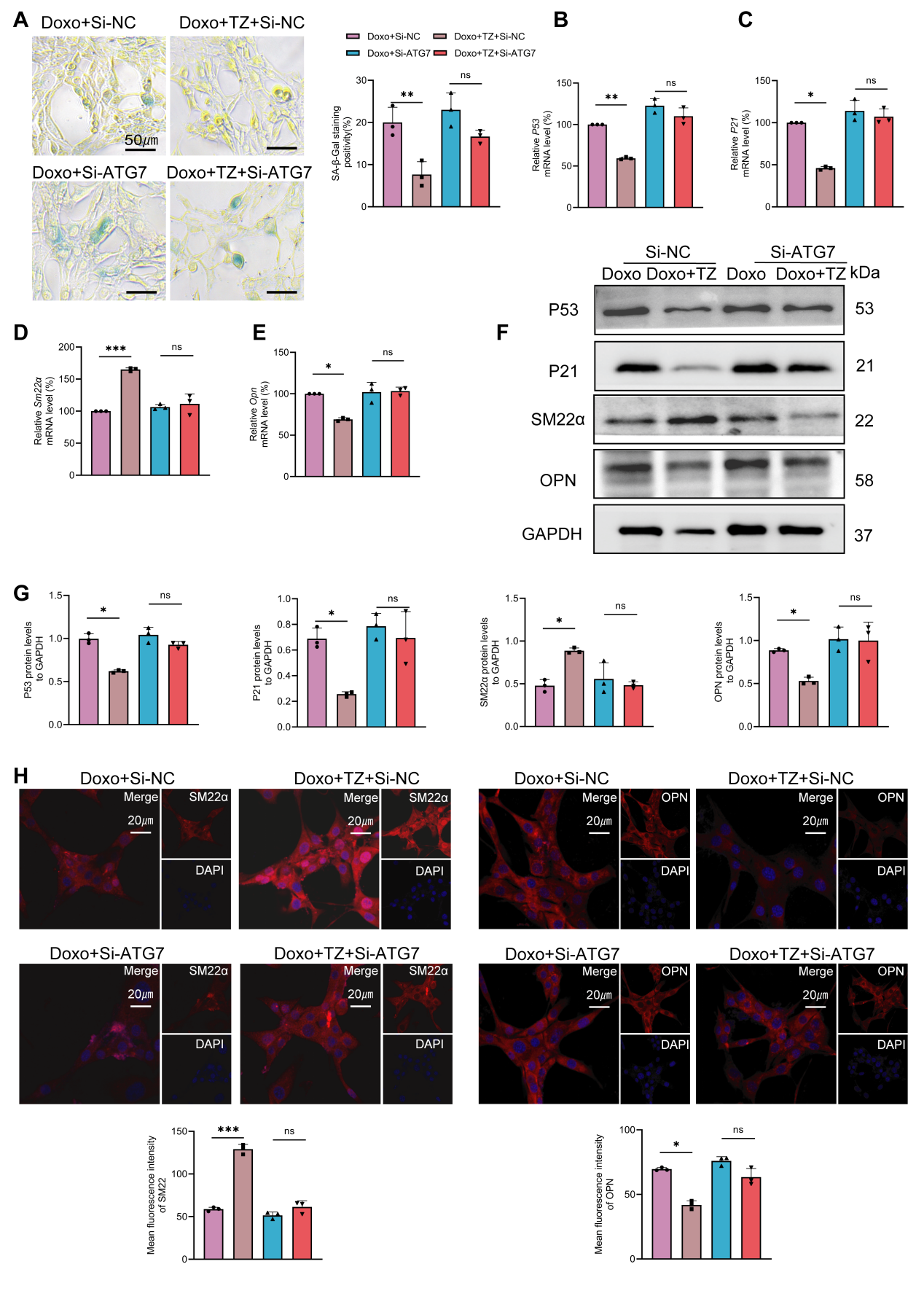
Figure S13


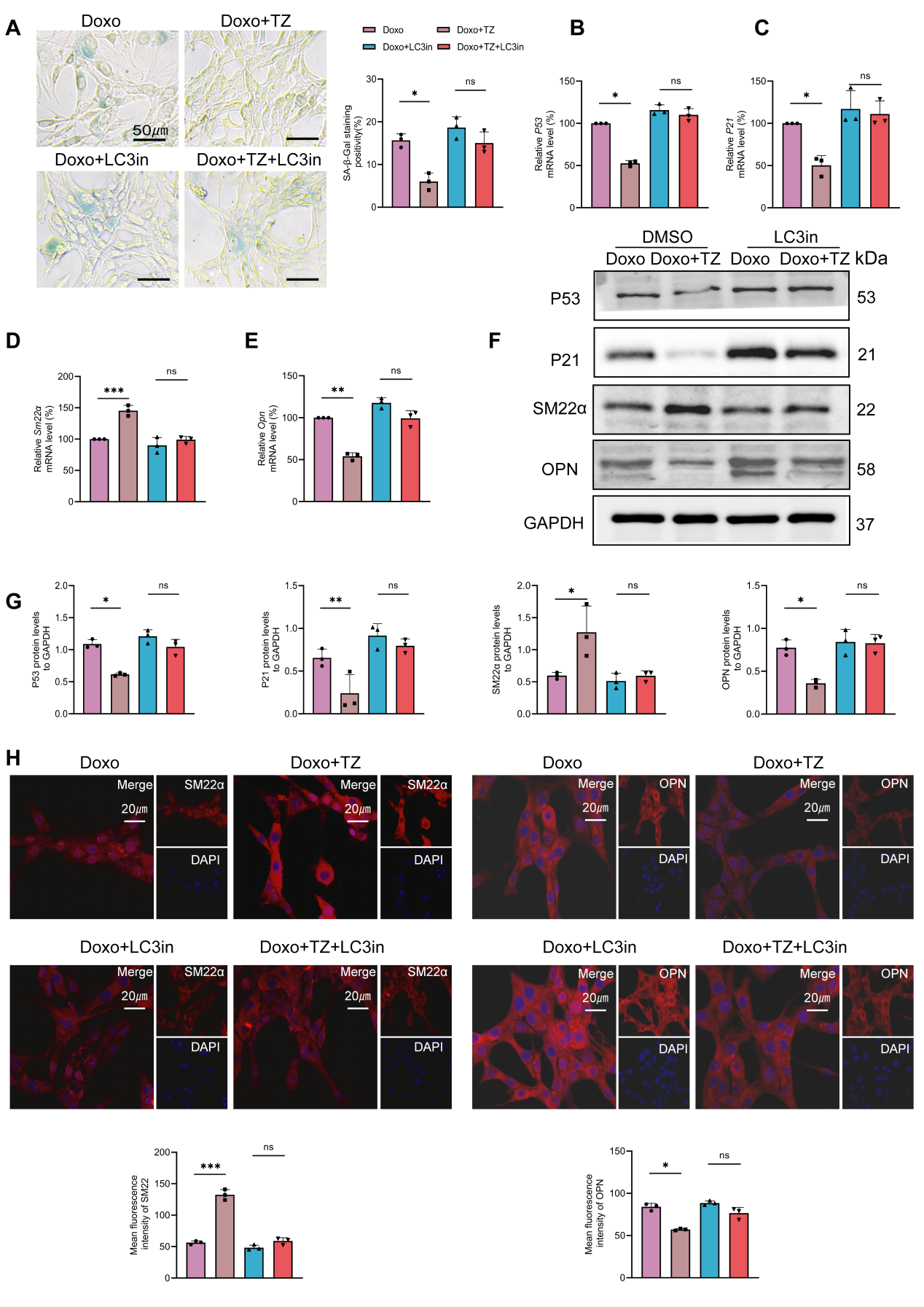
Figure S14


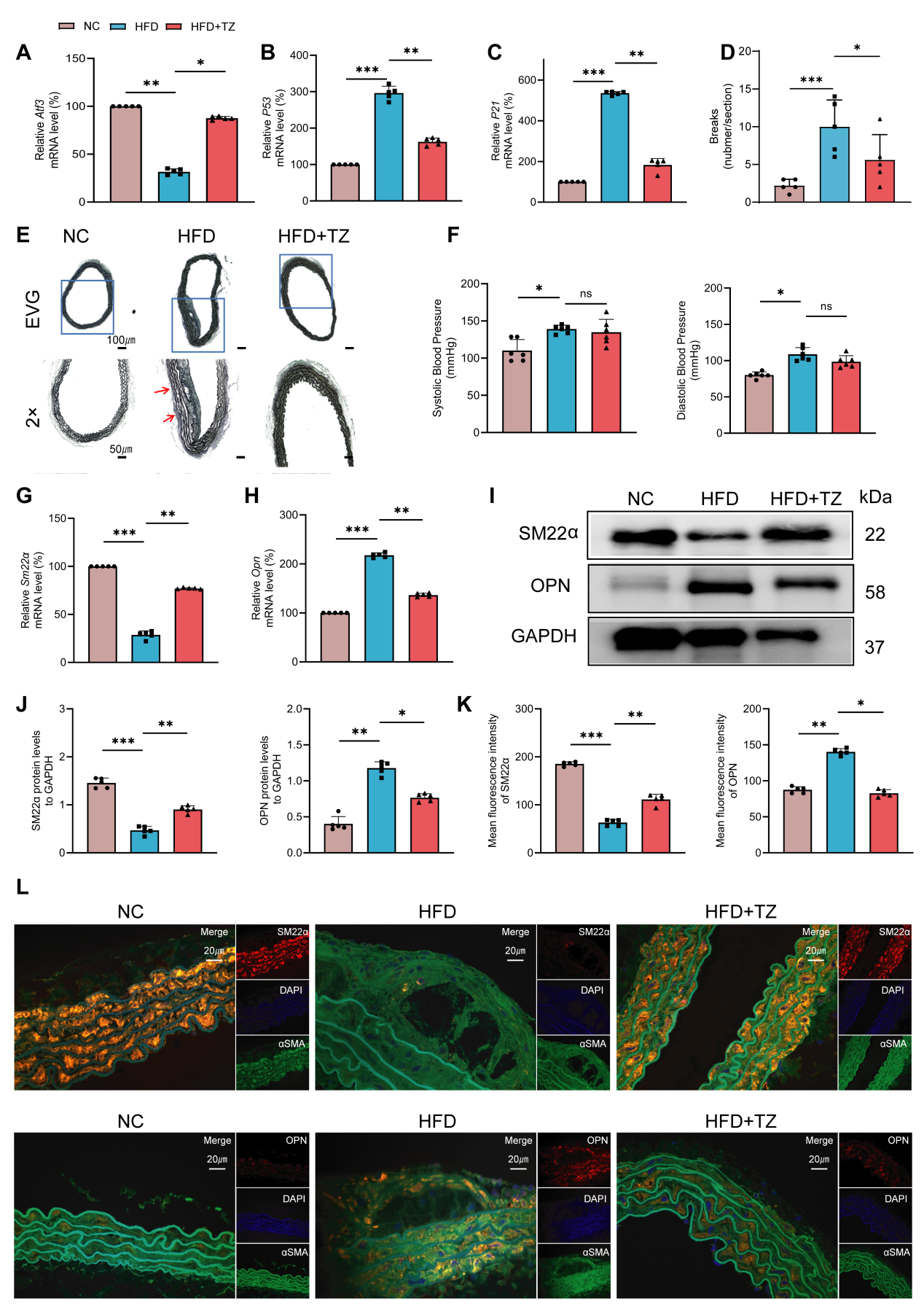
Figure S15


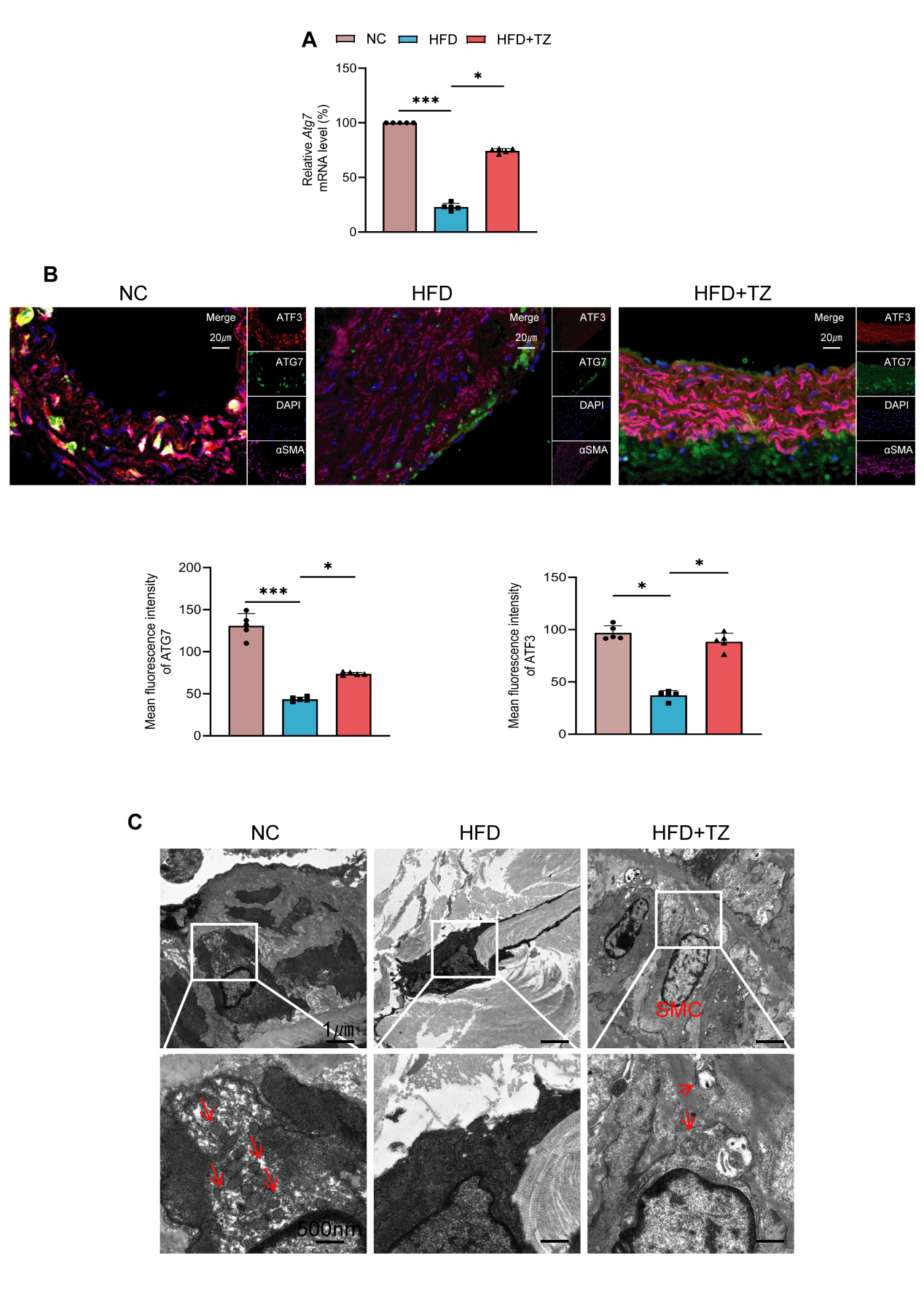
Figure S16
