# Supplementary material for: ATF3 Deficiency Exacerbates Ageing‐Induced Atherosclerosis and Clinical Intervention Strategy
Source: Adv Sci (Weinh). 2025 Jul 11;12(37):e02249. doi: 10.1002/advs.202502249 (PMC12499416; doi:10.1002/advs.202502249)
Supplement: Supplementary file 2 — Supporting Information [file ADVS-12-e02249-s003.docx]

Table S1. Carotid surgery patient information.

| Nubmer | Age | Gender | Blood pressure (mmHg) |
| --- | --- | --- | --- |
| AS1 | 54 | Female | 138/69 |
| AS2 | 42 | Male | 150/78 |
| AS3 | 36 | Male | 142/72 |
| AS4 | 57 | Female | 165/89 |
| AS5 | 47 | Male | 144/76 |
| Healthy-control1 | 56 | - | - |
| Healthy-control2 | 41 | - | - |
| Healthy-control3 | 38 | - | - |
| Healthy-control4 | 52 | - | - |
| Healthy-control5 | 63 | - | - |

Five carotid artery samples were procured from the sample repository maintained by the Department of Cardiovascular Surgery at Tongji Hospital under the approval code TJ-IRB201912154 from 2020 to 2022. Control samples were acquired from three healthy donors who underwent heart transplantation.

Table S2. 16 proteins enriched in the autophagy pathway

| GeneName | Protein_ID | Protein_Qscore | Abundance | iBAQ | Protein_Mass |
| --- | --- | --- | --- | --- | --- |
| Atg7 | sp\|Q9D906\|ATG7_MOUSE | 21.14857023 | 28346837.44 | 5198734.225 | 77470.00519 |
| Map2k2 | sp\|Q63932\|MP2K2_MOUSE | 20.68683217 | 29825935.63 | 1569786.086 | 44374.02854 |
| Map2k1 | sp\|P31938\|MP2K1_MOUSE | 31.99407384 | 20814984.58 | 1156388.032 | 43446.35305 |
| Rraga | sp\|Q80X95\|RRAGA_MOUSE | 19.27260886 | 11470080.78 | 674710.6342 | 36542.55165 |
| Mapk3 | sp\|Q63844\|MK03_MOUSE | 18.3807214 | 12206491 | 554840.4999 | 43039.11436 |
| Rragc | sp\|Q99K70\|RRAGC_MOUSE | 23.96270719 | 10469497.28 | 523474.8641 | 44092.76694 |
| Sqstm1 | sp\|Q61239\|FNTA_MOUSE | 10.89101609 | 7917264.189 | 439848.0105 | 43985.90965 |
| Rheb | sp\|Q921J2\|RHEB_MOUSE | 9.063874436 | 5139534.506 | 428294.5422 | 20438.54698 |
| Prkcd | sp\|P28867\|KPCD_MOUSE | 13.60873933 | 17890548.22 | 425965.4338 | 77496.81483 |
| Prkaa1 | sp\|Q5EG47\|AAPK1_MOUSE | 19.79775145 | 12538212.36 | 391819.1362 | 63888.54597 |
| Prkacb | sp\|P68181\|KAPCB_MOUSE | 13.68933593 | 6810308.854 | 340515.4427 | 40682.03109 |
| Deptor | sp\|Q570Y9\|DPTOR_MOUSE | 13.48533149 | 6993460.875 | 304063.5163 | 46090.16853 |
| Rras2 | sp\|P62071\|RRAS2_MOUSE | 12.19466991 | 2210276.689 | 147351.7792 | 23384.72319 |
| Rras2 | sp\|P62071\|RRAS2_MOUSE | 12.19466991 | 2210276.689 | 147351.7792 | 23384.72319 |
| Atg3 | sp\|Q9CPX6\|ATG3_MOUSE | 6.427688248 | 2340076.651 | 146254.7907 | 35773.40208 |
| Pik3r4 | sp\|Q8VD65\|PI3R4_MOUSE | 19.12398026 | 7317308.191 | 114332.9405 | 152502.4698 |

Table S3. The binding site between protein ATF3 and protein ATG7.

| Receptor | Ligand | Hydrogen bond  Interaction | Electrostatic interaction |
| --- | --- | --- | --- |
| ATG7 | ATF3 | ASN608-LYS97 | GLU609-ARG93 |
| (Alphafold predicted) | (Alphafold predicted) | ASP605-LYS97 |  |
|  |  | GLN456-PRO6 |  |
|  |  | GLN418-ALA11 |  |
|  |  | HIS24-VAL20 |  |
|  |  | SER588-ARG104 |  |
|  |  | CYS318-SER28 |  |

Table S4. The top ten molecules with the highest binding energy in molecular docking.

| Receptor DB ID | binding energy ( kcal/mol) |
| --- | --- |
| [DB01162](https://www.drugbank.ca/drugs/DB01162) | -8.5 |
| T6S0107 | -8.3 |
| PC6987531 | -8.1 |
| PC12095568 | -8.1 |
| DB00436 | -8 |
| DB14649 | -8 |
| DB00443 | -8 |
| T3S0804 | -7.9 |
| T1166 | -7.9 |
| T2868 | -7.9 |

Table S5. Basic characteristics of SAMR1 and SAMP8 mice.

|  | SAMR1  (n=6) | SAMP8  (n=6) | SAMP8  +TZ1  (n=6) | SAMP8  +TZ2  (n=6) | SAMP8  +TZ3  (n=6) | P value |
| --- | --- | --- | --- | --- | --- | --- |
| Weight (g) |  |  |  |  |  |  |
| 8-month-old | 30.5±1.8 | 29.8±1.8 | 30.6±3.2 | 30.1±2.0 | 30.4±0.7 | 0.389 |
| 9-month-old | 35.2±2.4 | 36.6±1.2 | 36.9±1.7 | 35.6±1.7 | 36.4±0.9 | 0.312 |
| 10-month-old | 38.0±1.1 | 38.4±1.1 | 38.9±2.1 | 39.7±2.2 | 39.0±0.5 | 0.377 |
| FBG (mmol/L) | 2.7±0.5 | 2.8±0.5 | 3.3±0.8 | 3.0±0.8 | 2.7±0.2 | 0.28 |
| TC (mmol/L) | 1.7±0.2 | 1.6±0.7 | 1.5±0.6 | 2.1±0.7 | 1.4±0.4 | 0.465 |
| TG (mmol/L) | 1.3±0.2 | 1.5±1.2 | 0.9±0.3 | 1.3±0.6 | 1.4±0.7 | 0.268 |
| HDL-C (mmol/L) | 1.2±0.1 | 1.1±0.4 | 1.1±0.5 | 1.5±0.4 | 1.0±0.2 | 0.491 |

TC, total cholesterol; TG, triglyceride; HDL-C, high-density lipoprotein cholesterol; and LDL-C, low-density lipoprotein cholesterol; FBG, fasting blood glucose. Data were present as means ± SD or median for normally or non-normally distributed continuous variables and as frequency or percentage for categorical variables. The unpaired Student’s t-test or the Mann-Whitney U test was applied to evaluate statistical significance for continuous variables with or without normal distribution, respectively. Meanwhile, the Chi-square test was used to evaluate the statistics.

Table S6. Basic characteristics of SAMR1 and SAMP8 mice.

|  | WT  (n=6) | GFP-AAV  (n=6) | ATF3-AAV  (n=6) | P value |
| --- | --- | --- | --- | --- |
| Weight (g) |  |  |  |  |
| 8-month-old | 29.8±1.8 | 30.3±4.5 | 29.8±1.8 | 0.211 |
| 9-month-old | 36.6±1.2 | 35.6±1.4 | 35.7±1.1 | 0.307 |
| 10-month-old | 38.4±1.1 | 39.8±2.6 | 38.9±0.4 | 0.289 |
| FBG (mmol/L) | 2.8±0.5 | 2.5±0.5 | 2.7±0.3 | 0.212 |
| TC (mmol/L) | 1.6±0.7 | 1.9±0.7 | 1.6±0.5 | 0.245 |
| TG (mmol/L) | 1.5±1.2 | 1.1±0.5 | 1.2±0.9 | 0.251 |
| HDL-C (mmol/L) | 1.1±0.4 | 0.2±0.1 | 0.1±0.0 | 0.195 |
| SBP (mmHg) | 153.2±15.8 | 143.6±16.3 | 148.2±17.9 | 0.619 |
| DBP (mmHg) | 120.5±17.0 | 116.9±13.8 | 119.2±17 | 0.927 |

TC, total cholesterol; TG, triglyceride; HDL-C, high-density lipoprotein cholesterol; and LDL-C, low-density lipoprotein cholesterol; FBG, fasting blood glucose. SBP, systolic blood pressure; DBP, diastolic blood pressure; Data were present as means ± SD or median for normally or non-normally distributed continuous variables and as frequency or percentage for categorical variables. The unpaired Student’s t-test or the Mann-Whitney U test was applied to evaluate statistical significance for continuous variables with or without normal distribution, respectively. Meanwhile, the Chi-square test was used to evaluate the statistics.

Table S7. Primary antibodies applied in IF, WB, IP and RIP

| Antibody | Catalog number | Manufacturer | Dilution ratio |
| --- | --- | --- | --- |
| Anti-P53 | AF0879 | Affinity, China | WB:1:1000 |
| Anti-P21 | sc-6246 | Santa Cruz, China | WB:1:500 |
| Anti-ATF3 | Ab254268 | Abcam, USA | IF:1:100, WB:1:1000, IP: 1/30 |
| Anti-ATG7 | Ab133528 | Abcam, USA | IF:1:100, WB:1:10000, IP: 1/30 |
| Anti-α-SMA | 19245S | CST, USA | WB:1:5000 |
| Anti-SM22α | 10493-1-AP | Proteintech, China | IF:1:200, WB:1:5000 |
| Anti-OPN | 22952-1-AP | Proteintech, China | IF:1:20, WB:1:1000 |
| Anti-LC3 | 4108S | CST, USA | WB:1:1000 |
| Anti-GAPDH | 10494-1-AP | Proteintech, China | WB:1:10000 |
| Anti-β-Tubulin | ABL1030, | Abbkine, China | WB:1:10000 |
| Anti-Histone H3 | 180531 | Servicebio, China | WB:1:1000 |
| Anti-Flag | 66008-4-Ig | Proteintech, China | WB:1:5000, IP: 1/30 |
| Anti-HA | 66006-2-Ig | Proteintech, China | WB:1:10000, IP: 1/30 |
| Anti-His | 66005-1-Ig | Proteintech, China | WB:1:1000 |
| Anti-GST | 66001-2-Ig | Proteintech, China | WB:1:5000 |
| Anti-YTHDF2 | Ab220163 | Abcam, USA | RIP: 1/30 |

Table S8. Primers applied in qRT-PCR

| Gene name | Species | Forward (5'-3') | Reverse (5'-3') |
| --- | --- | --- | --- |
| *P53* | Mouse | TGGAAGGAAATTTGTATCCCGA | GTGGATGGTGGTATACTCAGAG |
| *P21* | Mouse | CCTGGTGATGTCCGACCTG | CCATGAGCGCATCGCAATC |
| *Atf3* | Mouse | CACCTCCTGGGTCACTGGTATTTG | TGGCGAATCTCAGCTCTTCCTTG |
| *Atg7* | Mouse | GGTGAACCTCAGTGGATGTATGGAC | CCAGCAGCAGGCACTTGACAG |
| *Acta2* | Mouse | GTCCCAGACATCAGGGAGTAA | TCGGATACTTCAGCGTCAGGA |
| *Tagln* | Mouse | CAACAAGGGTCCATCCTACGG | ATCTGGGCGGCCTACATCA |
| *Opn* | Mouse | AGCAAGAAACTCTTCCAAGCAA | GTGAGATTCGTCAGATTCATCCG |
| *18S* | Mouse | CTCAACACGGGAAACCTCAC | CGCTCCACCAACTAAGAACG |
| *Atg7-promoter* | Mouse | GGGAAATTTCCCGGGAAATT | TTTCCCGGGAAATTTCCCGG |
| *Vcam-1* | Mouse | CTGGGAAGCTGGAAGAAATGC | CGGTTTGGGGTACTCTCTGG |
| *Il-6* | Mouse | CTGGGAAGCTGGAAGAAATGC | CGGTTTGGGGTACTCTCTGG |
| *Tnf* | Mouse | CAGGCGGTGCCTATGTCTC | CGATCACCCCGAAGTTCAGTAG |

Table S9. Wild-type and mutation sequence of ATF3 applied in Co-IP

| Name | Sequence |
| --- | --- |
| ATF3-WT | Atgatgcttcaacatccaggccaggtctctgcctcagaagtcagtgcgaccgccattgtcccctgcctctcacctcctgggtcactggtatttgaggattttgctaacctgacaccctttgtcaaggaagagctgagattcgccatccagaataaacacctctgccatcggatgtcctctgcgctggagtcagttaccgtcaacaacagacccctggagatgtcagtcaccaagtctgaggcggcccctgaagaagatgagaggaaaaggaggcggcgagaaagaaataaaattgctgctgccaagtgtcgaaacaagaaaaaggagaagacagagtgcctgcagaaagagtcagagaaactggagagtgtgaatgctgagctgaaggcccagattgaggagctgaagaatgagaaacagcatttgatatacatgctcaacctgcaccggcccacctgcatcgtccgggctcagaatggacggacaccggaagacgagaggaacctctttatccaacagataaaagaaggaacattgcagagc |
| ATF3-MUT1(ARG93GLU) | Atgatgcttcaacatccaggccaggtctctgcctcagaagtcagtgcgaccgccattgtcccctgcctctcacctcctgggtcactggtatttgaggattttgctaacctgacaccctttgtcaaggaagagctgagattcgccatccagaataaacacctctgccatcggatgtcctctgcgctggagtcagttaccgtcaacaacagacccctggagatgtcagtcaccaagtctgaggcggcccctgaagaagatgagaggaaaaggaggcgggaggaaagaaataaaattgctgctgccaagtgtcgaaacaagaaaaaggagaagacagagtgcctgcagaaagagtcagagaaactggagagtgtgaatgctgagctgaaggcccagattgaggagctgaagaatgagaaacagcatttgatatacatgctcaacctgcaccggcccacctgcatcgtccgggctcagaatggacggacaccggaagacgagaggaacctctttatccaacagataaaagaaggaacattgcagagc |
| ATF3-MUT2(LYS97GLU) | Atgatgcttcaacatccaggccaggtctctgcctcagaagtcagtgcgaccgccattgtcccctgcctctcacctcctgggtcactggtatttgaggattttgctaacctgacaccctttgtcaaggaagagctgagattcgccatccagaataaacacctctgccatcggatgtcctctgcgctggagtcagttaccgtcaacaacagacccctggagatgtcagtcaccaagtctgaggcggcccctgaagaagatgagaggaaaaggaggcggcgagaaagaaatgagattgctgctgccaagtgtcgaaacaagaaaaaggagaagacagagtgcctgcagaaagagtcagagaaactggagagtgtgaatgctgagctgaaggcccagattgaggagctgaagaatgagaaacagcatttgatatacatgctcaacctgcaccggcccacctgcatcgtccgggctcagaatggacggacaccggaagacgagaggaacctctttatccaacagataaaagaaggaacattgcagagc |
| ATF3-MUT3(ARG104GLU) | Atgatgcttcaacatccaggccaggtctctgcctcagaagtcagtgcgaccgccattgtcccctgcctctcacctcctgggtcactggtatttgaggattttgctaacctgacaccctttgtcaaggaagagctgagattcgccatccagaataaacacctctgccatcggatgtcctctgcgctggagtcagttaccgtcaacaacagacccctggagatgtcagtcaccaagtctgaggcggcccctgaagaagatgagaggaaaaggaggcggcgagaaagaaataaaattgctgctgccaagtgtgagaacaagaaaaaggagaagacagagtgcctgcagaaagagtcagagaaactggagagtgtgaatgctgagctgaaggcccagattgaggagctgaagaatgagaaacagcatttgatatacatgctcaacctgcaccggcccacctgcatcgtccgggctcagaatggacggacaccggaagacgagaggaacctctttatccaacagataaaagaaggaacattgcagagc |

Table S10. Wild-type and mutation sequence of *Atg7* promoter applied in Dual-Luciferase Reporter Assay

| Name | Sequence |
| --- | --- |
| *Atg7* promoter (-1963to+37)WT | TTGAACCTAGGCTCTAGGTCTTGCAGAGTCCCGTGCATAACCTCAGTGTAAGGATATCTAGTTCTACAGCTTTAGGCCAATGTAGCATTCCAAGTCCCCATCCTAAAAAGAATGATCACAGTGTCACTTCAATGTAGCCTACTCTGTGCATACCACTGAAAATATTGAGAAGAGTGTGTGGGGATAGATAAATTTTCATTTGTCCCAAGGTTAGCTTCTCCCATCAAAGAAGCCACCAGAGAACAGGCTGGCCAGGAGAGGGAACAGTCCATCCAGCACAGCATGGTCCAGCCCTCTTGTATTATACTAGCTCCCATTTGCCTTTTTTTTTCTGGTCTTGTGTGTTACTCCTTAAGCAGAGAAAACTTATCAGAAGCAGGAATTCACACCTTCCATCTTGCTCTCTGTGATGTTTGGGGCAAGATGATGACTTGGGTGCAAAATTGTTGATCAGCTGTGATTGGTAGCCAGTTGTGGTCCTGGAGACATTTACCCACCTGTAGTTCAGGGTGGTCTTCCATTCCAGTAGTTATTCCTTGTTCCCCAACTTCATGCCTTCTTCCTAACGCTCATTTTGTGGGGTTTTTTTTCTTATTTTTCAGAGTCTAAGATTATTTCCTGTCACTCTGACTCCAAAGTGTCTGTCTCTCTCTCTCTCTGTCTGTCTCTCTTTCTTTTTTCCAGGAACCACTTTCCATTTGTTTACTCTGCCCCCCCCCCCGTATAAGCTATAAGGCACACACACACACACACACACACACACACACACACACACACACACACCACCTTGTAATTTATCCATTAGAAATAGGAAACCCTTAATGTAAAAAAAAATCAATAAATAGTCCAAGATATTATGATGAATCCCTAGAGGCACTCCAAAGGCACTGTGTGCCAGGAAAGGTGTCCGTGGACTGCCATCTCAGCAATGGTGATGCGGTATCAGGATGCTTGCCTTGAGCTCTGATCATCCTGGACTATAGAACTAAATCAAGTCAAGTACACTCTTGTCGCTAAGGAAGTCACTCATGTGCAAGACTCCCCAGGCTAGAGCATTTCATTATGTTCATTATCCTGTCAAAAAAAAAAATGTTTTCCCAGTAGTAAGTTTTTTGTTCGTTTTATTTGTTTGTTTGTTTTGTTTGAGATAGTGTAGCCCTGGCTGTCCCAGAACTCACTACTCACAGATCCACCTACCTCTGCCTCCCAAATGCTGGATTTTTTGTTTTGTTTTGTTTTGTTTTGTTTTTCGAGACAGGGTTTCTCTGTATAACTCTGGCTGTCCTGGAACTCACTTTGTAGACCAGGCTGGCCTTGAACTCAGAAATCTGCCTGCCTCTGCCTCCTGAGGGCTGGGATTAAAATCTTGTGCCACCACGCCCAGCCCAAATGCTGGAATTTAAGACATATCCCACACCGTCTGGCCCATATTAGGTTCTTAAGAAGTGATTTTATAGTCACTGAAGTTGCTGGCTGCAGTAGCTTGGTGGATATTTTAGATTCTATTTGGTTTTCCAGAGTTATATTTGGTTGGGCAAGCTTTTAATCCCAGCACTGGGGCAAAGCAAAGGTAAGCAATCTCTGTTAGTTCGAGACTAGACTGATGTATATGTATACAGGTCCAGAACAGCCATGGCTATGTAGTGTAAATATGAGGAGGACATAACCTTTTTAAAAAATTAAAAATGCAAAAAATAAAAATAAACCTTGTTTCAACACCTGTACTCTAGTACTACCCTGGGAATTTGATAATAATTCCGTAGTACTGTAGAGTCACTCGCAGGAAAATGTAATCTTCCTTACAAGCCCCAAAAGGCTTACAGGTTAGCCTTAGTTTCGCCCCGTTATTGCGGTTGTGGGGACCCTTTTTGTCGTTGTTGTTGTAGTGCGCATGCGCGCCGCGCTTCCGCGTTTGTGTGGGCTGGGTTATTACGTCATTGGGCCGCGCGGCGCGGCTGGGGCTGTGGTTGCCGGAAGTTGAGCGGCGGTAAGTAAGCCGT |
| *Atg7* promoter (-1963to+37)MT1 | TTGAACCTAGGCTCTAGGTCTTGCAGAGTCCCGTGCATAACCTCAGTGTAAGGATATCTAGTTCTACAGCTTTAGGCCAATGTAGCATTCCAAGTCCCCATCCTAAAAAGAATGATCACAGTGTCACTTCAATGTAGCCTACTCTGTGCATACCACTGAAAATATTGAGAAGAGTGTGTGGGGATAGATAAATTTTCATTTGTCCCAAGGTTAGCTTCTCCCATCAAAGAAGCCACCAGAGAACAGGCTGGCCAGGAGAGGGAACAGTCCATCCAGCACAGCATGGTCCAGCCCTCTTGTATTATACTAGCTCCCATTTGCCTTTTTTTTTCTGGTCTTGTGTGTTACTCCTTAAGCAGAGAAAACTTATCAGAAGCAGGAATTCACACCTTCCATCTTGCTCTCTGTGATGTTTGGGGCAAGATTCGTCAGGGGGTGCAAAATTGTTGATCAGCTGTGATTGGTAGCCAGTTGTGGTCCTGGAGACATTTACCCACCTGTAGTTCAGGGTGGTCTTCCATTCCAGTAGTTATTCCTTGTTCCCCAACTTCATGCCTTCTTCCTAACGCTCATTTTGTGGGGTTTTTTTTCTTATTTTTCAGAGTCTAAGATTATTTCCTGTCACTCTGACTCCAAAGTGTCTGTCTCTCTCTCTCTCTGTCTGTCTCTCTTTCTTTTTTCCAGGAACCACTTTCCATTTGTTTACTCTGCCCCCCCCCCCGTATAAGCTATAAGGCACACACACACACACACACACACACACACACACACACACACACACACCACCTTGTAATTTATCCATTAGAAATAGGAAACCCTTAATGTAAAAAAAAATCAATAAATAGTCCAAGATATTATGATGAATCCCTAGAGGCACTCCAAAGGCACTGTGTGCCAGGAAAGGTGTCCGTGGACTGCCATCTCAGCAATGGTGATGCGGTATCAGGATGCTTGCCTTGAGCTCTGATCATCCTGGACTATAGAACTAAATCAAGTCAAGTACACTCTTGTCGCTAAGGAAGTCACTCATGTGCAAGACTCCCCAGGCTAGAGCATTTCATTATGTTCATTATCCTGTCAAAAAAAAAAATGTTTTCCCAGTAGTAAGTTTTTTGTTCGTTTTATTTGTTTGTTTGTTTTGTTTGAGATAGTGTAGCCCTGGCTGTCCCAGAACTCACTACTCACAGATCCACCTACCTCTGCCTCCCAAATGCTGGATTTTTTGTTTTGTTTTGTTTTGTTTTGTTTTTCGAGACAGGGTTTCTCTGTATAACTCTGGCTGTCCTGGAACTCACTTTGTAGACCAGGCTGGCCTTGAACTCAGAAATCTGCCTGCCTCTGCCTCCTGAGGGCTGGGATTAAAATCTTGTGCCACCACGCCCAGCCCAAATGCTGGAATTTAAGACATATCCCACACCGTCTGGCCCATATTAGGTTCTTAAGAAGTGATTTTATAGTCACTGAAGTTGCTGGCTGCAGTAGCTTGGTGGATATTTTAGATTCTATTTGGTTTTCCAGAGTTATATTTGGTTGGGCAAGCTTTTAATCCCAGCACTGGGGCAAAGCAAAGGTAAGCAATCTCTGTTAGTTCGAGACTAGACTGATGTATATGTATACAGGTCCAGAACAGCCATGGCTATGTAGTGTAAATATGAGGAGGACATAACCTTTTTAAAAAATTAAAAATGCAAAAAATAAAAATAAACCTTGTTTCAACACCTGTACTCTAGTACTACCCTGGGAATTTGATAATAATTCCGTAGTACTGTAGAGTCACTCGCAGGAAAATGTAATCTTCCTTACAAGCCCCAAAAGGCTTACAGGTTAGCCTTAGTTTCGCCCCGTTATTGCGGTTGTGGGGACCCTTTTTGTCGTTGTTGTTGTAGTGCGCATGCGCGCCGCGCTTCCGCGTTTGTGTGGGCTGGGTTATTACGTCATTGGGCCGCGCGGCGCGGCTGGGGCTGTGGTTGCCGGAAGTTGAGCGGCGGTAAGTAAGCCGT |
| *Atg7* promoter (-1963to+37)MT2 | TTGAACCTAGGCTCTAGGTCTTGCAGAGTCCCGTGCATAACCTCAGTGTAAGGATATCTAGTTCTACAGCTTTAGGCCAATGTAGCATTCCAAGTCCCCATCCTAAAAAGAATGATCACAGTGTCACTTCAATGTAGCCTACTCTGTGCATACCACTGAAAATATTGAGAAGAGTGTGTGGGGATAGATAAATTTTCATTTGTCCCAAGGTTAGCTTCTCCCATCAAAGAAGCCACCAGAGAACAGGCTGGCCAGGAGAGGGAACAGTCCATCCAGCACAGCATGGTCCAGCCCTCTTGTATTATACTAGCTCCCATTTGCCTTTTTTTTTCTGGTCTTGTGTGTTACTCCTTAAGCAGAGAAAACTTATCAGAAGCAGGAATTCACACCTTCCATCTTGCTCTCTGTGATGTTTGGGGCAAGATGATGACTTGGGTGCAAAATTGTTGATCAGCTGTGATTGGTAGCCAGTTGTGGTCCTGGAGACATTTACCCACCTGTAGTTCAGGGTGGTCTTCCATTCCAGTAGTTATTCCTTGTTCCCCAACTTCATGCCTTCTTCCTAACGCTCATTTTGTGGGGTTTTTTTTCTTATTTTTCAGAGTCTAAGATTATTTCCTGTCACTCTGACTCCAAAGTGTCTGTCTCTCTCTCTCTCTGTCTGTCTCTCTTTCTTTTTTCCAGGAACCACTTTCCATTTGTTTACTCTGCCCCCCCCCCCGTATAAGCTATAAGGCACACACACACACACACACACACACACACACACACACACACACACACCACCTTGTAATTTATCCATTAGAAATAGGAAACCCTTAATGTAAAAAAAAATCAATAAATAGTCCAAGATATTATGATGAATCCCTAGAGGCACTCCAAAGGCACTGTGTGCCAGGAAAGGTGTCCGTGGACTGCCATCTCAGCAATGGTGATGCGGTATCAGGATGCTTGCCTTGAGCTCTGATCATCCTGGACTATAGAACTAAATCAAGTCAAGTACACTCTTGTCGCTAAGGAAGTCACTCATGTGCAAGACTCCCCAGGCTAGAGCATTTCATTATGTTCATTATCCTGTCAAAAAAAAAAATGTTTTCCCAGTAGTAAGTTTTTTGTTCGTTTTATTTGTTTGTTTGTTTTGTTTGAGATAGTGTAGCCCTGGCTGTCCCAGAACTCACTACTCACAGATCCACCTACCTCTGCCTCCCAAATGCTGGATTTTTTGTTTTGTTTTGTTTTGTTTTGTTTTTCGAGACAGGGTTTCTCTGTATAACTCTGGCTGTCCTGGAACTCACTTTGTAGACCAGGCTGGCCTTGAACTCAGAAATCTGCCTGCCTCTGCCTCCTGAGGGCTGGGATTAAAATCTTGTGCCACCACGCCCAGCCCAAATGCTGGAATTTAAGACATATCCCACACCGTCTGGCCCATATTAGGTTCTTAAGAAGTGATTTTATAGTCACTGAAGTTGCTGGCTGCAGTAGCTTGGTGGATATTTTAGATTCTATTTGGTTTTCCAGAGTTATATTTGGTTGGGCAAGCTTTTAATCCCAGCACTGGGGCAAAGCAAAGGTAAGCAATCTCTGTTAGTTCGAGACTAGACTGATGTATATGTATACAGGTCCAGAACAGCCATGGCTATGTAGTGTAAATATGAGGAGGACATAACCTTTTTAAAAAATTAAAAATGCAAAAAATAAAAATAAACCTTGTTTCAACACCTGTACTCTAGTACTACCCTGGGAATTTGATAATAATTCCGTAGTACTGTAGAGTCACTCGCAGGAAAATGTAATCTTCCTTACAAGCCCCAAAAGGCTTACAGGTTAGCCTTAGTTTCGCCCCGTTATTGCGGTTGTGGGGACCCTTTTTGTCGTTGTTGTTGTAGTGCGCATGCGCGCCGCGCTTCCGCGTTTGTGTGGGCTGGGTTATTCATGACGGGGGCCGCGCGGCGCGGCTGGGGCTGTGGTTGCCGGAAGTTGAGCGGCGGTAAGTAAGCCGT |
