# Supplementary material for: ATF3 Deficiency Exacerbates Ageing‐Induced Atherosclerosis and Clinical Intervention Strategy
Source: Adv Sci (Weinh). 2025 Jul 11;12(37):e02249. doi: 10.1002/advs.202502249 (PMC12499416; doi:10.1002/advs.202502249)
Supplement: Supplementary file 3 — Supporting Information [file ADVS-12-e02249-s004.docx]

**Supplementary Materials**

**Methods**

**Adeno-associate virus (AAVs) Transfection**

The mouse was secured in a tail vein injection apparatus (Globalebio, Beijing, China), and its tail was disinfected with alcohol. The AAVs carrying the SM22α promoter contains an ATF3 RNAi interference sequence were provided by Genechem (Shanghai, China). Select the cardiovascular tissue-specific serotype AAV9. The control AAV9 and AAV9-ATF3-RNAi were prepared by diluting them with a sterile saline solution to the appropriate titer. The injection volume of the AAVs was 200 μl, with a total titer of 3×10^11^ v.g/ml. The mouse tail was straightened, and the needle was inserted at 1/3 distance from the tail tip. The AAVs were slowly and evenly injected into the mouse tail vein. Gentle pressure was applied with a sterile gauze to stop any bleeding.

**Blood pressure (BP) and pulse wave velocity (PWV) measurements**

The CODA noninvasive tail-cuff BP system (Kent Scientific, CT, USA) was used to measure the BP of the mice. The PWV in mice was determined using a Doppler Flow Velocity System (Indus Instruments, TX, USA). The mice were positioned supine on a heated ECG electrode board maintained at a constant temperature of 38℃. Their limbs were fixed and coated with conductive gel. Continuous electrocardiogram recordings were obtained, and pressure waveforms were detected using an 20 MHz probe at the descending aorta and abdominal aorta. The time intervals for the pressure waves to travel to different blood vessels were calculated over 10 cardiac cycles, and the distance between the descending aorta and abdominal aorta was measured using a ruler. Mouse PWV (m/s) was calculated by dividing the distance traveled by the time taken for propagation.

**Cell culture**

Primary mouse vascular smooth muscle cells (VSMCs) were isolated and cultured from the aorta of C57BL/6 mice. VSMCs that tested negative for mycoplasma (40601ES, Yeasen, China) were used in the latter experiment. The endothelial cell line (zqxzbio,China) was cultured in DMEM supplemented with 10% BI serum. The cells were cultured in Dulbecco's Modified Eagle Medium/Nutrient Mixture F-12 (DMEM/F-12, Gibco, NYC, USA) enriched with 10% fetal bovine serum (Gibco) and 1% penicillin/streptomycin at 37℃ in a humidified atmosphere with 5% CO2. Primary VSMCs used in the experiments were maintained within passages 4-6 to ensure their retention of a youthful phenotypic state. TZ was dissolved in DMSO, TZ1 at 1nM, TZ2 at 10nM and TZ3 at 20nM. Doxorubicin (Doxo, Sigma Aldrich, sigmaaldrich.cn/CN/zh/product/sigma/d1515) was dissolved in DMSO and used to treat cells at 100 nM for 48 h. Rapamycin (RAPA, Sigma Aldrich, sigmaaldrich.cn/CN/zh/product/mm/553210) was dissolved in DMSO and used to treat cells at 50 nM for 48 h. Bafilomycin A1 (BafA1, Sigma Aldrich, sigmaaldrich.cn/CN/zh/product/mm/508409) was dissolved in DMSO and used to treat cells at 100 nM for 6 h. DC-LC3in-D5 (LC3in, MedChemExpress, medchemexpress.cn/dc-lc3in-d5.html) was dissolved in DMSO and used to treat cells at 10 uM for 48 h. Actinomycin D (MedChemExpress, medchemexpress.cn/Actinomycin-D.html) was dissolved in DMSO and used to treat cells at 10 nM for 8 h. Phenoxybenzamine (Phen, MedChemExpress, medchemexpress.cn/phenoxybenzamine.html) was dissolved in DMSO and used to treat cells at 1 ㎛ for 4 h. Doxazosin (DZ, Sigma Aldrich, sigmaaldrich.cn/CN/zh/product/sial/y0000553) was dissolved in DMSO and used to treat cells at 10 nM for 48 h.

**Senescence-associated β-galactosidase (SA-β-gal) staining assay**

The SA-β-gal staining kit (Abbkine, Wuhan, China) was applied to detect the activity of β-galactosidase (β-Gal) in tissues or cells following the protocol provided by the manufacturer [1]. The SA-β-gal-positive proportion area was counted. Subsequently, it was calculated using phase-contrast microscopy. Images were analyzed with Image J software (Version 1.8.0, National Institutes of Health, USA).

**Histological Staining Techniques: Hematoxylin-eosin (HE), Elastica van Gieson (EVG), Oil Red O Staining and Immunofluorescence (IF)**

The fixed vascular tissue was embedded in an OCT embedding medium (Sakura, Japan) and sliced into 5 μm thick transverse sections. HE, EVG and Oil Red O staining were performed as previously reported [2]. Frozen sections were permeabilized with 0.1% Triton® X-100 (BioFroxx 1139ML100) for 15 min. Non-specific binding sites were blocked with 10% serum from the secondary antibody species at 37℃ for 30 min. The primary antibody working solution was prepared and incubated overnight at 4℃. Thawed sections were subsequently incubated with the secondary antibody solution at 37℃ for 1 h. Subsequently, the sections were stained with a DAPI solution for 5 min in the dark to label the nuclei, followed by washing with TBST. The samples were imaged using a confocal laser-scanning microscope (Nikon C2+, Tokyo, Japan). Image-Pro Plus 7.0 software (Media Cybernetics, USA) was used for image analysis.

**Transmission electron microscope (TEM)**

Fresh vascular tissue, not exceeding 1 mm × 1 mm × 1 mm in volume, was promptly immersed in an electron microscope fixative (Baiqiandu Technology, Wuhan, China) and fixed at 4℃ for 2–4 h. Cell clusters, comparable to green beans in size, were isolated, the culture medium was removed, and clusters were subsequently fixed in an electron microscope fixative at 4℃ for 2–4 h. After secondary fixation and dehydration with an alcohol gradient, the samples were infiltrated overnight with a mixture of propylene oxide and an embedding agent (Baiqiandu technology). Following embedding, 70 nm ultrathin sections were cut using a microtome (Daitome, Taiwan, China). These sections were double-stained with uranium and lead and allowed to air dry overnight at room temperature. Observation and image acquisition were conducted under a transmission electron microscope (JEOL, Japan) for subsequent analysis.

**Single-Cell RNA Sequencing (scRNA-seq) data analysis**

Raw sequencing data was downloaded from the Gene Expression Omnibus database (Dataset ID: GSE253903). Using the Seurat4norm algorithm, we standardized the single-cell sequencing data to obtain accurate and unbiased single-cell gene expression data. Subsequently, we applied PCA (Principal Component Analysis) and t-SNE (t-Distributed Stochastic Neighbor Embedding) algorithms for dimensionality reduction. Using the Bioconductor package SingleR, we completed the cell type annotation, and we identified transcription factors in the data with pySCENIC (v0.9.5). For the comparison of differential genes between groups, the parameter was defined as log2|fold-change|>0.25 and p-adjust <0.05.

**Bulk RNA-sequencing**

VSMCs were isolated from fresh mouse aortas. Briefly, vessels were enzymatically digested using collagenase (MedChemExpress) to remove the adventitia, followed by elastase (MedChemExpress) treatment to dissociate the tissue into single cells. The cells labeled with an anti-vWF antibody to identify endothelial cells (ECs). vWF-positive cells were removed using magnetic bead separation (Sigma Aldrich). Total RNA was extracted from VSMCs using the HiPure Total RNA Mini Kit (Magen, Guangzhou, China). RNA sequencing and bioinformatic analyses were performed by BGI Genomics (Shenzhen, China) using the MGISEQ x000 system. Gene expression levels were quantified as transcripts per million (TPMs) using an RSEM simulator. Differentially expressed genes (DEGs) were identified with a significance threshold set at false discovery rate (FDR) <0.05 and |log2 (fold change) | >0.5. Data analysis utilized the limma R package (version 4.1.0).

**Western blotting (WB)**

Total proteins were extracted from VSMCs and vascular tissue using RIPA lysis buffer supplemented with a 100× mixture of phosphatase and protease inhibitor cocktail (Promoter, Wuhan, China). The proteins were transferred onto a PVDF (Polyvinylidene Fluoride) membrane (Millipore, Bedford, MA, USA) following the described procedure[2]. Protein bands were visualized using an enhanced chemiluminescence detection reagent (Beyotime) and a ChemiScope 6100 imaging system (Clinx, Shanghai, China), with image analysis using Image J software (Version 1.8.0).

**Antibodies**

**Table S7** provides a detailed description.

**RNA extraction and real-time quantitative PCR (RT-qPCR) analysis**

Total RNA was extracted from homogenized vascular tissue or VSMCs using Trizol (Thermo Scientific). The concentration and purity of the total RNA were assessed using NanoDrop 2000 (Thermo Scientific) in a C1000 Touch PCR Amplifier (Bio-rad, CA, USA). cDNA synthesis was performed using the reverse transcription kit (Toyobo, Osaka, Japan). The qRT-PCR analysis utilized the StepOne Real-Time PCR system (Quantagene q225, Kubotechnology, Beijing, China) and employed the 2^-ΔΔCt^ method to determine relative RNA levels. **Table S8** provides additional information.

**CUT&Tag library preparation and data analysis**

VSMCs were washed twice gently with wash buffer [3]. 10μL Concanavalin A coated magnetic activated beads (Apexbio, Shanghai, China) were added to each sample and incubated at room temperature for 10 minutes. The cells bound to the beads were then subjected to immunoprecipitation using anti-ATF3 antibodies. To enhance the number of Protein A binding sites, a secondary antibody was included. After washing, the cells were treated with a PAG-Tn5 adapter complex to prepare CUT&Tag fragment DNA libraries. The DNA libraries underwent amplification for 15 cycles and were sequenced on the Illumina NovaSeq Xplus using a paired-end 150 sequencing approach. Raw reads were filtered to obtain clean reads by using fastp (v0.20.0). Clean reads were mapped to the mouse reference genome (mm10) using Bowtie2 (v2.2.6) and subsequently filtered for high-quality BAM files with SAMtools (v1.10). Peaks were identified using MACS2 (v2.2.7.1) with default parameters. HOMER (v4.11) was used to annotate the nearest genes and genomic features associated with the peaks. Differential peaks were detected using DiffBind, applying a cut-off value of log2|fold-change|>1 and p-adjust <0.05.

**Chromatin Immunoprecipitation (ChIP) -qPCR**

Harvested mVSMCs were washed twice with ice-cold PBS, resuspended in 1% formaldehyde, and rotated at room temperature for 10 minutes. The reaction was stopped with Glycine Buffer, followed by two PBS washes. Cell pellets were collected by centrifugation. Cell pellets were lysed with Lysis Buffer for 30 min, followed by addition of ChIP Buffer (Biology, Wuhan, BOLG2309) and thorough mixing. The supernatant was combined with ultrapure water and RNase A, incubated at 37°C for 5 min, then treated with NaCl and Proteinase K for 3 h at 65°C. DNA was purified using a DNA purification kit (ThermoFisher), eluted in ultrapure water, and quantified via Qubit fluorometry, with fragment size analyzed by 1.5% agarose gel electrophoresis. For ChIP-qPCR, samples were divided into Input, IgG, and ATF3-IP groups. A 490 µL diluted sample was incubated with 3–5 µg ATF3 antibody or 1 µL IgG at 4°C for 3 h under rotation. The mixture was transferred to pre-treated magnetic bead tubes and rotated at 4°C for 2 h. Beads were washed five times with Wash Buffer, briefly centrifuged, and the supernatant discarded. DNA was eluted with Elution Buffer, vortexed, and the supernatant treated with RNase A, NaCl, and Proteinase K for 3 h at 65°C. Purified DNA was subjected to qPCR analysis.

**Molecular docking**

The protein structures were generated using Alphafold and manually optimized for various parameters, such as desolvation and hydrogenation, using AutoDockTools-1.5.7. Protein-protein docking was then performed using the GRAMM docking server, followed by further optimization of the resulting complex. Protein-protein interaction predictions were made using PyMOL, categorizing and scoring interaction types based on functional residues of different amino acids. Finally, a protein-protein interaction map was created.

**Molecular dynamics (MD) simulations**

MD were performed using GROMACS-2022. The small molecule's atomic charges were calculated using the AM1-BCC method in Ambertools, with GAFF for the ligand, Amber ff99SB for the protein, and TIP3P for water.The system was energy-minimized (50,000 steps, steepest descent), followed by equilibration with positional restraints on protein heavy atoms (100 ps NVT, 100 ps NPT). A 100-ns production MD was then run without restraints. Simulations maintained 1 bar pressure (Parrinello-Rahman) and 310 K temperature. Long-range electrostatics used PME, with a 1 nm cutoff for van der Waals interactions. Bonds involving hydrogen atoms were constrained using SHAKE. Trajectories were saved every 10 ps.

**Small-interfering RNA (siRNA) transfection**

All siRNA sequences were synthesized by RiboBio (Guangzhou, China). The target sequence for mouse siRNA-ATF3 was 5ʹ-CCTCTTTATCCAACAGATA-3ʹ. The target sequence for mouse siRNA-ATG7 was 5ʹ-CCTGAGAGCATCCCTCTAA-3ʹ. VSMCs were treated with a mixture of the transfection reagent (Invitrogen, Carlsbad, CA, USA) and siRNA for 6–8 h. Subsequently, the medium was replaced with a complete culture medium, and gene expression was observed.

**Adenovirus (AdV) transfection**

When the VSMCs reached 30% confluence, AdV transfection carrying the ATF3 or ATG7 gene (Genechem, Shanghai, China) and control-negative virus was initiated. The medium was replaced with an adenovirus-containing medium and incubated for 18 h. Transfection efficiency was then assessed based on fluorescence.

**Cell viability assay**

Cell viability was assessed using the Cell Proliferation assay kit (Abcam) following established protocols [2]. Absorbance was measured for each sample at 450 nm using a microplate reader (ML31-P, Mhot, Guangzhou, China).

**Cell migration assay**

VSMCs were first seeded in a 6-well plate (Ibidi, Martinsried, Germany), cultured in DMEM supplemented with 10% fetal bovine serum for 24 h, and formed a fusion monolayer. Subsequently, the cells were then grown in serum-free DMEM and incubated in a medium with or without TZ for 36 h. Migration was observed using an inverted microscope (TEx000, Nikon) at specified time points (0, 24, and 36 h). Migration distance was quantified using Image J (V 1.8.0) analysis software.

**Single-Base Elongation- and Ligation-Based qPCR Amplification (SELECT) Assay**

SELECT assay was performed as previously described [4]. Briefly, qPCR was used to measure the *Atf3* RNA levels in each sample, maintaining consistent *Atf3* RNA content across groups. The mixture of total RNA and both upstream and downstream primers was prepared, annealed and extended. Following the combination of DNA polymerase, ATP, and ligase, the mixture was added. Then, single nucleotides were extended and ligated. Finally, qPCR was used to detect the SELECT amplification products.

**RNA immunoprecipitation (RIP) assay**

The RIP experiment was performed following the protocol outlined in the BersinBio (China, Bs5010) RIP kit manual. VSMCs samples were collected, and polysome lysis buffer containing protease inhibitor and RNase inhibitor was added. DNase salt stock and DNase were utilized to eliminate DNA from the samples. Samples were mixed with the YTHDF2 antibody and an equal amount of IgG antibody, then incubated overnight at 4°C using a vertical mixer. Protein A/G beads were added to the samples and mixed vertically at room temperature for 1 h. After washing multiple times with polysome washing buffer, the beads were collected using a magnetic rack. RNA was extracted and qRT-PCR was performed to detect mRNA levels.

**Co-immunoprecipitation (Co-IP) and Proteomics Analysis**

Cells were lysed using a protein lysis buffer (Abbkine Scientific, Wuhan, China) mixed with a protease inhibitor cocktail. The lysates were incubated with the anti-ATF3 antibody (Abcam), anti-ATG7 antibody (Abcam), or IgG with protein A/G magnetic beads overnight at 4℃. Subsequently, the cell lysates were incubated with the magnetic beads overnight at 4℃. Samples were washed to remove unbound immune complexes. In contrast, the bound immune complexes were separated from the magnetic beads using an elution buffer. The remaining immune complexes were collected for mass spectrometry analysis. The proteins obtained from the Co-IP experiment were analyzed by BGI Corporation (Shenzhen, China) for further identification and analysis.

**Glutathione S-transferase (GST) pull-down assay**

Transformed One Shot^TM^ Stbl3^TM^  Chemically Competent *E.* *coli* (C737303, ThermoFisher) expressing the GST-tagged ATF3 fusion vector were induced with 1 mM IPTG (Sigma) at 25℃ for 12 h. GST fusion protein was purified using glutathione agarose resin. The His-tagged ATG7 construct was introduced into a plasmid in Human Embryonic Kidney 293T cells (HEK-293T, WN-10189, Chinese Academy of Sciences, Shanghai, China). GST or GST-ATF3 proteins were purified using BersinBio GSH magnetic beads, and His-tagged ATG7 protein was isolated via His-tag antibody-based purification. These purified proteins were then incubated overnight at 4°C under controlled binding conditions. The resin was washed three times to remove unbound proteins. Protein immunoblotting of the input and output samples was performed for further identification.

**Surface Plasmon Resonance (SPR) screening**

The CM5 sensor chip was first activated. Recombinant ATF3 protein (Cloud-Clone Crop, Wuhan, China) was then diluted to 50 μg/mL concentration and injected into the sample channel. Recombinant ATG7 protein (Cloud-Clone Crop) was diluted to 7 concentrations (100, 50, 25, 12.5, 6.25, 3.125 and 0 nM) and injected into the channels for binding and dissociation, with both processes taking place in the analyte buffer. Six cycles were conducted, ascending in analyte concentration. The BiacoreTx00 platform (Cytiva, Shanghai, China) was used to measure molecular affinity between molecules [5].

**Mutant variant of ATF3**

Wild-type ATF3 and three-point mutant variants, based on the predicted ATF3 and ATG7 binding sites, were constructed by inserting the corresponding sequences into a Flag-tagged plasmid complementary DNA (pCDNA) vector using restriction endonucleases and DNA ligases. **Table S9** provides additional sequence information. The inserted sequences were then verified by sequencing to ensure compatibility with the target. For the Co-IP experiment, ATG7 was cloned and inserted into the pCDNA vector for expression of the fusion protein with a His tag. The Co-IP was performed using anti-Flag antibody with IgG antibody as a negative control, following the same procedure as described above.

**Protein-nucleus separation**

The Cell Nucleus/Cytosol Protein Extraction Kit (Beyotime, Shanghai, China) was used to separate nuclear/cytoplasmic proteins. The cell pellet was resuspended in Cytosol Protein Extraction Reagent A with PMSF and then vigorously vortexed at the highest speed for full dispersion. Subsequently, the mixture was chilled on ice for 15 min, and then Cytosol Protein Extraction Reagent B was added. After vigorous vortexing at the highest speed and another 1-min ice incubation, the supernatant containing the extracted cytoplasmic proteins, was collected by centrifugation. The pellet underwent further treatment with a Nuclear Protein Extraction Reagent, followed by vigorous vortexing at the highest speed for full dispersion. Finally, the supernatant containing the extracted nuclear proteins was collected by centrifugation.

**Dual-Luciferase Reporter Assay**

The ATG7 gene promoter region, including mutant and wild-type sequences, was cloned into the PGL3-basic vector (Genomeditech, Shanghai, China). See **Table S10** for specific sequences. HEK-293T cells were transfected with the constructed vectors using an HG transgene reagent (Genomeditech). Renilla Luciferase-Thymidine Kinase (pRL-TK, TK) was co-transfected into cells as a transfection control. After 48 h, cell samples were collected and subjected to a reporter gene assay using the Promega Dual-Luciferase Reporter Assay Kit (Madison, WI, USA) according to the instructions of the manufacturer [2]. Relative light units were measured with a chemiluminescence plate reader (SpectraMax L, USA).

**Statistical analysis**

The statistical analysis and data visualization were conducted using IBM SPSS Statistics 23.0 and GraphPad Prism 8.0 software. Continuous variables were presented as mean ± standard deviation (SD) from three independent experiments. The normality of data distribution was assessed with the Shapiro-Wilk test. For normally distributed data, unpaired t-tests were used for single-group comparisons, while one-way analysis of variance (ANOVA) followed by Tukey's post-hoc test was used for multiple-group comparisons. Non-normally distributed data were analyzed using the non-parametric Mann–Whitney U test and Kruskal-Wallis test followed by Dunn's multiple comparison test. The correlation test uses Pearson analysis. The Chi-square test was used to evaluate the statistics. Statistical significance was set at P < 0.05.

References:

[1] N.S. Mohamad Kamal, S. Safuan, S. Shamsuddin, P. Foroozandeh, Aging of the cells: Insight into cellular senescence and detection Methods, European journal of cell biology, 99 (2020) 151108.

[2] T. Ji, D. Yan, Y. Huang, M. Luo, Y. Zhang, T. Xu, S. Gao, L. Zhang, L. Ruan, C. Zhang, Fibulin 1, targeted by microRNA-24-3p, promotes cell proliferation and migration in vascular smooth muscle cells, contributing to the development of atherosclerosis in APOE(-/-) mice, Gene, 898 (2024) 148129.

[3] M. Bartosovic, M. Kabbe, G. Castelo-Branco, Single-cell CUT&Tag profiles histone modifications and transcription factors in complex tissues, Nature biotechnology, 39 (2021) 825-835.

[4] X. Liu, J. Yuan, X. Zhang, L. Li, X. Dai, Q. Chen, Y. Wang, ATF3 Modulates the Resistance of Breast Cancer Cells to Tamoxifen through an N(6)-Methyladenosine-Based Epitranscriptomic Mechanism, Chem Res Toxicol, 34 (2021) 1814-1821.

[5] H. Bonnet, L. Coche-Guerente, E. Defrancq, N. Spinelli, A. Van der Heyden, J. Dejeu, Negative SPR Signals during Low Molecular Weight Analyte Recognition, Anal Chem, 93 (2021) 4134-4140.

**Supplementary Figure Legends**

Figure S1: Vascular smooth muscle cells (VSMCs) in atherosclerosis (AS) simultaneously display signs of senescence and a decline in ATF3 content. A: Molecular structure of TZ. B: Analysis of TZ purity using mass spectrometry. C: Dot plots displaying the expression of representative marker genes with an AUC cutoff of ≥ 0.8 for each cell type. D: The violin plot illustrating the variation in levels of AP-1/ATF family transcription factors across different cell types. E: Immunofluorescent images of p21 (red), SM22α (red), OPN (red), α-SMA (green) and nuclei (blue) in human carotid arteries (scale bars = 20 μm; n = 5). Error bars represent mean ± standard deviation. The unpaired t-test were used to compare data; *p < 0.05, **p < 0.01, ***p < 0.001.

Figure S2: Vascular smooth muscle cells (VSMCs) in atherosclerosis (AS) simultaneously display signs of senescence and a decline in ATF3 content. A: Immunofluorescent images of ATF3 (red), α-SMA (green) and nuclei (blue) in APOE^-/-^ mice aortas (scale bars = 20 μm; n = 5). B: Immunofluorescent images of p53 (red), p21 (red), SM22α (red), OPN (red), α-SMA (green) and nuclei (blue) in APOE^-/-^ mice aortas (scale bars = 20 μm; n = 5). C: qPCR analysis of *p53, p21*, and *Atf3* expression in aortic tissues from mice across age groups (n = 5). D: Western blot analysis of p53, p21, and ATF3 expression in aortic tissues from mice across age groups (n = 5). E: At different time periods, qPCR analysis of *Atf3* mRNA expression in VSMC specifically knock down ATF3 mice carotid arteries (n = 5). F: At different time periods, western blot analysis of ATF3 expression in VSMC specifically knock down ATF3 mice carotid arteries (n = 5). Error bars represent mean ± standard deviation. The unpaired t-test (A,B) and one-way ANOVA (C-F) were used to compare data; *p < 0.05, **p < 0.01, ***p < 0.001.

Figure S3: Specific knockdown of ATF3 in ApoE^-/-^ mice VSMCs exacerbates cellular senescence and atherosclerosis. A: At different time periods, IF imaging of ATF3 (red), α-SMA (green), and nuclei (blue) in VSMC specifically knock down ATF3 mice carotid arteries (scale bars = 20 μm; n = 5). B: Oil Red O staining of aortic root sections of SAMR1 and SAMP8 mice (scale bars = 100 μm; n = 5). C: qPCR analysis of *Il-6, Vcam-1 and Tnf* mRNA expression in SAMR1 and SAMP8 mice (n=5). D: EVG staining and TEM of mouse aortas (scale bars, 50 μm, 100 μm, 1 μm, and 500 nm). EF: elastic fiber, CF: collagen fiber; arrows indicate areas of collagen fiber disarray (n = 5). E: Western blot analysis of SM22α and OPN expression in ApoE-/- mice aortas (n = 5). F: IF imaging of SM22α (red), OPN (red), α-SMA (green), and nuclei (blue) in ApoE-/- mice aortas (scale bars = 20 μm; n = 5). Error bars represent mean ± standard deviation. The unpaired t-test (A-C) and one-way ANOVA (D-F) were used to compare data; *p < 0.05, **p < 0.01, ***p < 0.001.

Figure S4: A: Feature plot illustrating the expression distribution of ATF3 and ATG7 in VSMCs. B: Scatter plot illustrating the correlation between ATG7 and ATF3 expression levels. C–K: Silencing ATF3 using siRNA transfection in VSMCs. C: qPCR analysis of *Atf3* mRNA expression in VSMCs (n = 3). D: Western blot analysis of ATF3 expression in VSMCs (n = 3). C–F: Immunofluorescent staining of ATF3 (red) and nuclei (blue) in VSMCs (scale bars, 20 μm; n = 3). G: qPCR analysis of *Atg7* mRNA expression levels in VSMCs (n = 3). H: Western blot analysis of SQSTM1 and ATG7 expression in VSMCs (n = 3). I: Western blot analysis of LC3B-II/LC3B-I in VSMCs. VSMCs treated with BafA1 (100 nM) for 6 h were used as a positive control for impaired autophagic flux (n = 3). J–K: Silencing ATF3 using siRNA transfection in VSMCs. L: Quantification of the affinity between ATG7 and ATF3 interaction using SPR analysis. Error bars represent mean **±** standard deviation. The Mann–Whitney U-test (C–E, G, I) and Kruskal–Wallis test (K) were performed to compare data; *p < 0.05, **p < 0.01, ***p < 0.001.

Figure S5: A: qPCR analysis of *Atg7* mRNA expression in VSMCs (n = 3). B: Immunofluorescent staining of ATG7 (red) and nuclei (blue) in VSMCs (scale bars, 20 μm; n = 3). C-D: Western blot analysis of ATG7 expression in VSMCs (n = 3). E-F: Based on nuclear-cytoplasmic fractionation assays, overexpression of ATG7 was confirmed to promote increased nuclear translocation of ATF3 (n=3). G: Fluorescence analysis of ATF3 and ATG7 distribution in the nucleus and cytoplasm (scale bars, 20 μm; n = 3). Error bars represent the mean ± standard deviation. The Mann–Whitney U-test was performed to compare data; **p < 0.01.

Figure S6: TZ disrupts the stability of the *Atf3* mRNA-YTHDF2 complex. A: Structural backbone Rg variations from molecular dynamics simulations. B: Structural backbone RMSF variations from molecular dynamics simulations. C: Free energy landscape of the complex. D: MM-PBSA-derived binding free energy values.

Figure S7: A: The effect of TZ on *Atf3* mRNA expression levels in endothelial cells (ECs) was examined by qPCR (n = 3). B: TZ has no effect on the half-life of *Atf3* mRNA in ECs (n = 3). C: Relative m6A methylation abundances of *Atf3* mRNA at 1627 site measured by the SELECT method in ECs (n = 3). D: Dot plot of the GO pathway enrichment analysis of differentially expressed genes between SAMP8 and SAMP8+TZ group. E: qPCR analysis of AP-1/ATF family transcription factors mRNA expression levels in between SAMP8 and SAMP8+TZ group (n = 5). The unpaired t-test (E) and Kruskal–Wallis test (A-C) were used to compare data; *p < 0.05, **p < 0.01, ***p < 0.001.

Figure S8: TZ improves VSMC senescence and phenotypic transition in SAMP8 mice. A: Hematoxylin and eosin staining of mouse livers and kidneys (scale bars = 5 mm; n = 5). B, C: SBP and DBP in SAMR1 mice and SAMP8 mice after three months of TZ oral administration (TZ1: 20 μg/kg, TZ2: 60 μg/kg, TZ3: 180 μg/kg; n = 6). D–H: qPCR analysis of *Atf3, p53, p21*, *Sm22*α and *Opn* (n = 5). I–K: Western blot analysis of SM22α and OPN expression in mouse aortas (n = 5). L: Immunofluorescent images of SM22α (red), α-SMA (green), and nuclei (blue) in mouse aortas (scale bars = 20 μm; n = 5). Error bars represent mean ± standard deviation. The one-way ANOVA was used to compare data; *p < 0.05, **p < 0.01, ***p < 0.001.

Figure S9: TZ improves VSMC phenotypic transition in SAMP8 mice. A: Immunofluorescent images of OPN (red), α-SMA (green), and nuclei (blue) in mouse aortas (scale bars = 20 μm; n = 5). B: HE staining, EVG staining and TEM of mouse aortas (scale bars, 50 μm, 100 μm, 1 μm, and 500 nm). EF: elastic fiber, CF: collagen fiber; arrows indicate areas of collagen fiber disarray (n = 5). Error bars represent mean ± standard deviation. The one-way ANOVA was performed to compare data; *p < 0.05, **p < 0.01, ***p < 0.001.

Figure S10: TZ reverses VSMC senescence and phenotype conversion via ATF3. A: Immunofluorescent staining of ATF3 (red), α-SMA (pink), CD31(orange) and nuclei (blue) in mouse aortas (scale bars = 20 μm; n = 5). B: Hematoxylin and eosin staining of mouse livers and kidneys (scale bars = 5 mm; n = 5). C: qPCR analysis of *p53, p21*, *Sm22*α and *Opn* (n = 5). D: Western blot analysis of SM22α and OPN expression in mouse aortas (n = 5). E: Immunofluorescent images of SM22α (red), OPN (red), α-SMA (green), and nuclei (blue) in mouse aortas (scale bars = 20 μm; n = 5). Error bars represent mean ± standard deviation. The one-way ANOVA was used to compare data; *p < 0.05, **p < 0.01, ***p < 0.001.

Figure S11: Silencing ATF3 using siRNA transfection in VSMCs. A: SA-β-gal staining of VSMCs (scale bars, 50 μm; n = 3). B–C: qPCR analysis of *p53* and *p21* mRNA expression levels in VSMCs (n = 3). D: Western blot analysis of p53 and p21 expression in VSMCs (n = 3). E–F: qPCR analysis of *Sm22α* (M) and *Opn* (N) mRNA expression levels in VSMCs (n = 3). G: Western blot analysis of SM22α, and OPN expression in VSMCs (n = 3). H: Immunofluorescent staining of SM22α (red), OPN (red) and nuclei (blue) in VSMCs (scale bars, 20 μm; n = 3). Error bars represent mean **±** standard deviation. The Kruskal–Wallis test was performed to compare data; *p < 0.05, **p < 0.01, ***p < 0.001.

Figure S12: TZ improves autophagy in an ATF3 dependent manner. A: qPCR analysis of *Atg7* mRNA expression levels in VSMCs (n = 3). B–D: Western blot analysis of SQSTM1 and ATG7 in VSMCs (n = 3). E–F: Western blot analysis of LC3B-II/LC3B-I in VSMCs. VSMCs treated with BafA1 (100 nM) for 6 h were used as a positive control for impaired autophagic flux (n = 3). G: TEM observation of autophagic phenomena in VSMCs, with arrows indicating autophagosomes or autolysosomes (scale bars, 2 μm and 500 nm; n = 3). H: Fluorescence analysis of VSMCs transfected with mCherry-GFP-LC3 reporter (n = 3); red: autophagosomes, yellow: autolysosomes (scale bars, 10 μm). Error bars represent mean ± standard deviation. The Kruskal–Wallis test was performed to compare data; *p < 0.05, **p < 0.01.

Figure S13: Silencing ATG7 using siRNA transfection in VSMCs. A: SA-β-gal staining of VSMCs (scale bars = 50 μm; n = 3). B–E: qPCR analysis of *p53, p21*, *Sm22*α and *Opn* in VSMCs (n = 3). F–G: Western blot analysis of p53, p21, SM22α, and OPN expression in VSMCs (n = 3). H–I: Immunofluorescent staining of SM22α (red), OPN (red), and nuclei (blue) in VSMCs (scale bars, 20 μm; n = 3). Error bars represent mean ± standard deviation. The Kruskal–Wallis test was performed to compare data;*p < 0.05, **p < 0.01, ***p < 0.001.

Figure S14: VSMCs treated with LC3in (10 μM) for 48 h inhibit LC3 lipidation. A: SA-β-gal staining of VSMCs (scale bars = 50 μm; n = 3). B–E: qPCR analysis of *p53, p21*, *Sm22*α and *Opn* in VSMCs (n = 3). F–G: Western blot analysis of p53, p21, SM22α, and OPN expression in VSMCs (n = 3). H–I: Immunofluorescent staining of SM22α (red), OPN (red), and nuclei (blue) in VSMCs (scale bars, 20 μm; n = 3). Error bars represent mean ± standard deviation. The Kruskal–Wallis test was performed to compare data;*p < 0.05, **p < 0.01, ***p < 0.001.

Figure S15: TZ improves VSMC senescence and age-related phenotypes in APOE^-/-^ mice. A–C: qPCR analysis of Atf3, *p53* and *p21* mRNA expression levels in mouse carotid arteries (n = 5). D–E: EVG staining of mouse aortas (scale bars, 50 μm, 100 μm). F: SBP and DBP in APOE^-/-^ mice after three months of TZ. H–I: qPCR analysis of *Sm22α* and *Opn* mRNA expression levels in mouse carotid arteries (n = 5). J–K: Western blot analysis of SM22α and OPN expression in mouse aortas (n = 5). L–M: Immunofluorescent staining of SM22α (red), OPN (red), α-SMA (green) and nuclei (blue) in mouse aortas (scale bars, 20 μm; n = 5). Error bars represent mean ± standard deviation. The one-way ANOVA was performed to compare data; *p < 0.05, **p < 0.01, ***p < 0.001.

Figure S16: TZ improves atherosclerosis by modulating ATF3 activity. A: qPCR analysis of *Atg7* mRNA expression levels in APOE^-/-^ mice carotid arteries (n = 5). B: Immunofluorescent staining of ATF3 (red), ATG7 (green), α-SMA (pink) and nuclei (blue) in APOE^-/-^ mice aortas (scale bars, 20 μm; n = 5). C: TEM observation of autophagic phenomena in vascular medial smooth muscle cells (SMCs); arrows indicate autophagosomes or autolysosomes (scale bars, 1 μm and 500 nm; n = 5). Error bars represent mean ± standard deviation. The Kruskal–Wallis was performed to compare data; *p < 0.05, **p < 0.01, ***p < 0.001.
