# Supplementary material for: ATF3 Deficiency Exacerbates Ageing‐Induced Atherosclerosis and Clinical Intervention Strategy
Source: Adv Sci (Weinh). 2025 Jul 11;12(37):e02249. doi: 10.1002/advs.202502249 (PMC12499416; doi:10.1002/advs.202502249)
Supplement: Supplementary file 4 — Supporting Information [file ADVS-12-e02249-s001.docx]

**Supplementary Figure Legends**

Figure S1: **Vascular smooth muscle cells (VSMCs) in atherosclerosis (AS) simultaneously display signs of senescence.** A: Molecular structure of TZ. B: Analysis of TZ purity using mass spectrometry. C: Dot plots displaying the expression of representative marker genes with an AUC cutoff of ≥ 0.8 for each cell type. D: The violin plot illustrating the variation in levels of AP-1/ATF family transcription factors across different cell types. E: Immunofluorescent images of p21 (red), SM22α (red), OPN (red), α-SMA (green) and nuclei (blue) in human carotid arteries (scale bars = 20 μm; n = 5). Error bars represent mean ± standard deviation. The unpaired t-test were used to compare data; *p < 0.05, **p < 0.01, ***p < 0.001.

Figure S2: **Vascular smooth muscle cells (VSMCs) in atherosclerosis (AS) exhibit cellular senescence and reduced ATF3 expression.** A: Immunofluorescent images of ATF3 (red), α-SMA (green) and nuclei (blue) in APOE^-/-^ mice aortas (scale bars = 20 μm; n = 5). B: Immunofluorescent images of p53 (red), p21 (red), SM22α (red), OPN (red), α-SMA (green) and nuclei (blue) in APOE^-/-^ mice aortas (scale bars = 20 μm; n = 5). C: qPCR analysis of *p53, p21*, and *Atf3* expression in aortic tissues from mice across age groups (n = 5). D: Western blot analysis of p53, p21, and ATF3 expression in aortic tissues from mice across age groups (n = 5). E: At different time periods, qPCR analysis of *Atf3* mRNA expression in VSMC specifically knock down ATF3 mice carotid arteries (n = 5). F: At different time periods, western blot analysis of ATF3 expression in VSMC specifically knock down ATF3 mice carotid arteries (n = 5). Error bars represent mean ± standard deviation. The unpaired t-test (A,B) and one-way ANOVA (C-F) were used to compare data; *p < 0.05, **p < 0.01, ***p < 0.001.

Figure S3: **Specific knockdown of ATF3 in ApoE^-/-^ mice VSMCs exacerbates cellular senescence and atherosclerosis.** A: At different time periods, IF imaging of ATF3 (red), α-SMA (green), and nuclei (blue) in VSMC specifically knock down ATF3 mice carotid arteries (scale bars = 20 μm; n = 5). B: Oil Red O staining of aortic root sections of SAMR1 and SAMP8 mice (scale bars = 100 μm; n = 5). C: qPCR analysis of *Il-6, Vcam-1 and Tnf* mRNA expression in SAMR1 and SAMP8 mice (n=5). D: EVG staining and TEM of mouse aortas (scale bars, 50 μm, 100 μm, 1 μm, and 500 nm). EF: elastic fiber, CF: collagen fiber; arrows indicate areas of collagen fiber disarray (n = 5). E: Western blot analysis of SM22α and OPN expression in ApoE-/- mice aortas (n = 5). F: IF imaging of SM22α (red), OPN (red), α-SMA (green), and nuclei (blue) in ApoE-/- mice aortas (scale bars = 20 μm; n = 5). Error bars represent mean ± standard deviation. The unpaired t-test (A-C) and one-way ANOVA (D-F) were used to compare data; *p < 0.05, **p < 0.01, ***p < 0.001.

Figure S4: **Knockdown of ATF3 in VSMCs leads to reduced ATG7 protein levels.** A: Feature plot illustrating the expression distribution of ATF3 and ATG7 in VSMCs. B: Scatter plot illustrating the correlation between ATG7 and ATF3 expression levels. C–K: Silencing ATF3 using siRNA transfection in VSMCs. C: qPCR analysis of *Atf3* mRNA expression in VSMCs (n = 3). D: Western blot analysis of ATF3 expression in VSMCs (n = 3). C–F: Immunofluorescent staining of ATF3 (red) and nuclei (blue) in VSMCs (scale bars, 20 μm; n = 3). G: qPCR analysis of *Atg7* mRNA expression levels in VSMCs (n = 3). H: Western blot analysis of SQSTM1 and ATG7 expression in VSMCs (n = 3). I: Western blot analysis of LC3B-II/LC3B-I in VSMCs. VSMCs treated with BafA1 (100 nM) for 6 h were used as a positive control for impaired autophagic flux (n = 3). J–K: Silencing ATF3 using siRNA transfection in VSMCs. L: Quantification of the affinity between ATG7 and ATF3 interaction using SPR analysis. Error bars represent mean **±** standard deviation. The Mann–Whitney U-test (C–E, G, I) and Kruskal–Wallis test (K) were performed to compare data; *p < 0.05, **p < 0.01, ***p < 0.001.

Figure S5: **ATG7 facilitates nuclear translocation of ATF3.** A: qPCR analysis of *Atg7* mRNA expression in VSMCs (n = 3). B: Immunofluorescent staining of ATG7 (red) and nuclei (blue) in VSMCs (scale bars, 20 μm; n = 3). C-D: Western blot analysis of ATG7 expression in VSMCs (n = 3). E-F: Based on nuclear-cytoplasmic fractionation assays, overexpression of ATG7 was confirmed to promote increased nuclear translocation of ATF3 (n=3). G: Fluorescence analysis of ATF3 and ATG7 distribution in the nucleus and cytoplasm (scale bars, 20 μm; n = 3). Error bars represent the mean ± standard deviation. The Mann–Whitney U-test was performed to compare data; **p < 0.01.

Figure S6: **TZ disrupts the stability of the *Atf3* mRNA-YTHDF2 complex.** A: Structural backbone Rg variations from molecular dynamics simulations. B: Structural backbone RMSF variations from molecular dynamics simulations. C: Free energy landscape of the complex. D: MM-PBSA-derived binding free energy values.

Figure S7: **TZ does not affect ATF3 mRNA stability in endothelial cells (ECs) and induces alterations in the ATF3 signaling pathway in VSMCs.** A: The effect of TZ on *Atf3* mRNA expression levels in ECs was examined by qPCR (n = 3). B: TZ has no effect on the half-life of *Atf3* mRNA in ECs (n = 3). C: Relative m6A methylation abundances of *Atf3* mRNA at 1627 site measured by the SELECT method in ECs (n = 3). D: Dot plot of the GO pathway enrichment analysis of differentially expressed genes between SAMP8 and SAMP8+TZ group. E: qPCR analysis of AP-1/ATF family transcription factors mRNA expression levels in between SAMP8 and SAMP8+TZ group (n = 5). The unpaired t-test (E) and Kruskal–Wallis test (A-C) were used to compare data; *p < 0.05, **p < 0.01, ***p < 0.001.

Figure S8: **TZ improves VSMC senescence and phenotypic transition in SAMP8 mice.** A: Hematoxylin and eosin staining of mouse livers and kidneys (scale bars = 5 mm; n = 5). B, C: SBP and DBP in SAMR1 mice and SAMP8 mice after three months of TZ oral administration (TZ1: 20 μg/kg, TZ2: 60 μg/kg, TZ3: 180 μg/kg; n = 6). D–H: qPCR analysis of *Atf3, p53, p21*, *Sm22*α and *Opn* (n = 5). I–K: Western blot analysis of SM22α and OPN expression in mouse aortas (n = 5). L: Immunofluorescent images of SM22α (red), α-SMA (green), and nuclei (blue) in mouse aortas (scale bars = 20 μm; n = 5). Error bars represent mean ± standard deviation. The one-way ANOVA was used to compare data; *p < 0.05, **p < 0.01, ***p < 0.001.

Figure S9: **TZ improves VSMC phenotypic transition in SAMP8 mice.** A: Immunofluorescent images of OPN (red), α-SMA (green), and nuclei (blue) in mouse aortas (scale bars = 20 μm; n = 5). B: HE staining, EVG staining and TEM of mouse aortas (scale bars, 50 μm, 100 μm, 1 μm, and 500 nm). EF: elastic fiber, CF: collagen fiber; arrows indicate areas of collagen fiber disarray (n = 5). Error bars represent mean ± standard deviation. The one-way ANOVA was performed to compare data; *p < 0.05, **p < 0.01, ***p < 0.001.

Figure S10: **TZ reverses VSMC senescence and phenotype conversion via ATF3.** A: Immunofluorescent staining of ATF3 (red), α-SMA (pink), CD31(orange) and nuclei (blue) in mouse aortas (scale bars = 20 μm; n = 5). B: Hematoxylin and eosin staining of mouse livers and kidneys (scale bars = 5 mm; n = 5). C: qPCR analysis of *p53, p21*, *Sm22*α and *Opn* (n = 5). D: Western blot analysis of SM22α and OPN expression in mouse aortas (n = 5). E: Immunofluorescent images of SM22α (red), OPN (red), α-SMA (green), and nuclei (blue) in mouse aortas (scale bars = 20 μm; n = 5). Error bars represent mean ± standard deviation. The one-way ANOVA was used to compare data; *p < 0.05, **p < 0.01, ***p < 0.001.

Figure S11: **Knockdown of ATF3 reverses the anti-senescence activity of TZ in VSMCs.** A: SA-β-gal staining of VSMCs (scale bars, 50 μm; n = 3). B–C: qPCR analysis of *p53* and *p21* mRNA expression levels in VSMCs (n = 3). D: Western blot analysis of p53 and p21 expression in VSMCs (n = 3). E–F: qPCR analysis of *Sm22α* (M) and *Opn* (N) mRNA expression levels in VSMCs (n = 3). G: Western blot analysis of SM22α, and OPN expression in VSMCs (n = 3). H: Immunofluorescent staining of SM22α (red), OPN (red) and nuclei (blue) in VSMCs (scale bars, 20 μm; n = 3). Error bars represent mean **±** standard deviation. The Kruskal–Wallis test was performed to compare data; *p < 0.05, **p < 0.01, ***p < 0.001.

Figure S12: **TZ improves autophagy in an ATF3 dependent manner.** A: qPCR analysis of *Atg7* mRNA expression levels in VSMCs (n = 3). B–D: Western blot analysis of SQSTM1 and ATG7 in VSMCs (n = 3). E–F: Western blot analysis of LC3B-II/LC3B-I in VSMCs. VSMCs treated with BafA1 (100 nM) for 6 h were used as a positive control for impaired autophagic flux (n = 3). G: TEM observation of autophagic phenomena in VSMCs, with arrows indicating autophagosomes or autolysosomes (scale bars, 2 μm and 500 nm; n = 3). H: Fluorescence analysis of VSMCs transfected with mCherry-GFP-LC3 reporter (n = 3); red: autophagosomes, yellow: autolysosomes (scale bars, 10 μm). Error bars represent mean ± standard deviation. The Kruskal–Wallis test was performed to compare data; *p < 0.05, **p < 0.01.

Figure S13: **The anti-senescent effect of TZ is mediated via autophagy activation.** A: SA-β-gal staining of VSMCs (scale bars = 50 μm; n = 3). B–E: qPCR analysis of *p53, p21*, *Sm22*α and *Opn* in VSMCs (n = 3). F–G: Western blot analysis of p53, p21, SM22α, and OPN expression in VSMCs (n = 3). H–I: Immunofluorescent staining of SM22α (red), OPN (red), and nuclei (blue) in VSMCs (scale bars, 20 μm; n = 3). Error bars represent mean ± standard deviation. The Kruskal–Wallis test was performed to compare data;*p < 0.05, **p < 0.01, ***p < 0.001.

Figure S14: **The anti-senescent efficacy of TZ requires functional autophagy.** A: SA-β-gal staining of VSMCs (scale bars = 50 μm; n = 3). B–E: qPCR analysis of *p53, p21*, *Sm22*α and *Opn* in VSMCs (n = 3). F–G: Western blot analysis of p53, p21, SM22α, and OPN expression in VSMCs (n = 3). H–I: Immunofluorescent staining of SM22α (red), OPN (red), and nuclei (blue) in VSMCs (scale bars, 20 μm; n = 3). Error bars represent mean ± standard deviation. The Kruskal–Wallis test was performed to compare data;*p < 0.05, **p < 0.01, ***p < 0.001.

Figure S15: **TZ improves VSMC senescence and age-related phenotypes in APOE^-/-^ mice.** A–C: qPCR analysis of Atf3, *p53* and *p21* mRNA expression levels in mouse carotid arteries (n = 5). D–E: EVG staining of mouse aortas (scale bars, 50 μm, 100 μm). F: SBP and DBP in APOE^-/-^ mice after three months of TZ. H–I: qPCR analysis of *Sm22α* and *Opn* mRNA expression levels in mouse carotid arteries (n = 5). J–K: Western blot analysis of SM22α and OPN expression in mouse aortas (n = 5). L–M: Immunofluorescent staining of SM22α (red), OPN (red), α-SMA (green) and nuclei (blue) in mouse aortas (scale bars, 20 μm; n = 5). Error bars represent mean ± standard deviation. The one-way ANOVA was performed to compare data; *p < 0.05, **p < 0.01, ***p < 0.001.

Figure S16: **TZ improves atherosclerosis by modulating ATF3 activity.** A: qPCR analysis of *Atg7* mRNA expression levels in APOE^-/-^ mice carotid arteries (n = 5). B: Immunofluorescent staining of ATF3 (red), ATG7 (green), α-SMA (pink) and nuclei (blue) in APOE^-/-^ mice aortas (scale bars, 20 μm; n = 5). C: TEM observation of autophagic phenomena in vascular medial smooth muscle cells (SMCs); arrows indicate autophagosomes or autolysosomes (scale bars, 1 μm and 500 nm; n = 5). Error bars represent mean ± standard deviation. The Kruskal–Wallis was performed to compare data; *p < 0.05, **p < 0.01, ***p < 0.001.

**Supplymentary Table Captions:**

Table S1. Carotid surgery patient information

Table S2. 16 proteins enriched in the autophagy pathway

Table S3. The binding site between protein ATF3 and protein ATG7

Table S4. The top ten molecules with the highest binding energy in molecular docking

Table S5. Basic characteristics of SAMR1 and SAMP8 mice

Table S6. Basic characteristics of SAMR1 and SAMP8 mice

Table S7. Primary antibodies applied in IF, WB, IP and RIP

Table S8. Primers applied in qRT-PCR

Table S9. Wild-type and mutation sequence of ATF3 applied in Co-IP

Table S10. Wild-type and mutation sequence of *Atg7* promoter applied in Dual-Luciferase Reporter Assay
